# SOX2 regulates foregut squamous epithelial homeostasis and is lost during Barrett’s esophagus development

**DOI:** 10.1172/JCI190374

**Published:** 2025-06-19

**Authors:** Ramon U. Jin, Yuanwei Xu, T. Mamie Lih, Yang-Zhe Huang, Toni M. Nittolo, Blake E. Sells, Olivia M. Dres, Jean S. Wang, Qing K. Li, Hui Zhang, Jason C. Mills

**Affiliations:** 1 Division of Oncology, Department of Medicine and; 2 Division of Gastroenterology, Department of Medicine, Washington University, St. Louis, Missouri, USA.; 3 Department of Pathology, Johns Hopkins University School of Medicine, Baltimore, Maryland, USA.; 4 Department of Chemical and Biomolecular Engineering, Johns Hopkins University, Baltimore, Maryland, USA.; 5 Section of Gastroenterology, Department of Medicine, Baylor College of Medicine, Houston, Texas, USA.; 6 Department of Oncology and; 7 Department of Urology, Johns Hopkins University School of Medicine, Baltimore, Maryland, USA.; 8 Department of Pathology & Immunology, Baylor College of Medicine, Houston, Texas, USA.; 9 Department of Molecular and Cellular Biology, Baylor College of Medicine, Houston, Texas, USA.

**Keywords:** Development, Gastroenterology, Gastric cancer

## Abstract

Esophageal adenocarcinoma is increasingly prevalent and is thought to arise from Barrett’s esophagus (BE), a metaplastic condition in which chronic acid and bile reflux transforms the esophageal squamous epithelium into a gastric-intestinal glandular mucosa. The molecular determinants driving this metaplasia are poorly understood. We developed a human BE organoid biobank that recapitulates BE’s molecular heterogeneity. Bulk and single-cell transcriptomics, supported by patient tissue analysis, revealed that BE differentiation reflects a balance between SOX2 (foregut/esophageal) and CDX2 (hindgut/intestinal) transcription factors. Using squamous-specific inducible *Sox2*-KO (*Krt5^CreER/+^ Sox2*^Δ/Δ^ *ROSA26^tdTomato/+^*) mice, we observed increased basal proliferation, reduced squamous differentiation, and expanded metaplastic glands at the squamocolumnar junction, some tracing back to *Krt5*-expressing cells. CUT&RUN analysis showed SOX2 bound and promoted differentiation-associated targets (e.g., *Krt13*) and repressed proliferation-associated targets (e.g., *Mki67*). Thus, SOX2 is critical for foregut squamous epithelial differentiation, and its decreased expression is likely an initiating step in progression to BE and then to esophageal adenocarcinoma.

## Abstract 2

SOX2 is a master regulator of upper GI squamous epithelial identity, and its loss is involved in the transcriptional reprogramming seen in Barrett’s esophagus development.

## Graphical abstract

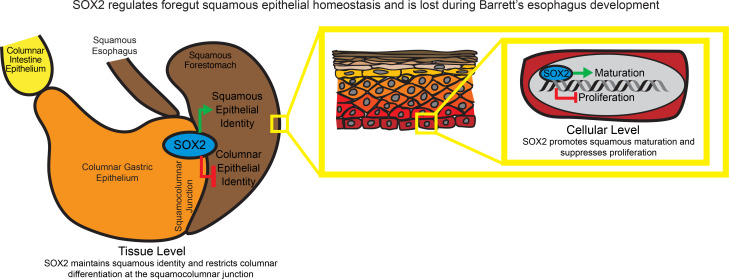

## Introduction

Esophageal adenocarcinoma has increased in incidence over several decades in the United States and is now the predominant histological form of esophageal cancer in the developed world (1–6). Esophageal adenocarcinoma is thought to arise from Barrett’s esophagus (BE), a metaplastic condition caused by chronic gastroesophageal reflux (7), where normal esophageal squamous epithelium is replaced by glandular columnar epithelium (8–10). BE is a precancerous lesion that may progress through low- and high-grade dysplasia to esophageal adenocarcinoma over 5 to 15 years (11–17). As esophageal adenocarcinoma incidence has increased, so has BE, now affecting an estimated 5%–20% of the US population (5, 18–24). Although BE is thought to be a prerequisite for esophageal adenocarcinoma, only a small fraction of people with BE develop esophageal adenocarcinoma (25). Currently, there are no clinically available biomarkers to stratify the cancer risk (26) or effective chemopreventative strategies (27, 28). A better understanding of the molecular events leading to BE development should result in improved diagnostic, early detection, and treatment options for esophageal adenocarcinoma.

BE represents a developmental caudalization, where the proximal (rostral) esophageal epithelium is replaced by epithelium characteristic of more distal (caudal) alimentary tract. Namely, nondysplastic BE lesions often contain a mix of gastric and intestinal cell types (29, 30). Consistent with a process of sequential caudalization, there is epigenetic, genomic, and transcriptional evidence that the epithelium that initially replaces normal esophageal squamous epithelium is of gastric phenotype and/or originates from gastric epithelium migrating from the gastroesophageal junction (31, 32). Given that development of the epithelium in each anatomic region depends ultimately on the expression patterns of pioneering transcription factors (33), understanding how BE forms and progresses will depend on understanding the changes in transcriptional regulation that occur as BE develops, and aberrant transcriptional programming is likely at the root of BE. SOX2, essential for esophageal identity, is highly expressed during embryonic development (34, 35) and adulthood to maintain esophageal homeostasis (36–38). CDX1/2 are critical regulators of intestinal differentiation (39, 40) but are absent in the normal foregut. Thus, the activity of these SOX2 and CDX factors can help characterize patterns of intestinal metaplasia in the stomach (41–43) and in the esophagus (44–47). Chronic inflammation and cytokine stimulation can induce aberrant esophageal activation of CDX2 (46, 48–51). However, ectopic expression of CDX2 by itself in the murine esophageal squamous epithelium does not result in “intestinalization” (52, 53). Thus, BE likely arises through a coordinated progressive transcriptional reprogramming that shifts epithelial differentiation toward a more posterior gastrointestinal phenotype (52–57).

We previously showed that SOX2 is robustly expressed in normal esophageal squamous epithelium and decreases during BE development and esophageal adenocarcinoma progression (30). Here, we established a patient-derived BE organoid biobank to recapitulate the heterogeneity of BE and used single-cell transcriptomics to show that SOX2 regulates a cell division program in BE cells. To assess SOX2’s functional role, we analyzed *Sox2*^Δ/Δ^ mice with induced foregut squamous-specific *Sox2* deletion. These mice exhibited increased basal layer proliferation, decreased mature squamous structural protein expression, and activation of a squamous damage response program. Cleavage under targets and release using nuclease (CUT&RUN) revealed that SOX2 directly activates genes involved in squamous maturation and represses those driving proliferation and signaling. Notably, *Sox2*^Δ/Δ^ mice developed expanded columnar glands at the squamocolumnar junction. In-depth histological and proteomic characterization of these expanded glands showed that some of these glands were derived by reprogramming from *Sox2*^Δ/Δ^ squamous epithelium, and that they exhibited BE markers with both gastric and intestinal characteristics. Together, these findings suggest SOX2 loss is a key event in the transition from squamous to glandular epithelium in BE.

## Results

**Figure 1:**
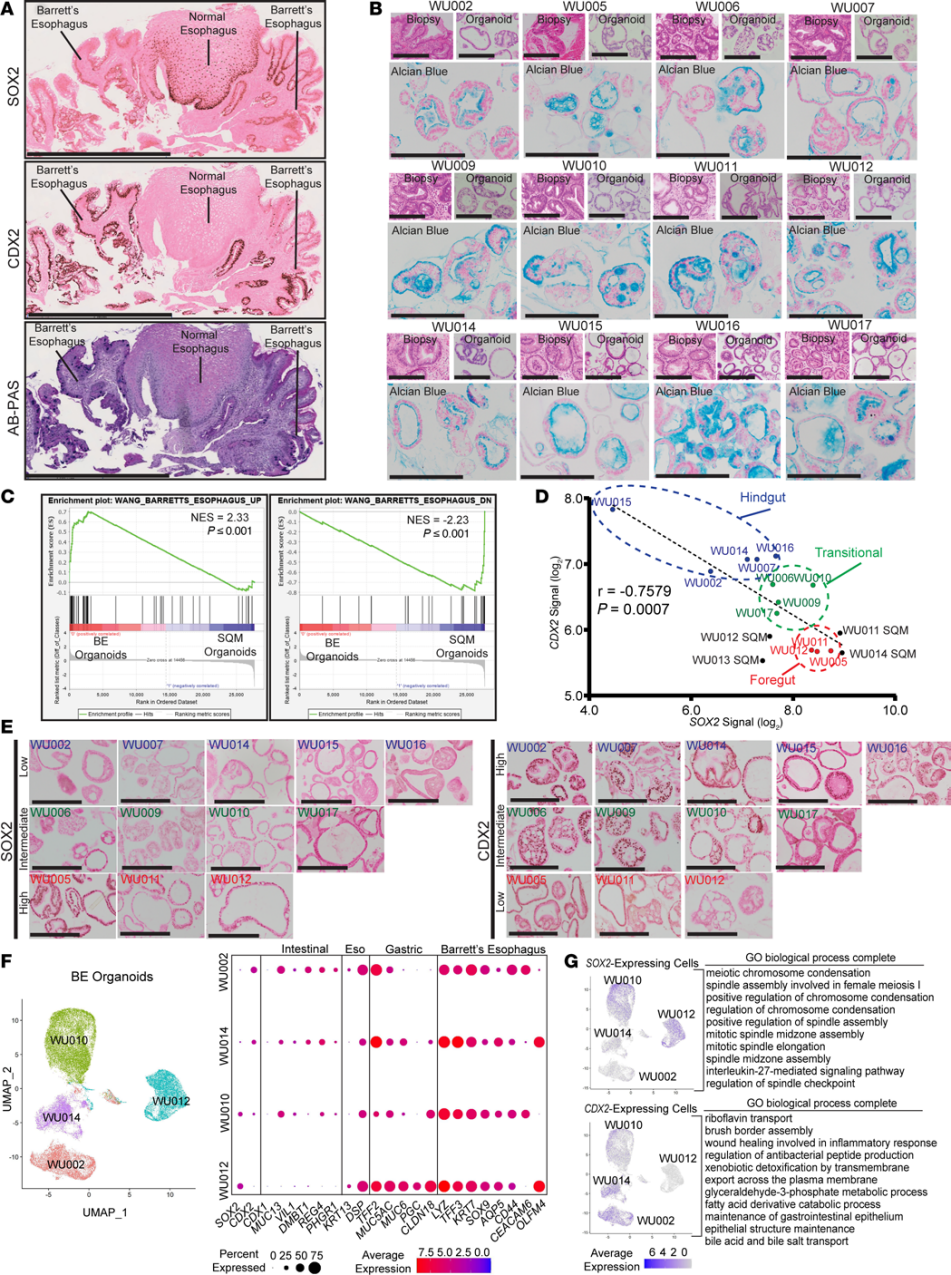
Barrett’s esophagus heterogeneity correlates with SOX2 abundance. (**A**) Human BE samples and normal esophagus stained for SOX2, CDX2, and Alcian blue-periodic acid-Schiff (AB-PAS). Scale bars: 1 mm. (**B**) Patient-derived BE organoids and paired biopsies stained with H&E; organoids also stained with Alcian blue to highlight mucus cells. Scale bars: 100 μm. (**C**) Gene set enrichment analysis showing enrichment of “WANG-BARRETTS_ESOPHAGUS_UP” and “WANG_BARRETTS_ESOPHAGUS_DN” gene sets for esophageal squamous (SQM) organoids versus BE organoids. Normalized enrichment scores and *P* values shown. (**D**) *SOX2* and *CDX2* expression in 12 BE and 4 SQM organoids; Pearson’s correlation coefficient (*r*) and *P* value shown. Groupings: hindgut (blue), transitional (green), and foregut (red). (**E**) BE organoids stained for SOX2 (left) and CDX2 (right), categorized as high, intermediate, and low expression. Scale bars: 100 μm. (**F**) scRNA-Seq of BE organoid lines WU002, WU014, WU010, and WU012. Uniform manifold approximation and projection (UMAP) shows total cells; dot plots display average expression and percentage of expressing cells for *SOX2*, *CDX2*, and lineage markers (intestinal, esophageal, gastric, BE). (**G**) UMAPs of *SOX2-* and *CDX2-*expressing cells among organoid lines; GO biological processes for differentially expressed genes in each population listed.

In samples from patients with BE, we observed that there was overall decreased expression of the foregut epithelium-promoting transcription factor SOX2 and increased ectopic expression of the intestinalizing transcription factor CDX2 (Figure 1A) (30). To analyze in more detail the patterns of transcription factor expression among the heterogeneous cells that compose BE in different patients, we generated a database of BE organoids based on previously published protocols (58, 59) from deidentified patient specimens (Figure 1B and Supplemental Table 1; supplemental material available online with this article; https://doi.org/10.1172/JCI190374DS1). We also successfully established 4 normal esophageal squamous organoid lines (WU011 SQM, WU012 SQM, WU013 SQM, and WU014 SQM) from biopsies of normal-appearing squamous tissue adjacent to BE lesions using a modified protocol for culturing keratinocytes in serum-free media (60, 61). These normal human squamous organoids ceased expanding after a few passages, so we confine our characterization of these organoids here to transcriptomic analysis.

We performed global transcriptomic analysis of the BE organoids relative to the 4 squamous organoids (NCBI’s Gene Expression Omnibus [GEO] GSE297800). Gene set enrichment analysis (GSEA) (62, 63) using gene sets from previously published transcriptomic analyses of genes differentially expressed in vivo in BE lesions versus normal esophageal tissues (64) showed that gene expression patterns of our BE organoids correlated well with those of in vivo BE lesions (Figure 1C). The global transcriptional analysis revealed substantial patient-to-patient BE organoid heterogeneity in overall expression of *CDX2* and *SOX2* with inverse correlation in expression such that *CDX2* high-expressors expressed low *SOX2* and vice versa (Figure 1D). We categorized organoids as “hindgut,” “transitional,” and “foregut” based on the balance of *SOX2* and *CDX2* expression. *SOX2* expression had relative negative correlative relationships with intestinal genes (*LYZ*, *TFF3*, and *OLFM4*) and positive correlative relationships with esophageal genes (*KRT13*, *DSG3*, and *TP63*) (Supplemental Figure 1, A and B). IHC staining of our BE organoids for protein expression of SOX2 and CDX2 corroborated the transcriptomic findings (Figure 1E).

To further delineate the functions of SOX2 and CDX2 in BE, we performed single-cell RNA-Seq (scRNA-Seq) on a subset of hindgut (WU002 and WU014), transitional (WU010), and foregut (WU012) BE organoids (Figure 1, F and G; GEO GSE298632). The foregut BE organoids were obtained from biopsies taken from BE lesions that were grossly visible to the endoscopist yet, on subsequent histopathological analysis, were shown to lack goblet cells (a requirement for the pathological diagnosis of BE in the United States; ref. 65) or had only one small focus of intestinal metaplasia in otherwise gastric columnar epithelium (Supplemental Table 1). Thus, the gross and histological features correlated with the organoid transcriptional phenotype (both bulk and single-cell), characterized by minimal intestinal (i.e., hindgut) differentiation (Figure 1F and Supplemental Table 1). Organoids with more hindgut characteristics (WU002 and WU014) showed more *CDX2*-expressing cells, whereas the foregut organoid line (WU012) showed more *SOX2*-expressing cells, and the transitional line (WU010) had mixed *SOX2* and *CDX2* fractions (Figure 1, F and G). Furthermore, WU002 and WU014 hindgut organoids expressed the most intestine-specific genes, the WU012 foregut organoid line expressed more esophageal and gastric genes, and all BE organoid lines robustly expressed many established gene markers of BE (32, 66–68) (Figure 1F). We next identified *SOX2*- and *CDX2*-expressing cells in the scRNA-Seq analysis and focused on the genes whose expression was coenriched with either *SOX2*-expressing or *CDX2*-expressing cell populations. *SOX2*-expressing cells were enriched for transcripts governing cell division and cell cycle regulatory functions based on Gene Ontology (GO) terms (69, 70) (Figure 1G). The transcripts preferentially expressed in *CDX2* cells were enriched for intestinal epithelial functions including nutrient transport, brush border assembly, and maintenance of gastrointestinal epithelium (Figure 1G).

The results indicate that, while *SOX2* expression may decrease in cells with more hindgut characteristics (and *CDX2* expression), *SOX2*-expressing cells can still be maintained. Moreover, these *SOX2*-expressing cells may be performing similar functions in all metaplastic lesions. SOX2 has a well-characterized, prooncogenic effect in esophageal squamous cell carcinomas (38, 71), but there is a lack of in vivo models elucidating the role of SOX2 in the adult foregut epithelium and its possible role in metaplasia of that epithelium. In addition, effects of *Sox2* loss have been assessed on the developing foregut endoderm (34, 35), but early death postnatally of mice with decreased *Sox2* expression has limited the ability to characterize the effects of *Sox2* loss on adult foregut squamous epithelium without more specific conditional deletion of the gene.

To this end, we developed an inducible model of *Sox2* loss in the murine foregut squamous epithelium, *Krt5^CreER/+^ Sox2^fl/fl^ ROSA26^LSLtdTomato/+^* mice (referred to as *Sox2*^Δ/Δ^ after induction). These mice express tdTomato upon Cre-mediated recombination of the *lox*-STOP-*lox* (*LSL*) cassette in the ubiquitously expressed *ROSA26* locus (72). In other words, tdTomato expression serves as a proxy to trace the *Krt5* lineage squamous cells that have lost *Sox2* expression after treatment with tamoxifen. Of note, the foregut of rodents differs from humans in that the proximal-most portion of the rodent stomach, the forestomach, is lined by squamous epithelium that histologically phenocopies that of the esophagus (73–75).

**Figure 2:**
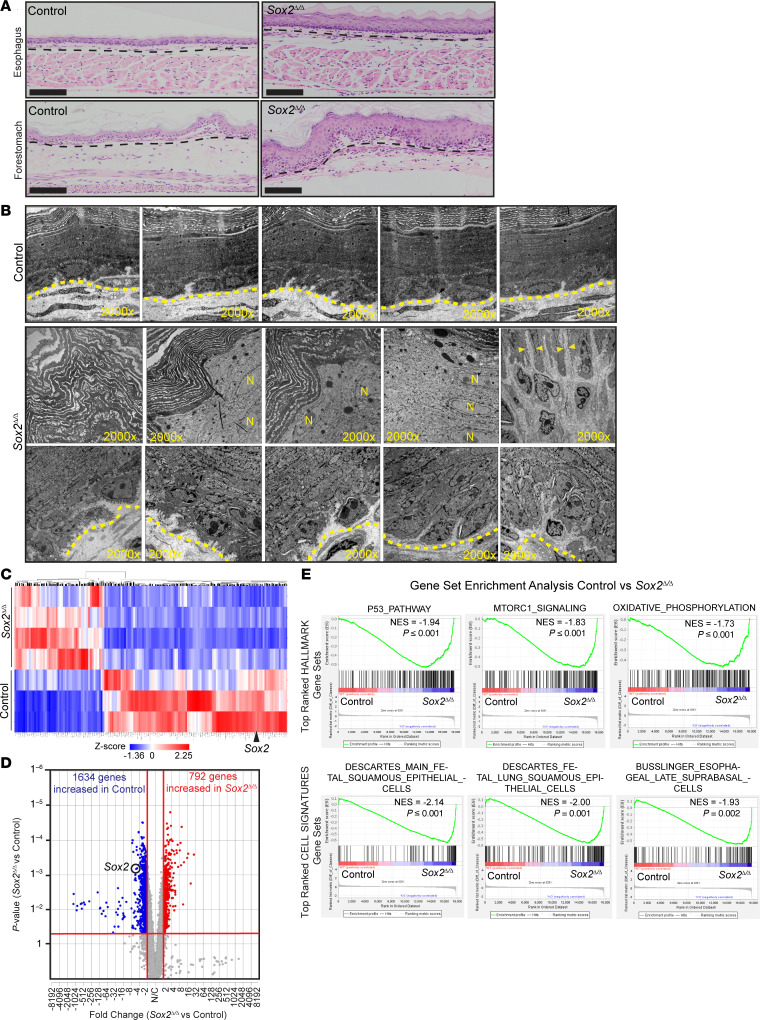
Loss of *Sox2* in the foregut squamous epithelium induces histological and transcriptional changes. (**A**) H&E staining of *Sox2*^Δ/Δ^ mice shows thickened esophagus and forestomach versus WT control. Scale bars: 100 μm. Images are representative of at least 3 independent experiments. (**B**) TEM of *Sox2*^Δ/Δ^ forestomach showing expanded basal cells, more nucleated surface cells (N = nucleus), enlarged cell-cell junctions (arrowheads), and disorganized keratin layers compared with control; basement membrane (dotted line); ×2,000 magnification. Presented TEMs are differing forestomach regions from the same *Sox2*^Δ/Δ^ or control animal. (**C**) Heatmap of differentially expressed genes in 4 *Sox2*^Δ/Δ^ versus 3 control forestomachs. Upregulated and downregulated genes shown in red and blue, respectively. *Sox2* labeled. (**D**) Volcano plot of differentially expressed genes from **C**. *Sox2* highlighted. (**E**) Gene set enrichment analysis of **C** with gene sets, normalized enrichment scores, and *P* values indicated.

We assessed the histological appearance of both the esophagus and forestomach foregut squamous tissues of control (littermates that lack the *Krt5^CreER/+^* allele) and *Sox2*^Δ/Δ^ mice 6 weeks after tamoxifen treatment (7 consecutive daily 1 mg/20 g mouse body weight i.p. injections; refs. 76, 77) to induce Cre recombinase and *Sox2* deletion (Figure 2A). Consistently, we saw thickening of the squamous epithelium in the esophagi and forestomachs of *Sox2*^Δ/Δ^ mice. We chose to primarily focus on the forestomach for the remaining experiments because it is in direct continuation with the glandular stomach, mimicking the human gastroesophageal junction; it is a larger and technically more easily assessable tissue; and it is histologically similar to the murine esophagus, as discussed above. Moreover, given that mice do not reflux into their esophagi, the squamocolumnar junction between the glandular stomach and forestomach may better model BE in humans where acid reflux is part of the pathogenesis. Biological differences in the murine forestomach and esophagus have been noted in prior studies (38), and our studies also reveal variations that we will highlight when applicable. Transmission electron microscopy showed that, in contrast to the thin squamous epithelium with prominent nucleated basal cell layer seen in control animals, the *Sox2*^Δ/Δ^ animals displayed markedly thickened forestomachs with expanded basal layer, increased intracellular spacing, and increased nucleated cells adjacent to the keratinized superficial layer (Figure 2B and Supplemental Figure 2). In addition, the *Sox2*^Δ/Δ^ animals had prominent autophagosomal structures and large dark keratin inclusions seldom seen in control forestomachs (Supplemental Figure 2). Bulk microarray transcriptomic analysis of the forestomachs from 4 *Sox2*^Δ/Δ^ and 3 control mice (GEO GSE297858) confirmed that *Sox2* was among the top transcripts altered and correlated with decreased or increased expression of a large cohort of other genes (Figure 2, C and D).

GSEA (62, 63) using the Hallmarks gene sets, which focus on general metabolic, inflammatory, and cell signaling functions, highlighted specific gene expression patterns with statistically significant normalized enrichment scores. *Sox2*^Δ/Δ^ forestomachs showed increased expression of genes related to p53 signaling and metabolism, such as those involved in the cellular energetics hub, mTORC1, and mitochondrial oxidative phosphorylation (Figure 2E and Supplemental Table 2). In addition, we used a collection of gene sets known as “Cell Signatures” to discover that *Sox2*^Δ/Δ^ forestomachs were enriched in gene sets related to fetal squamous epithelium and the progenitor suprabasal layer of squamous epithelium, consistent with SOX2 being required for full development and differentiation of squamous epithelium (Figure 2E and Supplemental Table 2).

**Figure 3:**
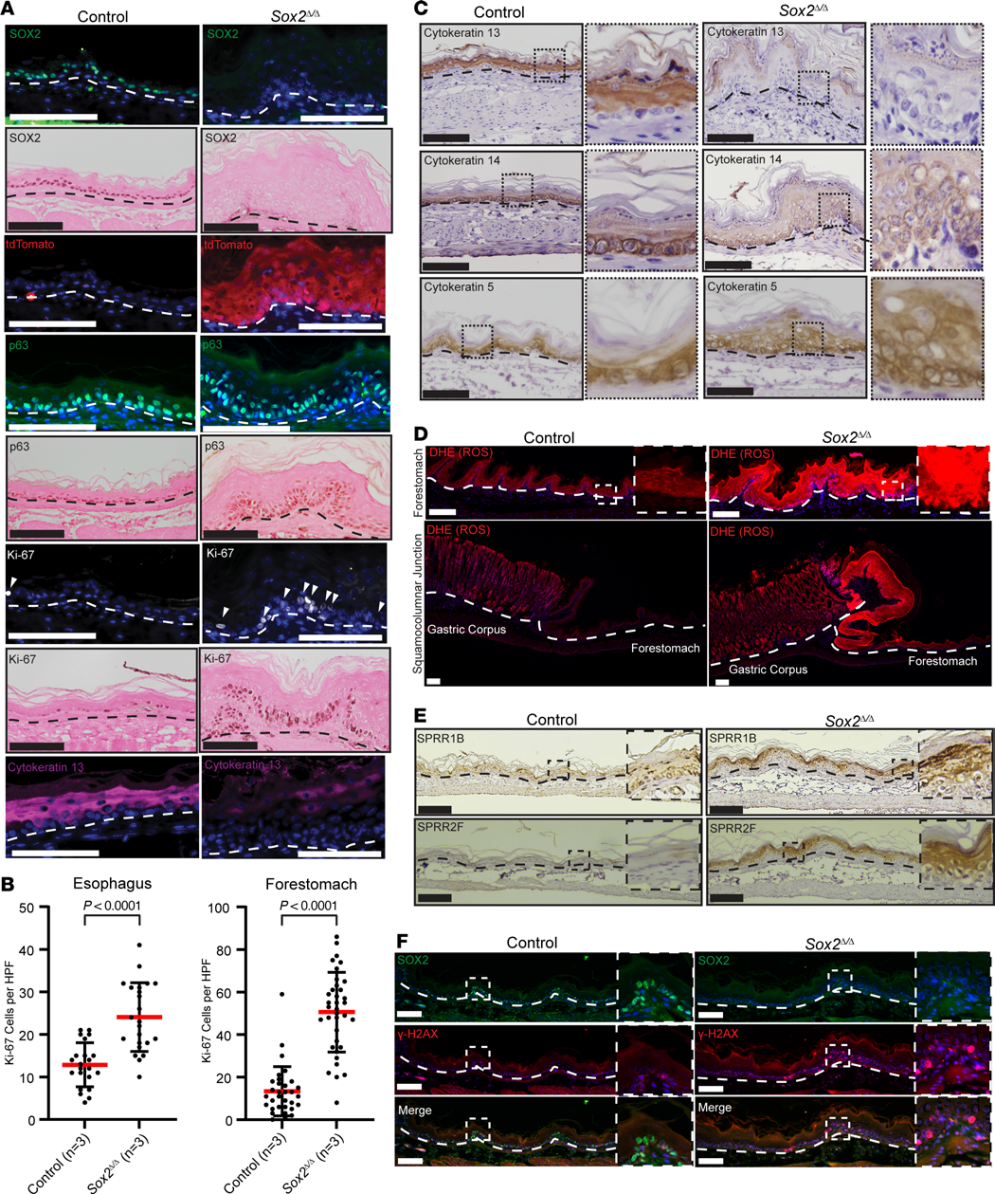
Loss of *Sox2* in the foregut squamous epithelium results in increased proliferation and decreased maturation. (**A**) Immunostaining of WT control and *Sox2*^Δ/Δ^ forestomachs showing SOX2 (green or brown nuclear), lineage-traced cells (tdTomato, red), p63 (green or brown nuclear), Ki-67 (white and brown nuclear, arrowheads), and cytokeratin 13 (purple). (**B**) Quantification of Ki-67^+^ cells in esophagi and forestomachs, 3 mice per group. Mean (red bar) ± SD (black bars). Two-tailed unpaired Student’s *t* test; *P* values indicated. (**C**) Immunostaining shows loss of surface cytokeratin 13 and expansion of basal cytokeratin 14 and basal cytokeratin 5 in *Sox2*^Δ*/*Δ^ forestomachs. (**D**) DHE staining detects ROS (red) in forestomach and gastric corpus of control and *Sox2*^Δ/Δ^ mice; basement membrane (dashed white line); insets show magnified forestomach regions. (**E**) SPRR1B and SPRR2F expression increased in *Sox2*^Δ/Δ^ forestomachs; insets show selected regions. (**F**) Immunofluorescence reveals SOX2 (green nuclear) loss and increased γ-H2AX (red nuclear) in *Sox2*^Δ/Δ^ forestomachs; insets highlight affected regions. All scale bars: 100 μm. Images are representative of at least 3 independent experiments.

We next decided to confirm the aberrant differentiation caused by loss of *Sox2* with targeted IHC analyses. First, we confirmed *Sox2* was efficiently deleted and correlated with tdTomato expression (Figure 3A). Next, we noted that a transcription factor typically expressed in the basal layers of squamous epithelium and required for squamous epithelium development, p63 (78), was more broadly expressed in both basal and intermediate layers in *Sox2*^Δ/Δ^ forestomachs (Figure 3A). Loss of *Sox2* alone did not lead to expression of the intestinal transcription factor, CDX2, as assessed by transcriptomic analysis and IHC (data not shown). The expanded p63^+^ basal cells in the forestomachs of *Sox2*^Δ/Δ^ mice expressed Ki-67, indicating an increase of basal cells in the cell cycle (Figure 3, A and B). Squamous epithelium maturation is characterized by changes in cytokeratin expression: KRT13 marks differentiated superficial cells and KRT5/14 are expressed in more immature basal layers (79). *Sox2*^Δ/Δ^ forestomachs showed decreased KRT13 and expanded KRT5/14, consistent with increased progenitors and impaired maturation (Figure 3, A and C). Of note, the phenotype of *Sox2* deletion in the esophagus was similar histologically to the pattern in the forestomach (Figure 2A); however, the increase in proliferation in mutants was statistically significant but less pronounced (Figure 3B). Unlike forestomach, KRT13 expression was unchanged in mutant esophagi (Supplemental Figure 3).

Given the increased p53 signaling and abundance of autophagosomes seen on transmission electron microscopy in *Sox2*^Δ/Δ^ forestomachs, we next examined elements of epithelial damage response pathways. We found a significant increase in markers of ROS, as evidenced by dihydroethidium staining, in the forestomachs of *Sox2*^Δ/Δ^ animals (Figure 3D). Small proline-rich proteins are a family of proteins that function in the squamous epithelium to protect against free radicals, prevent DNA damage, and counter p53 activation (80). In *Sox2*^Δ/Δ^ forestomachs, we saw a marked increase in SPRR1B and SPRR2F protein expression (Figure 3E), and *Sprr1b* and *Sprr2f* were also among the top transcripts increased with *Sox2* loss. Finally, consistent with increased p53 pathway expression and increased ROS and SPRR expression, we found *Sox2*^Δ/Δ^ forestomachs exhibited increased expression of the DNA damage repair marker, γ-H2AX (81), which, as expected, was not expressed in control forestomachs (Figure 3F). Thus, overall, histologically and transcriptionally, *Sox2*^Δ/Δ^ forestomachs displayed baseline induction of ROS along with increased markers of DNA damage and repair in the setting of increased proliferation and decreased maturation.

In a complementary approach, we generated another inducible model of *Sox2* loss in the murine foregut squamous epithelium using a different squamous epithelial driver: *Krt14^CreER/+^ Sox2^fl/fl^ ROSA26^LSLtdTomato/+^* mice. The cytokeratin 14 promoter has been used to perturb gene expression in the basal layer of multiple squamous epithelia (82). Upon induction of *Sox2* loss in these mice, we were not able to detect statistically significant or consistent changes in squamous epithelial phenotype in the esophagi or forestomachs (Supplemental Figure 4A), although the recombination rate in these animals with the dosing schemes we tried resulted in only rare focal *Sox2* loss and tdTomato induction (Supplemental Figure 4, B and C), likely due to failure to induce Cre efficiently. Alternatively, there may be a difference in expression between cytokeratin 14 and cytokeratin 5 promoters (83, 84). In any case, we did not continue to characterize the *Krt14^CreER/+^ Sox2*^Δ/Δ^ *ROSA26^tdTomato/+^* mice.

**Figure 4:**
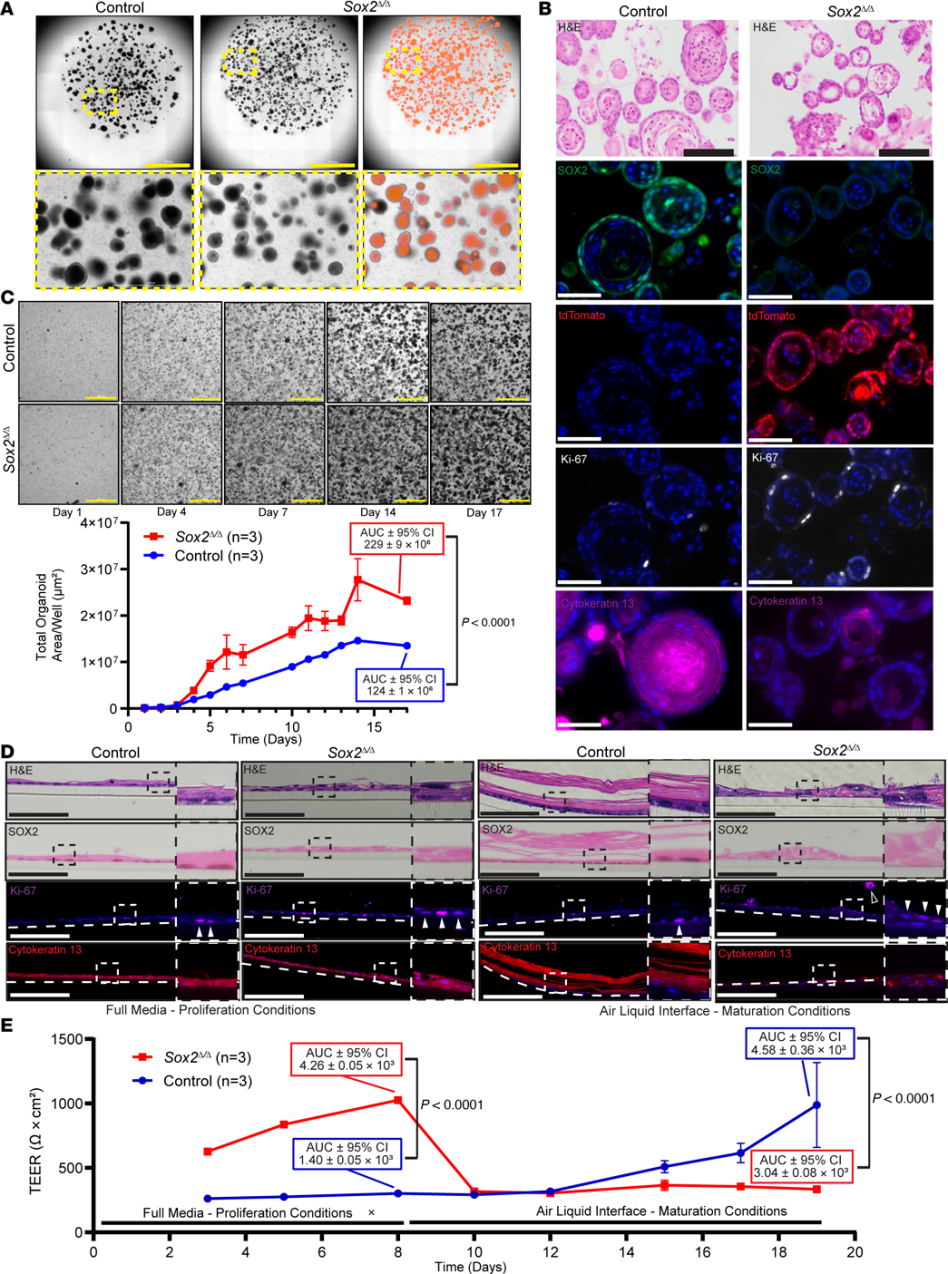
*Sox2*^Δ/Δ^ squamous organoids have increased proliferation and decreased maturation. (**A**) Bright-field images of forestomach organoids from WT control and *Sox2*^Δ/Δ^ mice. Right: tdTomato fluorescence indicates Cre activity and *Sox2* deletion. Bottom: higher magnification of boxed regions. Scale bars: 3,000 μm. (**B**) Immunostaining of *Sox2*^Δ/Δ^ squamous organoids shows SOX2 loss (green nuclear), tdTomato expression (red cytoplastic), increased Ki-67 (white nuclear), and reduced cytokeratin 13 (purple cytoplasmic). All scale bars: 100 μm. (**C**) Proliferation assay of squamous organoids tracked over 17 days; bright-field images shown. Scale bars: 1,000 μm. Below: total organoid area (mean ± SD, 3 wells/condition); AUC and 95% CI at day 17 shown. Two-tailed unpaired Student’s *t* test; *P* value indicated. (**D**) Transwell culture of squamous organoids under full media proliferation (left) and air-liquid interface (ALI) maturation conditions (right). H&E, SOX2 (brown nuclear), Ki-67 (purple nuclear, arrowheads), and cytokeratin 13 (red cytoplasmic) staining. Open arrowhead showing shed cell under ALI maturation conditions. Insets: higher magnification of boxed areas. Scale bars: 100 μm. (**E**) Transepithelial electric resistance (TEER) measurements (mean ± SD, 3 wells/condition) after 8 days in full media and 11 additional days under ALI conditions. AUC and 95% CI for full media and ALI conditions shown. Two-tailed unpaired Student’s *t* test; *P* values indicated. Images are representative of at least 3 independent experiments.

To more directly quantitate the effects of *Sox2* loss specifically and cell-intrinsically on epithelial proliferation and maturation, we generated organoids from *Sox2*^Δ/Δ^ and control animals using both forestomach and esophageal tissues (60, 61) (Figure 4, A and B, and Supplemental Figure 5A). When grown in 3D culture (i.e., in Matrigel droplets), the *Sox2*^Δ/Δ^ squamous forestomach organoids lacked the central layer of keratinization lining the organoid lumen (and paralleling the superficial-most layer in vivo) found in control organoids (Figure 4, A and B). As was observed in vivo, these *Sox2*^Δ/Δ^ organoids retained SOX2 loss, expressed the tdTomato lineage trace, showed increased Ki-67 staining, and had decreased cytokeratin 13 (Figure 4, A and B, and Supplemental Figure 5B). Similar to our in vivo findings above, *Sox2*^Δ/Δ^ esophageal squamous organoids had less increase in Ki-67 (non–statistically significant trend toward increase), and cytokeratin 13 expression was unchanged versus control organoids (Supplemental Figure 5, A and B). We quantified growth dynamics of *Sox2*^Δ/Δ^ and control squamous organoids by extracting and digesting the 3D organoids to single cells, plating 23,000 cells per well, and imaging continuously over 17 days (Figure 4C). *Sox2*^Δ/Δ^ organoids grew more robustly than the control in terms of the overall fraction of each well occupied by organoids (Figure 4C). We noted a seeming paradox in that individual *Sox2*^Δ/Δ^ organoids were smaller on average than the WT organoids, as measured by total organoid area (i.e., the largest cross-sectional area of each organoid; Supplemental Figure 5C). However, because WT organoids differentiate more completely than mutants, much of the total cross-sectional area was occupied by acellular keratin layers in the organoid lumen. When we instead measured only the cellular organoid area (i.e., the cross-sectional area of each organoid occupied by cells, excluding the lumen), we again saw that *Sox2* loss caused increased cell growth (Supplemental Figure 5C). To further detail proliferation and maturation, we transitioned the organoids to a 2D Transwell growth system (85–87). In submerged medium conditions that stimulate proliferation as cells are immersed in growth-promoting factors, we found that the *Sox2*^Δ/Δ^ organoids had increased numbers of Ki-67^+^ cycling cells with increased stratification of cells (Figure 4D and Supplemental Figure 5D). To assess effects of loss of *Sox2* on barrier function of the epithelium, we assessed transepithelial electrical resistance (TEER) (88) as a marker for epithelial integrity and found it was increased in *Sox2*^Δ/Δ^ organoids (Figure 4E). When organoids were transferred to culture conditions with the apices of cells exposed to air (i.e., in air-liquid interface), which simulates in vivo maturation conditions, we found, in contrast, a dramatic decrease in the TEER of *Sox2*^Δ/Δ^ organoids versus controls (Figure 4E). Of note, while proliferation for both control and *Sox2*^Δ/Δ^ organoids decreased in these maturation conditions, the *Sox2*^Δ/Δ^ organoids still maintained significantly increased proliferation compared with control organoids (Supplemental Figure 5D). The decrease in maturation (and expression of KRT13) seen with loss of *Sox2* caused substantial defects in epithelial integrity during air-liquid interface conditions, whereas control organoids showed increased maturation, thickness, and TEER (Figure 4, D and E). Thus, in vitro results confirmed that loss of *Sox2* led to increased proliferation and decreased maturation of the squamous epithelium.

**Figure 5:**
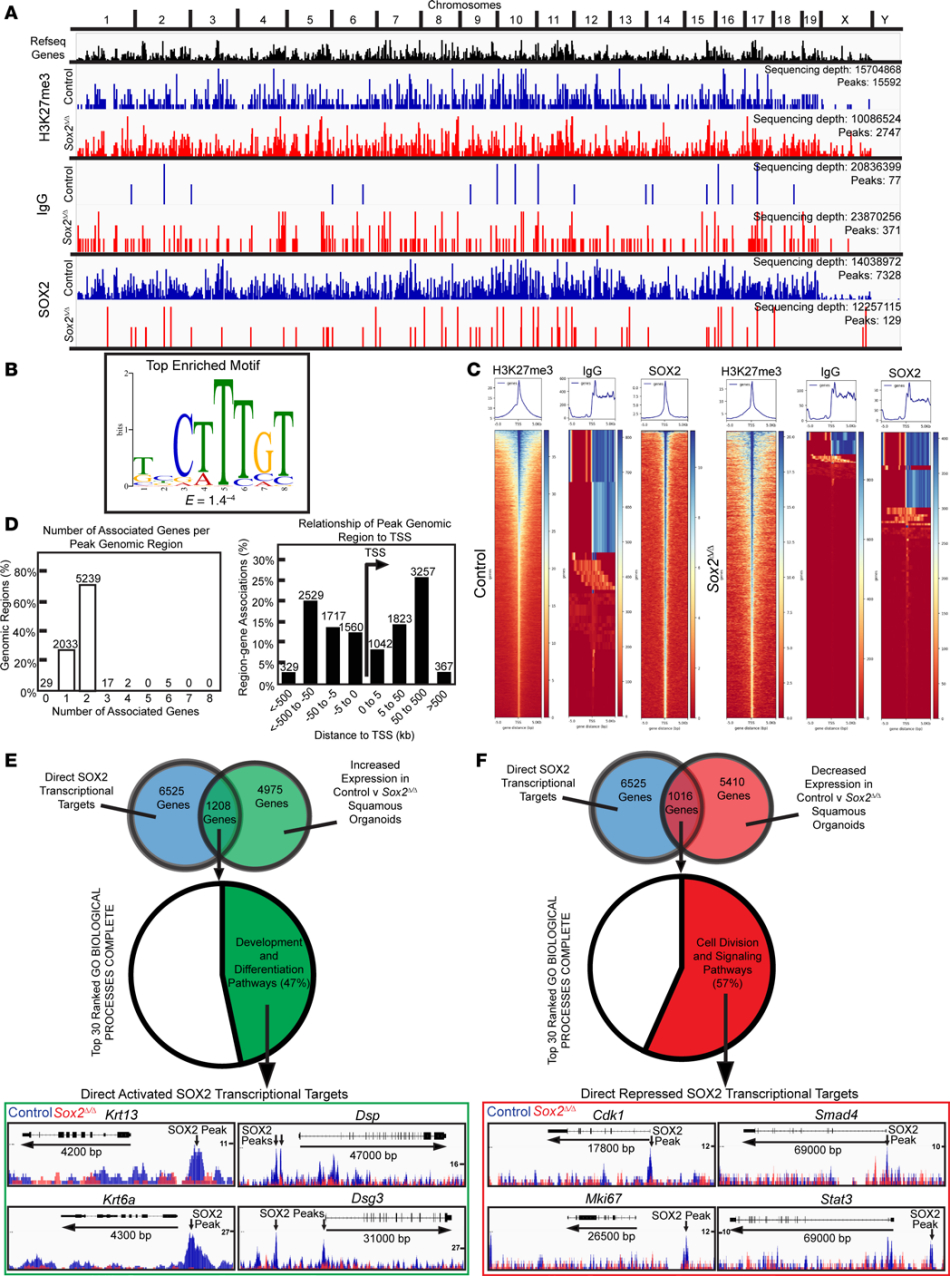
Direct transcriptional targets of SOX2 regulate proliferation and maturation. (**A**) Integrated genome browser view of CUT&RUN data for WT control (blue) and *Sox2*^Δ/Δ^ (red) squamous organoids probed using H3K27me3, IgG, and SOX2 antibodies. Sequencing depth and MACS2 peak calls displayed. (**B**) Top enriched motif identified from SOX2 CUT&RUN using MEME-ChIP and STREME with *E* value indicated. (**C**) Heatmaps and profile plots of CUT&RUN peaks –5 kb to +5 kb from transcription start sites for all genes in control and *Sox2*^Δ/Δ^ organoids. (**D**) Left: GREAT analysis showing number of genes associated with each SOX2 peak (total peaks labeled). Right: peak distribution relative to transcription start site. (**E** and **F**) Top: Venn diagrams showing overlap between SOX2-bound genes and those with altered expression in *Sox2*^Δ/Δ^ versus control organoids. Middle: pie charts of GO Biological Process terms among SOX2-bound genes with increased or decreased expression. Bottom: representative genes from GO categories (Development/Differentiation or Cell Division/Signaling) with gene structures and SOX2 binding peaks in control (blue) and *Sox2*^Δ/Δ^ (red) organoids.

Since SOX2 is a transcription factor, the phenotype we observed in *Sox2*^Δ/Δ^ squamous epithelium was likely due to changes in expression of its transcriptional targets. Given that there are limited studies that have elucidated the direct transcriptional targets of SOX2 in the adult foregut squamous epithelium, we sought to define the SOX2 transcriptional network using CUT&RUN (89–91). Control and *Sox2*^Δ/Δ^ forestomach squamous organoids were used for the CUT&RUN experiments with appropriate positive (antibody against H3K27me3) and negative controls (IgG) (Figure 5A; GEO 297942). As expected, loss of *Sox2* led to dramatic loss of SOX2-bound peaks: 7,328 peaks for control squamous organoids and 129 peaks for *Sox2*^Δ/Δ^ (Figure 5A). Reassuringly for the specificity of our analysis, the top *cis*-regulatory DNA sequence enriched in the 7,328 SOX2 peaks was the canonical motif for SOX2 binding to genomic DNA (Figure 5B) (92, 93). Peak density heat mapping showed that the SOX2-binding peaks were enriched in genomic regions containing transcription start sites of genes (Figure 5C). We assessed the relationship of these SOX2 peaks with associated genes using the Genomic Regions Enrichment of Annotations Tool (GREAT) (94, 95) and found 6,525 associated genes (Figure 5D).

We next integrated our CUT&RUN analysis with gene expression data generated from *Sox2*^Δ/Δ^ and control forestomach squamous organoids (Figure 5, E and F; GEO GSE297930). There were 1,208 genes that SOX2 bound in organoids whose expression was also decreased when *Sox2* was lost (Figure 5E), indicating SOX2 was responsible for activating their expression. GO term analysis for these SOX2-activated genes showed enrichment for pathways involved in differentiation and development (Supplemental Table 3). Representative genes included *Krt13*, *Krt6a*, *Dsp*, and *Dsg3* with SOX2 binding peaks upstream of the transcription start site (Figure 5E). There were 1,016 SOX2 transcriptional targets with the converse pattern of SOX2 binding with increased expression in *Sox2*^Δ/Δ^ (i.e., genes SOX2 represses). These showed enrichment in GO terms involved in cell division and signaling pathways like TGF-β (Figure 5F and Supplemental Table 3). Representative genes included *Cdk1*, *Smad4*, *Mki67*, and *Stat3* (Figure 5F). These sets of direct transcriptionally activated and repressed genes offer further mechanistic support of the phenotype seen in the *Sox2*^Δ/Δ^ mice of increased proliferation and decreased maturation.

**Figure 6:**
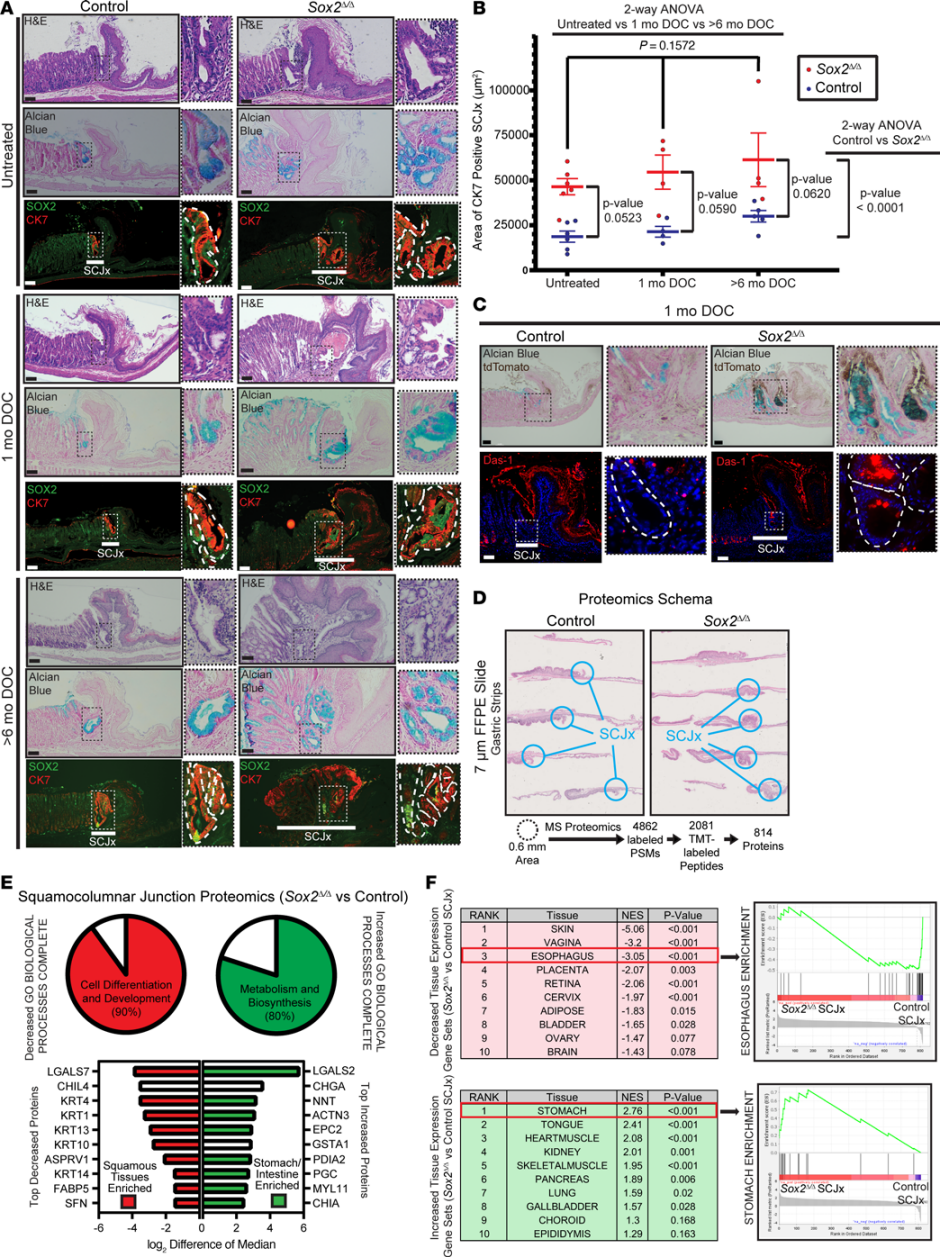
*Sox2* loss induces columnar expansion at the squamocolumnar junction. (**A**) H&E, Alcian blue, and immunofluorescence for SOX2 (green) and cytokeratin 7 (CK7; red) staining of WT control and *Sox2*^Δ*/*Δ^ forestomachs. Insets: magnified squamocolumnar junctions and glandular structures. Untreated (top), 1-month DOC-treated (middle), and more than 6-month DOC-treated (bottom). (**B**) Quantification of squamocolumnar junction areas (μm^2^, mean ± SEM) by CK7. Each point = average area from 3–7 squamocolumnar regions per mouse. Two-way ANOVA with Šidák’s post hoc test for genotype and treatment effects with *P* values indicated. (**C**) Alcian blue, tdTomato (brown), and Das-1 (red) staining in 1-month DOC-treated forestomachs. Insets highlight glandular changes at squamocolumnar junctions. Scale bars: 100 μm. Images are representative of at least 3 independent experiments. (**D**) Spatial proteomics from FFPE gastric strip tissue using on-site tissue protein labeling. Blue circles indicate 0.6 mm targeted regions. Mass spectrometry detected 4,862 peptide-spectrum matches (PSMs), 2,081 TMT-labeled peptides, and 814 proteins. (**E**) Comparative proteomics revealed 32 decreased and 782 increased proteins in *Sox2*^Δ/Δ^ versus control squamocolumnar junctions. Top: GO analysis showed decreased proteins were enriched for Cell Differentiation and Development; increased proteins were enriched for Metabolism and Biosynthesis. Bottom: top 10 enriched proteins per group shown, based on Human Protein Atlas expression in squamous tissues (red) or stomach/intestines (green). (**F**) Gene set enrichment analysis using Human Protein Atlas tissue-specific genes/proteins for 36 tissues. Top decreased: squamous tissues (e.g., skin, vagina, and esophagus; esophagus shown). Top increased: stomach shown. Top 10 enriched/depleted tissue sets shown with normalized enrichment scores and *P* values.

We did not observe an induction of glandular or intestinal differentiation in the squamous epithelium in the *Sox2*^Δ/Δ^ mice (e.g., we did not detect CDX2 expression). However, we did notice profound changes in the squamocolumnar junctions of mutant mice. Namely, where the forestomach meets the glandular stomach at the limiting ridge in the stomachs of the mice, there was expansion of Alcian blue–positive glands that were also marked by cytokeratin 7 (Figure 6A). KRT7 tends to label transitional glandular cells, submucosal gland duct epithelial cells, and BE (96–98). Others have reported that treatment of mice with the unconjugated bile acid deoxycholate (DOC; 0.3% in drinking water) can potentiate BE-like changes at the murine squamocolumnar junction (99–101). We assessed the short-term (1 month) and long-term (>6 months) effects of DOC treatment. Upon treatment with DOC at 0.3%, we saw increased morbidity and mortality of the *Sox2*^Δ/Δ^ mice (Supplemental Figure 6). As a result, we treated all mice using a dose deescalation schema in which mice were induced with DOC at 0.3% for 7 days and then maintained at 0.1% for the duration of treatment. We quantified the area of KRT7^+^ cells at the squamocolumnar junction in WT and mutant mice. In all cases, *Sox2*^Δ/Δ^ mice had statistically significant increases relative to equivalently treated controls; however, we did not see statistically significant effects of DOC treatment (Figure 6B).

Given the role of inflammation in synergizing with bile acids to potentiate BE-like changes in the murine squamocolumnar junction (99–101), we assessed inflammatory changes based on H&E staining for control and *Sox2*^Δ/Δ^ mice (Supplemental Figure 7A). We found no overt evidence of inflammation in the esophagi or forestomachs of *Sox2*^Δ/Δ^ mice; however, we did observe mixed inflammatory cell infiltrates at the squamocolumnar junction of these mice at both untreated and long-term DOC treated conditions (Supplemental Figure 7A). These inflammatory foci erupted into the gastric lumen with effacement of surface epithelial cells; on histology, the infiltrates appeared to be predominantly macrophages and neutrophils. To characterize the infiltrates, we immunostained for inflammatory markers including CD8 to mark cytotoxic T cells, STING to mark the cGAS-STING pathway (activated upon inflammation), F4/80 to mark macrophages, and Ly6G to mark neutrophils (Supplemental Figure 7, B and C). For both untreated and long-term DOC-treated *Sox2*^Δ/Δ^ mice, the infiltrates were populated by F4/80^+^ macrophages and Ly6G^+^ neutrophils. As above, we did not see a significant difference in these immune cell populations upon DOC treatment (Supplemental Figure 7C).

We next sought to determine the origin of the expanded glandular transitional cells by immunostaining for the tdTomato lineage trace from the *Krt5*-expressing squamous cells. Surprisingly, many of the expanded Alcian blue and KRT7 glandular cells coexpressed tdTomato, indicating squamous forestomach cells contributed to expansion of these glands and indicating some type of cell plasticity had occurred (Figure 6C and Supplemental Figure 8). Note, however, that many of the expanded glandular cells were also tdTomato-negative, indicating their expansion was not due to cell-autonomous loss of *Sox2* but a reaction to loss of *Sox2* in nearby cells. The expanded glands also expressed mAb Das-1, a well-established marker of BE metaplasia (102–104) (Figure 6C). We next performed “spot” (spatial) proteomic analysis (105) of WT and *Sox2*^Δ/Δ^ squamocolumnar junction glands from FFPE blocks of gastric strips (European Molecular Biology Laboratory [EMBL] European Bioinformatics Institute [EBI] PRoteomics IDEntifications database [PRIDE] PXD063992). We identified 814 proteins from 4 separate 0.6 mm tissue areas obtained from long-term DOC-treated control and *Sox2*^Δ/Δ^ gastric strips (Figure 6D). Of these, 32 proteins were decreased in *Sox2*^Δ/Δ^ squamocolumnar junctional glands versus the equivalent regions in WT mice. GO analysis revealed that almost all these proteins were related to squamous cell differentiation and development (Figure 6E and Supplemental Table 4). In the *Sox2*^Δ/Δ^ junctional glands, 782 proteins were increased, and these gastric and intestinal proteins were overwhelmingly categorizable within the biological process GO term “Metabolism and Biosynthesis” (Figure 6E and Supplemental Table 4). Using tissue-specific protein expression data from The Human Protein Atlas (106), we performed GSEA of the differentially expressed proteins in the squamocolumnar junctional glands and found a significant increase in proteins characterized as stomach-specific and decreased enrichment of proteins characteristic of organs lined by squamous epithelium (including esophagus) (Figure 6F and Supplemental Table 5). Thus, the expanded glands at the squamocolumnar junction were partially derived from squamous SOX2-expressing cells and were characterized by a metaplastic transition to glandular cells with increased gastric and decreased squamous phenotype.

## Discussion

**Figure 7:**
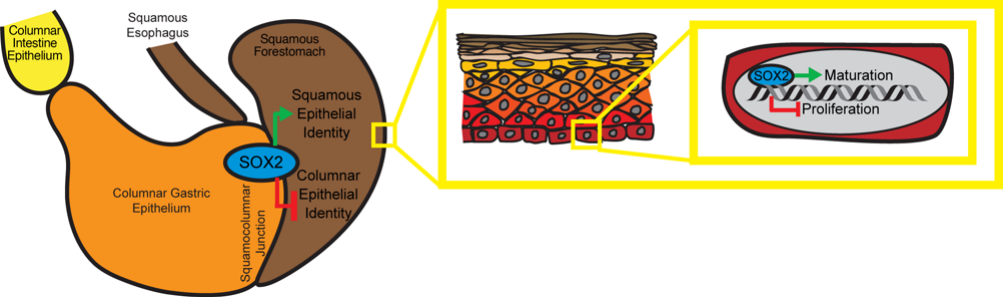
Diagram summarizing the role of SOX2 in murine foregut squamous epithelium. At the cellular level, SOX2 promotes squamous maturation and suppresses proliferation to maintain squamous epithelial homeostasis. SOX2 loss blocks maturation and induces a fetal-like proliferative state. At the tissue level, SOX2 maintains squamous identity and restricts columnar differentiation at the squamocolumnar junction. Its loss leads to junctional expansion, reduced squamous identity, and increased gastric/intestinal marker expression — mimicking features of human Barrett’s esophagus.

Our findings identified SOX2 as a master transcriptional regulator of foregut squamous epithelial identity (Figure 7). SOX2 is expressed throughout the foregut squamous epithelium and maintains homeostatic function by promoting squamous maturation genes and suppressing proliferation. At the squamocolumnar junction, SOX2 is critical for preserving the squamous-glandular boundary. Its loss leads to expansion of glands expressing metaplastic and gastrointestinal markers, positioning *Sox2*^Δ/Δ^ mice as a valuable mouse model for studying BE development. Unlike prior models, *Sox2*^Δ/Δ^ mice do not rely on engineered inflammation and develop BE-like glands more rapidly (100).

The squamocolumnar junction in the mouse stomach is more plastic and susceptible to metaplastic transformation. There is evidence that the transitional epithelium at the squamocolumnar junction can differentiate toward squamous and columnar lineages, expand upon injury, and may be a source of BE-like changes (97, 98, 107). Recent work has also shown that this squamocolumnar transitional epithelium is more prone to neoplastic transformation than other epithelial transitional zones, including the ovarian hilum and gastric antrum (108). The squamocolumnar junction transitional epithelium is maintained by numerous signaling pathways, including BMP4 (109, 110), osteopontin/CD44 (108), and regionally distinct *Fgf10*/*Fgfr2*-driven MAPK/ERK signaling (107). Our data showed that SOX2 not only promotes squamous maturation (Figure 5E), but also represses proliferation and signaling genes, particularly in the TGF-β/BMP pathway (Figure 5F). Loss of SOX2 leads to derepression of this signaling, suggesting that SOX2 normally restrains TGF-β/BMP signaling to allow/maintain normal squamous differentiation. In the absence of SOX2, increased TGF-β/BMP signaling may result in aberrant squamous differentiation and columnar expansion at the transition zone.

The transition zone’s cancer susceptibility and sensitivity to p53 loss (108, 111) align with our findings that SOX2 loss in the forestomach upregulates the p53 pathway (Figure 2E) and damage response markers (Figure 3, C–E). Inactivating *TP53* mutations are early events in progression of BE to esophageal adenocarcinoma (112, 113) and accumulate in the “normal” esophagus simply with age through clonal expansion (114). In addition, the presence of *TP53* mutations (which can be detected through abnormal IHC staining) can be used to identify patients with BE who are most at risk for esophageal adenocarcinoma progression (115). A SOX2/p53 regulatory network has been described in pancreatic cancer, where SOX2 compensates in p53-deficient pancreatic cancer cells to reduce stress and support proliferation (116). Similarly, combined SOX2 loss and aberrant p53 staining has been used to risk-stratify patients with BE at highest neoplastic risk (117). Our work provides mechanistic insight into this relationship.

SOX2 may have tumor-suppressive functions in the glandular stomach (118), given that its loss in the antrum derepresses intestinal/metaplastic genes and enhances Wnt-driven tumorigenesis (119). However, its function is context dependent. Namely, SOX2 can promote esophageal squamous cell carcinoma as it is commonly amplified in esophageal squamous cell carcinomas (71) and capable of driving cancer in overexpression mouse models (38, 120). SOX2 may act as a “rheostat” in the basal cells of the foregut squamous epithelium. Too much SOX2 expression (coupled with inflammation and other instigating signals) may drive oncogenesis. Too little (or loss of) SOX2 in these same cells may hinder mature squamous cell programming to unmask a proliferative intestinal gene expression program, leading to intestinal metaplasia and cancer progression. This ability of a transcription factor to have differing functions based on expression level and cellular specificity in the same tissue is not unique to SOX2: SOX9 has been observed to have diverse protumorigenic and antitumorigenic roles in the intestinal epithelium (121–123).

Most of the cell-intrinsic phenotypes in the *Sox2*^Δ/Δ^ mice are consistent with decreased maturation and increased proliferation in the face of increased ROS and DNA damage. However, *Sox2*^Δ/Δ^ squamous cells also showed increased autophagic structures in various stages of flux. This finding may correlate with recent studies indicating that increased autophagic activity marks the most proliferative and/or stem-like cells in the squamous epithelium (124).

The cell of origin for BE remains unclear, with several possible contributors (10). Our data support a heterogeneous origin for BE-like changes. In *Sox2*^Δ/Δ^ mice, expanded squamocolumnar glands expressing the ROSA26^tdTomato^ lineage tracing reporter (Figure 5C) — driven by the squamous basal layer *Krt5* promoter — indicate that some glandular cells arise from squamous progenitors. To our knowledge, no prior in vivo model has demonstrated the ability of the squamous epithelium to give rise to metaplastic columnar glands. Further work will need to define whether this phenomenon is occurring through transdifferentiation of mature squamous epithelial cells or through transcommitment of a basal layer squamous progenitor cell (125). Notably, many expanded cells were not tdTomato^+^, suggesting that SOX2 loss also alters neighboring WT cells, potentially of nonsquamous origin, such as gastric cardia (100, 126) or transitional junctional cells (97, 98). The local inflammatory changes at the squamocolumnar junction of the *Sox2*^Δ/Δ^ mice are similar to findings seen in the *L2-IL-1β* model (100), raising the possibility that SOX2 loss may promote junctional expansion (of both *Sox2*-null and WT cells) via inflammation.

Multiple cells of origin may underlie the phenotypic heterogeneity of BE lesions, which often contain both gastric and intestinal lineages (29, 66). We have established and characterized a BE organoid biobank that recapitulates this heterogeneity, revealing distinct subgroups based on an inverse expression pattern of SOX2 and CDX2. These data are consistent with other work regarding the role of these transcription factors in determining gastric versus intestinal differentiation in BE (127). scRNA-Seq showed *SOX2*-expressing cells consistently exhibit a cell division gene expression profile. We did not see evidence of a “keratinization” process in these *SOX2*-expressing BE cells, which likely indicates that these *SOX2*-expressing BE cells differ from SOX2-expressing cells from the squamous epithelium. These *SOX2*-expressing BE cells may arise from transdifferentiation or transcommitment of a squamous cell (125) or from glandular cells with lineage plasticity that reexpress SOX2 after BE development.

It is unlikely that SOX2 is the only transcription factor involved in BE initiation, as there is likely to be a stepwise or piecemeal transcriptional program with loss of esophageal traits and gain in gastrointestinal traits. Early embryonic loss of the squamous transcription factor p63 induces BE-like changes in the developing foregut (78, 98), and SOX2 interacts with p63 in squamous cells to regulate squamous-specific genes like *SLC2A1* (128, 129). Restoring SOX2 could theoretically reverse BE and halt progression to cancer, but transcription factors remain difficult drug targets (130). Ectopic overexpression of *Sox2* in mouse intestine induces a foregut-like phenotype and suppresses intestinal genes by reducing CDX2 binding to its targets (131), highlighting the ability of SOX2 to reprogram epithelial identity.

Beyond loss of transcription factors like SOX2, BE likely involves aberrant induction of gastric and intestinal transcription factors. GATA4, a gastric developmental transcription factor (132), represses squamous genes including *TP63* while promoting columnar identity (133). Interestingly, GATA4 has also been found to “balance” SOX2 expression at the squamocolumnar junction to pattern and lineage-specify the transitional (cytokeratin 7 expressing) epithelium toward a columnar epithelium (107). Conditional knockin of *Gata4* in the developing forestomach resulted in columnar-like cells with a glandular stomach expression pattern that showed paradoxically increased *Sox2* and decreased *Trp63* expression, and no expression of *Cdx2* (132). HNF4α, another gastric/intestinal transcription factor (134), promotes a columnar phenotype and can induce *Cdx2* expression via enhancer activation (52, 55). However, CDX2 alone is insufficient to fully intestinalize esophageal squamous cells (52, 53, 135), suggesting that SOX2 loss may be an early event during BE development that may precede aberrant expression of gastric/intestinal transcription factors. Not only does SOX2 specify direct squamous maturation, but its expression also inhibits proliferation signals and tumor suppressor mechanisms that have been shown to be required for subsequent CDX2-mediated intestinalization of esophageal squamous epithelium (54). Supporting this finding, combined p63 loss and CDX2 overexpression in graft models can induce BE-like changes (136). Further experiments are needed to dissect how combinations of losing normal esophageal factors and gaining intestinal factors drive intestinal metaplasia.

## Methods

See Supplemental Methods for full details.

### Sex as a biological variable.

We used human samples obtained from male and female patients and our study examined male and female animals; similar findings are reported for both sexes.

### Study approval.

Human BE FFPE tissue blocks were collected from the archives of Johns Hopkins School of Medicine Bayview Medical Campus Department of Pathology. The use of FFPE blocks was approved by the IRB of Johns Hopkins University School of Medicine (IRB ID 00262408). BE or normal adjacent esophageal squamous organoids were derived from deidentified tissue from patients with associated clinical details who were undergoing BE surveillance esophagogastroduodenoscopy for previously identified nondysplastic BE and provided informed consent through the Washington University School of Medicine Digestive Disease Research Core Center, with approval by the IRB of Washington University School of Medicine (IRB ID 201111078).

All experiments involving animals were performed according to protocols approved by the Washington University School of Medicine Animal Studies Committee and the IACUC of Baylor College of Medicine following federal guidelines.

### Statistics.

All Ki-67 proliferative quantifications were conducted by counting multiple high-powered fields from randomly selected regions of the esophagus or forestomach from at least 3 mice or at least 3 independent organoid wells per experimental condition. For quantification of organoid proliferation in 3D Matrigel growth conditions, Ki-67^+^ cells were divided by the total number of cells per organoid to generate a proportion of proliferative cells per organoid.

Statistical analyses were performed using GraphPad Prism 10. When comparing 2 conditions, a 2-tailed unpaired Student’s *t* test was used to quantify the likelihood of a true differences in means. For comparisons between multiple groups, 2-way ANOVA followed by post hoc Tukey’s test or Šidák’s multiple-comparison test was used to determine significance, as indicated. A *P* value of 0.05 or less was considered significant. Comparison of continuous organoid growth area and TEER was performed by AUC calculations; AUCs for TEER measurements under maturation and proliferation conditions were analyzed separately. Survival rates were analyzed using the Kaplan-Meier survival test. Data are generally expressed as mean ± SD except when statistical significance among multiple means was computed, in which case SEM is used. Samples were randomized, and measurements were blinded to prevent the introduction of experimental bias.

### Data availability.

The following microarray and sequencing data can be accessed in NCBI’s GEO: human BE organoid microarray and single-cell sequencing data (GSE297800 and GSE298632, respectively); mouse forestomach and organoid microarray data (GSE297858 and GSE297930, respectively); and mouse forestomach organoid CUT&RUN sequencing data (GSE297942). Mouse squamocolumnar junction spatial proteomics data can be accessed via EMBL-EBI PRIDE repository (PXD063992). The datasets are available from the corresponding authors upon request. Values for all data points in graphs are reported in the Supporting Data Values file.

## Author contributions

RUJ and JCM designed studies, performed experiments, analyzed data (including bioinformatics), provided funding, and wrote and edited the manuscript. YX, TML, and QKL designed studies, conducted experiments, acquired and analyzed data, and edited the manuscript; QKL also accrued and analyzed human tissue specimens. YZH conducted bioinformatics analyses and edited the manuscript. TMN performed experiments, acquired data, and edited the manuscript. BES and OMD performed experiments and acquired data. JSW identified patients with BE and obtained specimens from patients with PE. HZ designed studies. All authors provided manuscript feedback.

## Supplementary Material

Supplemental data

Supporting data values

## Version 1 06/30/2025

In-Press Preview

## Version 2 08/15/2025

Electronic publication
