# Supplementary material for: SOX2 regulates foregut squamous epithelial homeostasis and is lost during Barrett’s esophagus development
Source: J Clin Invest. 2025 Jun 19;135(16):e190374. doi: 10.1172/JCI190374 (PMC12352906; doi:10.1172/JCI190374)
Supplement: Supplemental data [file jci-135-190374-s018.pdf]

SUPPLEMENTAL METHODS

Human Barrett's Esophagus Biopsy Immunohistochemical Staining

Human Barrett's esophagus biopsy sample staining was performed by the Johns Hopkins Hospital Department of Surgical Pathology. FFPE blocks were cut into 4 µm sections, followed by de-paraffinization and rehydration prior to incubation with primary antibodies. For immunostaining of SOX2 and CDX2, a Ventana Discovery Ultra autostainer (Roche Diagnostics) was used. Briefly, antigen retrieval was performed using Ventana Ultra CC1 buffer (Roche Diagnostics) at 96ºC for 48 minutes. The primary antibody (**Supplemental Table 6**) was applied at 36ºC for one hour. The primary antibody was then detected using a HQ detection system (Roche Diagnostics), followed by visualization with Chromomap diaminobenzidine (DAB) IHC detection kit (Roche Diagnostics). Manual Alcian blue and Periodic Acid – Schiff (pH 2.5) staining was performed by the Johns Hopkins Hospital Department of Surgical Pathology.

Human Barrett's Esophagus Organoid Culture System

Barrett's esophagus or normal adjacent esophageal squamous organoids were derived from de-identified tissue from patients undergoing Barrett's esophagus endoscopic surveillance. In brief, Barrett's esophagus organoids were cultured with L-WRN-conditioned media (1) (2) supplemented with 10 nM gastrin (Sigma-Aldrich), 10 mM nicotinamide (Sigma-Aldrich), 500 nM A83-01 (Tocris), 10 µM SB202190 (Sigma-Aldrich), 200 ng/mL FGF10 (Peprotech), 10 µM Y-27632 (Sigma-Aldrich), and 10 µM Primocin (Invivogen) in 3D Matrigel (Corning) in 24-well plates (Greiner Bio-One). Esophageal

squamous organoids were cultured using keratinocyte-SFM (KSFM) media (3, 4) supplemented with 50 μg/mL Bovin pituitary extract (Gibco), 10 μM Y-27632 (Sigma-Aldrich), 1 ng/mL EGF (Gibco), and 0.6 mM calcium chloride (Sigma-Aldrich). Organoids were expanded and passaged every 7-10 days by mechanical dissociation from Matrigel, dispersed using TrypLE (Gibco) and manual pipetting, and transferred into new Matrigel and plated in 24-well plates.

Human Barrett's Esophagus Organoid Immunohistochemical Staining

Human Barrett's esophagus organoids were pelleted and fixed in 10% formalin at 4°C for one hour. Organoids were washed in PBS, moved to 70% ethanol, pelleted, mounted in 3% agar and embedded in paraffin. Blocks were cut into 7 μm sections. Immunohistochemical staining was performed on microtome sections after deparaffinization with Histoclear and rehydration followed by antigen retrieval in sodium citrate buffer (2.94 g sodium citrate, 500 uL Tween 20, pH 6.0) using a pressure cooker. Sections were blocked in 5% normal serum and left overnight with primary antibody (**Supplemental Table 6**). Sections were washed in PBS and incubated for one hour with biotinylated secondary antibody and then washed prior to mounting and visualization with Vectastain ABC-HRP DAB IHC detection kit (Vector Laboratories) and counterstaining with eosin Y. Alcian blue staining of organoids was performed using the Alcian blue (pH 2.5) staining kit (Vector Laboratories). Briefly, sections were deparaffinized as described above and hydrated in distilled water. Slides were then incubated in acetic acid solution and Alcian blue solution prior to washing and Nuclear fast red solution counterstaining.

Hematoxylin and eosin (H&E) staining was performed by the Washington University in St. Louis DDRCC-AITAC Morphology Core.

Human Barrett's Esophagus Organoid Transcriptomic Profiling

Human Barrett's esophagus organoids were pelleted, and RNA was extracted using the AllPrep DNA/RNA/Protein Mini Kit (Qiagen) per manufacturer's protocol. For microarray analyses, samples were processed and hybridized to Affymetrix Human Gene 2.0 ST per the manufacturer's instructions by the Washington University Genome Technology Access Core (GTAC). GeneChips were analyzed with Transcriptome Analysis Console (Applied Biosystems) analysis software to perform array QC, data normalization, and differential expression analyses. The human BE organoids microarray data is available from the GEO repository as GSE297800. Gene Set Enrichment Analysis (GSEA) (5, 6) was done using default settings with GMX files acquired from GSEA molecular signatures database (WANG_BARRETTS_ESOPHAGUS_UP and WANG_BARRETTS_ESOPHAGUS_DN)(7).

For human Barrett's esophagus organoid single-cell expression profiling, organoids were pelleted and digested to single cells using TrypLE (Gibco) digestion and manual pipetting dissociation, and then neutralized with Advanced DMEM/F12 (Gibco), 10% FBS (Gibco), 1X HEPES, 1X L-glutamine, 1X Pen/strep, 10 μM Y-27632 (Biogems), and 10 μM Primocin (InvivoGen). Cells were filtered using a 30 μm filter, counted, and volume adjusted before submission to the Washington University Genome Technology Access Core (GTAC). Processing of tissue for scRNA-seq was conducted by the Washington University Genome Technology Access Core using 10x Genomics platform

(10X Chromium Next GEM Single Cell 3' v3.1; 10x Genomics). The human BE organoids single-cell sequencing data is available from the GEO repository as GSE298632. Initial raw data were processed using the CellRanger 4.0 pipeline with human reference genome GRCh38. Clustering and differential expression analysis were conducted in Seurat/R (R Version 4.4.0-“Puppy Cup”; Seurat v5). Before cluster-based analysis, quality control of data was done to remove doublets and low-quality cells based on a predetermined threshold of mitochondrial markers and reads per cell. The following quality control parameters were used: percent.mt < 25, nFeature_RNA > 700, and nCount RNA < 75000. Each library was then globally scaled and normalized. Principle component analysis (PCA) was performed on the scaled, normalized data, and unbiased clustering analysis performed with uniform manifold approximation and projection (UMAP) with the following parameters: dims = 1:12, and resolution = 0.3. The datasets for *SOX2*- and *CDX2*-expressing cells are subsets based on certain cutoffs of gene expression of *SOX2* and *CDX2* followed by differential expression analysis. Gene Ontology biological analyses of statistical overrepresentation testing for GO biological processes complete was performed on these differentially expressed genes for the *SOX2*-expressing and *CDX2*-expressing cells using PANTHER (Protein ANalysis THrough Evolutionary Relationships) (8, 9).

Animals

Mice were kept in grouped housing and maintained in a specified-pathogen-free barrier facility under a 12-hour light cycle and fed regular chow. The mouse alleles B6N.129S6(Cg)-$Krt5^{tm1.1(cre/ERT2)Blh}$/J (Stain no: 029155, referred to as $Krt5^{CreER}$)(10),

Sox2$^{tm1.1Lan}$/J (Strain no: 013093, referred to as *Sox2*$^{fl/fl}$)(11), B6.Cg-*Gt(ROSA)26Sor*$^{tm9(CAG\text{-}tdTomato)Hze}$/J (Strain no: 007909, referred to as *ROSA26*^{LSLtdTomato})(12), and STOCK Tg(KRT14-cre/ERT)20Efu/J (Stain no: 005107, referred to as *Krt14*^{CreER})(13) were purchased from Jackson Laboratories. Experimental and control groups each contained at least three mice, with representatives from both genders. There were no differences in results observed between male and female mice. Littermate controls were housed together, when possible, to minimize differences in gastric microflora. To induce gene deletion but not gastric injury, "low-dose" tamoxifen (1 mg/20 g body weight; Toronto Research Chemicals) was injected intraperitoneally for 7 consecutive days (14). Tamoxifen was dispersed in 100% ethanol by sonication and then emulsified in sunflower oil (Sigma-Aldrich). Deoxycholate (Sigma-Aldrich) was administered in drinking water (15-17) DOC at 0.3% for 7 days and then treated at 0.1% for the duration of treatment.

Mouse Squamous Organoid Culture System

Mouse foregut squamous tissues from esophagi and forestomachs were dissected, manually disrupted, and enzymatically digested with collagenase IX (Sigma-Aldrich) and dispase (Gibco). Dissociated squamous cells were then plated in Matrigel (Corning) droplets and supplemented with keratinocyte-SFM (KSFM) media (3, 4) supplemented with 50 μg/mL Bovin pituitary extract (Gibco), 10 μM Y-27632 (Sigma-Aldrich), 1 ng/mL EGF (Gibco), and 0.6 mM calcium chloride (Sigma-Aldrich). Organoids were expanded and passaged every 7-10 days by mechanical dissociation from Matrigel,

dispersed using 0.05% Trypsin-EDTA (Thermo Fisher) and manual pipetting, and transferred into new Matrigel and plated in 24-well plates (Greiner Bio-One).

Mouse Tissue Immunohistochemical Staining

Mouse tissues were immediately excised and flushed with PBS and fixed overnight in 4% paraformaldehyde in PBS. Tissues were washed, embedded in 3% agar, and then underwent routine paraffin processing. For immunohistochemistry, tissue microtome 7 μm sections underwent standard deparaffinization serial Histoclear washes, rehydration with an ethanol series, quenching in a methanol solution containing 1.5% hydrogen peroxide, and antigen retrieval in sodium citrate buffer (2.94 g sodium citrate, 500 mL Tween 20, pH 6.0) using a pressure cooker. Sections were then blocked in 5% normal serum and left overnight with primary antibodies (**Supplemental Table 6**). After secondary antibody, color reaction was performed using Vectastain Elite ABC HRP Kit (Peroxidase, Standard) (Vector Laboratories) as detailed per the manufacturer's protocol. Slides were exposed using DAB Substrate Kit (Thermo Fisher) and mounted in Permount Mounting Medium (Fisher Chemical). Alcian blue staining was performed using the Alcian blue (pH 2.5) staining kit (Vector Laboratories). Briefly, sections were deparaffinized as described above and hydrated in distilled water. Slides were then incubated in acetic acid solution and Alcian blue solution prior to washing and Nuclear fast red solution counterstaining. Hematoxylin and eosin staining was performed by the Washington University in St. Louis DDRCC-AITAC Morphology Core or the Baylor College of Medicine Digestive Diseases Center Histology Component of the Tissue Analysis and Molecular Imaging (TAMI) Core.

Assessment of inflammation was performed using the Bond RX^{m} automated staining system with the “BOND Polymer Refine Detection” reagent kit (Leica). Slides were pretreated for staining with a 15 to 60-minute bake and dewax step followed by 20 minutes of antigen retrieval in citrate (ER1) buffer at 95°C. Tissues were stained with primary antibody (**Supplemental Table 6**) for one hour at room temperature followed by HRP-conjugated secondary antibody for 30 minutes at room temperature. Tissue-bound immunocomplexes were detected by incubating with the peroxidase substrates, DAB (8 minutes) or 3-amino-9-ethylcarbazole (AEC, 30 minutes), at room temperature. Finally, tissue sections were counterstained with hematoxylin.

For immunofluorescence, the following adjustments were made to the immunohistochemistry procedure; otherwise, all steps were identical. Sections were blocked in 1% BSA, 0.3% TritonX-100 in PBS for one hour, left overnight with primary antibodies at 4°C (**Supplemental Table 6**). Slides were incubated with Alexa Fluor fluorescent secondary antibodies (Thermo Fisher) for one hour. Slides were then washed in PBS and mounted using ProLong Gold antifade mountant with DAPI (Molecular Probes) and stored at 4°C.

To assess ROS production, fresh mouse tissue was embedded in OCT and sectioned using a frozen microtome (Leica). Tissue frozen sections were incubated in 10 μM Dihydro-Ethidium (DHE, Thermo Fisher) for 15 minutes at room temperature, washed in 1X PBS, and mounted using ProLong Gold antifade mountant with DAPI (Molecular Probes).

For transmission electron microscopy, stomach forestomach tissue was collected as described above, fixed overnight at 4°C in modified Karnovsky’s fixative or

glutaraldehyde, and sectioned into rings. Tissue rings were processed for EM by the Washington University in St. Louis Department of Pathology and Immunology Electron Microscopy Facility or the TAMI Core of the Baylor College of Medicine Digestive Diseases Center.

Mouse Squamous Organoid Immunohistochemical Staining

Mouse squamous organoids were pelleted and fixed in 4% paraformaldehyde in PBS at 4°C for one hour. Organoids were washed in PBS, moved to 70% ethanol, pelleted, mounted in 3% agar and embedded in paraffin. Blocks were cut into 7 μm sections. Hematoxylin and eosin staining was performed by the Washington University in St. Louis DDRCC-AITAC Morphology Core or the Baylor College of Medicine Digestive Diseases Center Histology Core. For immunofluorescence staining, sections underwent standard deparaffinization serial Histoclear washes, rehydration with an ethanol series, and antigen retrieval in sodium citrate buffer (2.94 g sodium citrate, 500 mL Tween 20, pH 6.0) using a pressure cooker. Sections were blocked in 1% BSA, 0.3% TritonX-100 in PBS for one hour, left overnight with primary antibodies at 4ºC (**Supplemental Table 6**). Slides were incubated with Alexa Fluor fluorescent secondary antibodies (Thermo Fisher) for one hour. Slides were then washed in PBS and mounted using ProLong Gold antifade mountant with DAPI (Molecular Probes) and stored at 4°C.

For transwell squamous organoid staining, squamous organoids grown in transwells were fixed with 10% formalin at 4°C for one hour. Transwell organoids were washed in PBS, moved to 70% ethanol, mounted in 3% agar and embedded in paraffin.

Blocks were cut into 7 μm sections and immunohistochemical staining including immunofluorescence was performed as described above.

Mouse Forestomach Tissue and Organoid Transcriptomic Profiling

Mouse forestomach tissues from four *Sox2*$^{\Delta/\Delta}$ mice and three wildtype control mice were harvested and flushed with PBS. Corpus and antrum were removed. The forestomach tissues were mechanically dissociated using a tissue Homogenizer 850 (Thermo Fisher). Mouse squamous organoids were mechanically dissociated from Matrigel, dispersed using 0.05% Trypsin-EDTA (Thermo Fisher) and manual pipetting. RNA was isolated using RNeasy Mini Kit (QIAGEN), following the manufacturer's protocol. For microarray, samples were processed and hybridized to Affymetrix Mouse Gene 2.0 ST by the Washington University Genome Technology Access Core (GTAC). Mouse forestomach tissue GeneChips were analyzed with Partek Flow Genomic Analysis Software using default settings for array QC, data normalization, and to generate differential expression analyses including hierarchical clustering and volcano plots. The mouse forestomach tissue microarray data is available from the GEO repository as GSE297858. Mouse forestomach organoid GeneChips were analyzed with Transcriptome Analysis Console (Applied Biosystems) analysis software to perform array QC, data normalization, and differential expression analyses. The mouse forestomach organoid microarray data is available from the GEO repository as GSE297930. Gene Set Enrichment Analysis (GSEA) (5, 6) was done using default settings with GMX files acquired from GSEA molecular signatures database (hallmark gene sets and cell type signature gene sets).

Mouse Squamous Organoid Matrigel Growth and Area Assay

Squamous organoids were mechanically dissociated from Matrigel, dispersed using 0.05% Trypsin-EDTA (Thermo Fisher) and manual pipetting, and 25,000 cells were transferred into new Matrigel and plated in 24-well plates. Serial organoid imaging and image stitching was performed using BioTek Cytation 7 Cell Imaging Multimode Reader and Gen5 Software. Total organoid area per well was calculated using ImageJ. Area under the curve (AUC) measurements were calculated at day 17 using Prism 10 (GraphPad). At day 14, areas of squamous organoids were manually calculated using ImageJ with "Total Organoid Area" determined as the maximal cross-sectional area including the central keratinization area and "Cellular Organoid Area" being the maximal cross-sectional area excluding the central keratinization area.

Mouse Squamous Organoid Transwell Proliferation and Maturation Assay

Squamous organoids were mechanically dissociated from Matrigel, dispersed using 0.05% Trypsin-EDTA (Thermo Fisher) and manual pipetting, and 200,000 cells were platted atop Collagen IV (Sigma-Alrich) coated 6.5 mm COSTAR 3470 transwell inserts (Corning). Proliferation condition was defined as supplying KSFM media with supplements on the transwell plated squamous organoids. Maturation condition was defined as removal of media from the transwell plated squamous organoids to allow an air-liquid interface. The barrier integrity of squamous organoids grown in transwells was determined with trans-epithelial electrical resistance (TEER) as measured with an epithelial Volt/Ohm meter (Millipore MERS 000–01). Transwell trans-epithelial electrical

resistance was measured on day 3 after transwell plating. On day 8, air liquid interface maturation conditions were initiated until day 19. Area under the curve (AUC) measurements were calculated for proliferation conditions (day 3-8) and maturation conditions (day 9-19) using Prism 10 (GraphPad).

Mouse Squamous Organoid CUT&RUN

Mouse squamous organoids were mechanically dissociated from Matrigel, dispersed using 0.05% Trypsin-EDTA (Thermo Fisher) and manual pipetting. 400,000 cells were used per condition according to the CUTANA CUT&RUN Kit (EpiCypher). Briefly, ConA beads were activated and dissociated cells were bound to beads and incubated with antibodies (0.1 µg H3K27me3, MA5-11198, Invitrogen; 0.5 µg SOX2, ab97959, Abcam; IgG 0.5 µg, 13-0042, EpiCypher) on nutator overnight at 4°C. The slurry was washed with cold digitonin (0.01%) buffer prior to binding of pAG-MNase (EpiCypher) and incubation for 10 minutes at room temperature. 100 mM of $CaCl_2$ was added to activate pAG-MNase for 2 hours at 4°C prior to stop buffer chelating of calcium and DNA purification with the CUTANA DNA Purification Kit. Illumina NGS libraries were prepared using 5 ng of DNA from each condition and the CUTANA CUT&RUN Library Prep Kit. Agilent TapeStation was used to calculate molarity of DNA fragments. DNA libraries were pooled for a final concentration of 10 nM. Library QC and sequencing was performed on a NovaSeq PE150 (Illumina) by Novogene.

Data analysis was performed on Galaxy platform(18) using Cutadapt for adapter and bad quality base removal, Bowtie2 for mapping to reference genome GRCm38 (mm10). MACS2 was performed for peak calling. Mouse forestomach organoid

CUT&RUN sequencing data is available as GSE297942. Heatmaps were plotted using Galaxy and Integrative Genomics Viewer was used for visualization. MEME suite (version 5.5.7) (19) with MEME-ChIP (20) and STREME (21) algorithms was used for peak motif analysis. Genomic Regions Enrichment of Annotations Tool (GREAT) (22) was used to associate peak genomic regions with target genes. Gene Ontology biological analyses of statistical overrepresentation testing for GO biological processes complete was performed for SOX2 activated and repressed transcriptional gargets using PANTHER (Protein ANalysis THrough Evolutionary Relationships) (8, 9).

Mouse Squamocolumnar Junction Area Calculations

Mouse forestomach tissues from untreated, 1 month DOC treated, and >6 month DOC treated wildtype control and *Sox2*$^{\Delta/\Delta}$ mice were dissected into strips, fixed, processed in paraffin and sectioned as described above. Immunofluorescence staining was performed as detailed above for CK7 to demarcate the transitional glands at the squamocolumnar junction (23, 24). CK7 positively stained squamocolumnar junction areas from 3-7 different squamocolumnar regions per mouse were measured using ImageJ.

Mouse Squamocolumnar Junction Inflammatory Cell Quantifications

Tissue sections were stained using the Bond RX^{m} as described above and imaged with an AxioScan.Z1 whole tissue scanner and ZEN v3 software (Zeiss). Unbiased parallelized analysis was then performed with HALO v4 digital pathology (Indica Labs). First, section images were annotated to include tissue regions of interest (ROI) and

exclude artifacts and debris. For the “Cytonuclear v2” analysis module, first nuclear detection and cell segmentation were fine-tuned to identify cells by the hematoxylin counterstain. Then, stain colors and thresholds specified to either cytoplasmic or nuclear localization were measured across all sections to calculate cell frequencies by stain expression.

Imaging

Brightfield images were taken on a Nikon NiU Manual Upright Fluorescence microscope or Olympus BX43 microscope. Fluorescence microscopy was performed using a Zeiss Axiovert 200 microscope with an Axiocam MRM camera and Apotome II instrument for grid-based optical sectioning or a Nikon NiU Manual Upright Fluorescence Microscope. Confocal fluorescence microscopy was performed using a Zeiss LSM 980 confocal microscope. Images were analyzed and post-imaging adjustments were performed with Adobe Photoshop CC. Organoid brightfield and fluorescent imaging were performed on a BioTek Cytation 7 Cell Imaging Mulitmode Reader. Transmission EM sections were viewed on a JEOL-1230 microscope equipped with a 4k x 4k CCD camera.

Mouse Squamocolumnar Junction Spatial Proteomics through On-site Tissue-protein-labeling (25)

Urea, tandem mass tag (TMT) reagents and dithiothreitol (DTT) were purchased from Thermo Fisher. Sequencing-grade trypsin was purchased from Promega. Lys-C was purchased from Wako Chemicals. C18 StageTips were packed in-house using two layers of 47 mm Empore C18 disks from CDS. All other reagents, including iodoacetamide (IAA),

formic acid (FA), anhydrous acetonitrile (ACN), and Tris–HCl (pH 8.0) were purchased from Sigma-Aldrich.

Mouse tissue blocks were fixed and embedded in paraffin as previously described. Serial 5 µm section slides were cut from tissue blocks. Manual Alcian blue and Periodic Acid – Schiff (pH 2.5) staining was performed by the Johns Hopkins Hospital Department of Surgical Pathology to illustrate the target area. Both H&E and Alcian blue and Periodic Acid – Schiff stained sections were used to identify the target area under a light microscope with various magnifications, including at low magnifications (e.g., 4x and 10x) for the evaluation of the overall tissue architecture and a high magnification (e.g., 40x) for the evaluation of the cytological characterization. The characteristics of targeted area were confirmed by a board-certified pathologist (QKL). The unstained slides were matched to corresponding H&E and Alcian blue and Periodic Acid – Schiff stained slides. 0.6 mm circles of target area were marked with a fine-tip marker pen (Sarstedt) on the back of each unstained slide.

For proteomic analysis, unstained slides were heated in an oven at 60°C for 10 minutes, and then incubated in xylene bath for deparaffinization followed by serial ethanol series and HPLC-grade water washes. Deparaffinized slides were then incubated in 100 mM Tris buffer pH 8.0 at 70°C for 20 minutes to decrosslink proteins, followed PBS and HPLC-grade water washes, and gently blow-dried with nitrogen gas. The target areas of dried slides were matched with the H&E and Alcian blue and Periodic Acid – Schiff stained slides, and a 0.6 mm circle was made to 4 squamocolumnar junctions from a wildtype control mouse and 4 squamocolumnar junctions from a *Sox2*$^{\Delta/\Delta}$ mouse.

The TMT-16-plex was used. Briefly, the TMT labeling reagents were diluted with 125mM HEPES at 1:4 ratio to the final concentration of 2 µg/µL in 100 mM HEPES and 20% ACN. The labeling reagent was directly applied to target circles on unstained slides using a pipette. The slides were air-dried. This labeling procedure was repeated 5 times and quenched using 5% hydroxylamine. After TMT labeling, tissues on unstained slides were scraped off using a scalpel and collected into vials for preparation of cell lysates.

Cell lysate preparation, protein extraction and digestion from above TMT-label tissue samples were performed as previously described (26). Briefly, each sample was lysed in lysis buffer containing 8 M urea, 75 mM NaCl, 50 mM Tris (pH 8.0), 1 mM EDTA, 2 µg/mL aprotinin, 10 µg/mL leupeptin, 1 mM PMSF, 10 mM NaF, phosphatase inhibitor cocktail 2 and 3 [1:100 dilution], and 20 µM PUGNAc. Proteins were reduced and alkylated with DTT (5 mM, 37ºC, 1 hr) and IAA (10 mM, room temperature for 45 min in the dark). The reduced proteins were diluted 1:4 with 50 mM Tris–HCl (pH 8.0) and incubated with Lys-C for 2 hr followed by trypsin digestion with enzyme/substrate ratio of 1:49 (wt/wt) for overnight digestion at 25ºC on a shaker. The digestion was quenched by adjusting pH to < 3 with 50% of formic acid. The digested peptide samples were desalted C18-StageTips and dried using Speed-Vac (26). Dried samples were kept at -80ºC for future analysis.

Proteomic analyses were performed in the Mass Spectrometry Core Facility at the Johns Hopkins Biomarker Discovery and Translation Center. The Orbitrap Exploris 480 mass spectrometer (Thermo Fisher) coupled with EvosepOne (Evosep Biosystems) LC system was used. Global peptides were loaded onto Evotip using 0.1% FA and separated on 15 cm x 150 µm, 1.5 µm PepSep C18 column (Bruker) in a Bruker column toaster

(50ºC) at an 88 min/30 SPD gradient. The peptides were separated, eluted from the column and nanosprayed directly into the mass spectrometer in a data-dependent mode.

Parameters for global proteomic samples were set as follows: MS1 resolution–60,000, mass range–350 to 1650m/z, RF Lens–60%, Normalized AGC Target (%)–300, Max injection time–auto, charge state include–2–6, dynamic exclusion–45s. The cycle time was set to 3 s, and within this 3 s the most abundant ions per scan were selected for MS/MS in the orbitrap. MS2 resolution–30,000, high-energy collision dissociation activation energy–37, isolation width (m/z)–0.7, AGC Target–standard, max injection time–64 ms.

All raw files were processed through MS-PyCloud (27) that were converted into mzML and searched against *Mus musculus* protein sequence FASTA files from UniProt/Swiss-Prot via MS-GF+ using the following settings: fixed modification of carbamidomethyl at cysteine, dynamic modifications of oxidation at methionine and TMT at lysine and protein N-terminus, precursor mass tolerance of 20 ppm, missed cleavages ≤ 2, instrument ID of "High-res LTQ," and fragmentation method of HCD. A false discovery rate of 1% at the PSM level with a minimum of 1 PSM per peptide and a minimum of 1 peptide per protein were used for data analysis. Protein abundances were calculated by summing up the abundances of peptide spectral matches belonging to the same protein. Proteins with more than 50% missing values were not included. For differential analysis, median-normalized datasets were further log2 transformed. Positive and negative log2-fold changed proteins were identified as differentially expressed. Mouse squamocolumnar junction spatial proteomics data can be accessed via EBI's PRIDE repository available as PXD063992. Gene Ontology biological analyses of statistical overrepresentation

testing for GO biological processes complete was performed on these differentially expressed proteins using PANTHER (Protein ANalysis THrough Evolutionary Relationships) (8, 9). Gene Set Enrichment Analysis (GSEA) (5, 6) was done using default settings with GMX files generated from The Human Tissue Specific Proteome database (Human Protein Atlas proteinatlas.org) (28).

SUPPLEMENTAL REFERENCES

1. Zhang Q, et al. A human Barrett's esophagus organoid system reveals epithelial-mesenchymal plasticity induced by acid and bile salts. *Am J Physiol Gastrointest Liver Physiol.* 2022;322(6):G598-G614.
2. Jin RU, et al. Tropism of Severe Acute Respiratory Syndrome Coronavirus 2 for Barrett's Esophagus May Increase Susceptibility to Developing Coronavirus Disease 2019. *Gastroenterology.* 2021;160(6):2165-8 e4.
3. Kasagi Y, et al. The Esophageal Organoid System Reveals Functional Interplay Between Notch and Cytokines in Reactive Epithelial Changes. *Cell Mol Gastroenterol Hepatol.* 2018;5(3):333-52.
4. Nakagawa H, et al. Modeling Epithelial Homeostasis and Reactive Epithelial Changes in Human and Murine Three-Dimensional Esophageal Organoids. *Curr Protoc Stem Cell Biol.* 2020;52(1):e106.
5. Mootha VK, et al. PGC-1alpha-responsive genes involved in oxidative phosphorylation are coordinately downregulated in human diabetes. *Nat Genet.* 2003;34(3):267-73.
6. Subramanian A, et al. Gene set enrichment analysis: a knowledge-based approach for interpreting genome-wide expression profiles. *Proc Natl Acad Sci U S A.* 2005;102(43):15545-50.
7. Wang S, et al. Transcriptional profiling suggests that Barrett's metaplasia is an early intermediate stage in esophageal adenocarcinogenesis. *Oncogene.* 2006;25(23):3346-56.
8. Mi H, et al. PANTHER in 2013: modeling the evolution of gene function, and other gene attributes, in the context of phylogenetic trees. *Nucleic Acids Res.* 2013;41(Database issue):D377-86.
9. Thomas PD, et al. PANTHER: Making genome-scale phylogenetics accessible to all. *Protein Sci.* 2022;31(1):8-22.
10. Van Keymeulen A, et al. Distinct stem cells contribute to mammary gland development and maintenance. *Nature.* 2011;479(7372):189-93.
11. Shaham O, et al. Pax6 is essential for lens fiber cell differentiation. *Development.* 2009;136(15):2567-78.

12. Madisen L, et al. A robust and high-throughput Cre reporting and characterization system for the whole mouse brain. *Nat Neurosci.* 2010;13(1):133-40.
13. Vasioukhin V, et al. The magical touch: genome targeting in epidermal stem cells induced by tamoxifen application to mouse skin. *Proc Natl Acad Sci U S A.* 1999;96(15):8551-6.
14. Huh WJ, et al. Inducible activation of Cre recombinase in adult mice causes gastric epithelial atrophy, metaplasia, and regenerative changes in the absence of "floxed" alleles. *Am J Physiol Gastrointest Liver Physiol.* 2010;299(2):G368-80.
15. Munch NS, et al. High-Fat Diet Accelerates Carcinogenesis in a Mouse Model of Barrett's Esophagus via Interleukin 8 and Alterations to the Gut Microbiome. *Gastroenterology.* 2019;157(2):492-506 e2.
16. Quante M, et al. Bile acid and inflammation activate gastric cardia stem cells in a mouse model of Barrett-like metaplasia. *Cancer Cell.* 2012;21(1):36-51.
17. Molendijk J, et al. Chronic High-Fat Diet Induces Early Barrett's Esophagus in Mice through Lipidome Remodeling. *Biomolecules.* 2020;10(5).
18. Galaxy C. The Galaxy platform for accessible, reproducible, and collaborative data analyses: 2024 update. *Nucleic Acids Res.* 2024;52(W1):W83-W94.
19. Bailey TL, et al. The MEME Suite. *Nucleic Acids Res.* 2015;43(W1):W39-49.
20. Machanick P, and Bailey TL. MEME-ChIP: motif analysis of large DNA datasets. *Bioinformatics.* 2011;27(12):1696-7.
21. Bailey TL. STREME: accurate and versatile sequence motif discovery. *Bioinformatics.* 2021;37(18):2834-40.
22. McLean CY, et al. GREAT improves functional interpretation of cis-regulatory regions. *Nat Biotechnol.* 2010;28(5):495-501.
23. Jiang M, et al. Transitional basal cells at the squamous-columnar junction generate Barrett's oesophagus. *Nature.* 2017;550(7677):529-33.
24. Wang X, et al. Residual embryonic cells as precursors of a Barrett's-like metaplasia. *Cell.* 2011;145(7):1023-35.
25. Xu Y, et al. SPOT: spatial proteomics through on-site tissue-protein-labeling. *Clin Proteomics.* 2024;21(1):60.
26. Mertins P, et al. Reproducible workflow for multiplexed deep-scale proteome and phosphoproteome analysis of tumor tissues by liquid chromatography-mass spectrometry. *Nat Protoc.* 2018;13(7):1632-61.
27. Hu Y, et al. MS-PyCloud: A Cloud Computing-Based Pipeline for Proteomic and Glycoproteomic Data Analyses. *Anal Chem.* 2024;96(25):10145-51.
28. Uhlen M, et al. Proteomics. Tissue-based map of the human proteome. *Science.* 2015;347(6220):1260419.

SUPPLEMENTAL FIGURE LEGENDS

Supplemental Figure 1. *SOX2* expression correlates with intestinal and esophageal gene signatures. Dot plots showing depicting Log2 gene expression of *SOX2* (X-axis) vs. **A**) Intestinal genes (*LYZ*, *TFF3*, *OLFM4*), and **B**) Esophageal squamous genes (*KRT13*, *DSG3*, *TP63*) in 12 BE organoids and 4 esophageal squamous (SQM) organoids. Pearson correlation coefficients (r) and p-values indicated. Groupings: Hindgut (blue), Transitional (green), Foregut (red).

Supplemental Figure 2. Ultrastructural changes in the forestomach squamous epithelium of *Sox2*$^{\Delta/\Delta}$ mice. A) Transmission electron microscopy (TEM) of wildtype control forestomachs at indicated magnifications showing surface keratin, basal cells, and cell-cell junctions (arrowheads), Colored outlines denote areas shown at higher magnification. **B**) *Sox2*$^{\Delta/\Delta}$ forestomachs reveal increased nucleated cells ('N') near surface keratin and inclusions (white arrowheads) within the surface keratin (green panels), expanded cell-cell junctions (yellow arrowheads), autophagosomes (blue, purple and orange panels), electron-dense intracellular inclusions (purple panels), and increased basal cells (orange and red panels). Presented TEMs are differing forestomach regions from the same *Sox2*$^{\Delta/\Delta}$ or control animal.

Supplemental Figure 3. Esophageal phenotype of *Sox2*$^{\Delta/\Delta}$ mice. Immunostaining of esophagi from wildtype control and *Sox2*$^{\Delta/\Delta}$ mice showing SOX2 (green), lineage-traced cells (tdTomato, red), Ki-67 (white nuclear), and Cytokeratin 13 (purple). Scale bars: 100 µm. Images are representative of at least 3 independent experiments.

Supplemental Figure 4. Characterization of *Krt14$^{CreER/+}$; Sox2$^{\Delta/\Delta}$; ROSA26$^{tdTomato/+}$* mice. **A**) H&E staining of esophagi and forestomachs from *Krt14$^{CreER/+}$; Sox2$^{\Delta/\Delta}$; ROSA26$^{tdTomato/+}$* mice and wildtype control. Immunohistochemistry showing mosaic **B**) SOX2 loss and **C**) tdTomato expression (mCherry antibody). Insets show magnified areas. Scale bars: 100 µm. Images are representative of at least 3 independent experiments.

Supplemental Figure 5. Additional characterization of *Sox2$^{\Delta/\Delta}$* squamous organoids. **A**) Immunostaining of *Sox2$^{\Delta/\Delta}$* esophageal organoids showing SOX2 (green nuclear), tdTomato (red cytoplastic), Ki-67 (white nuclear), and Cytokeratin 13 (purple cytoplasmic) expression. Scale bars: 100 µm. **B**) Quantification of Ki-67 positive cells as a percentage of total organoid cells in esophageal and forestomach organoids from wildtype and *Sox2$^{\Delta/\Delta}$* mice. Means (red bar) ± SDs (black bars) shown. Two-tailed unpaired Student's *t*-test p-values indicated. **C**) *Top*: Brightfield images of control and *Sox2$^{\Delta/\Delta}$* forestomach organoids after 14 days of growth in Matrigel. Insets highlight squamous organoids with "Total Organoid Area" and "Cellular Organoid Area" shaded in green. Scale bars: 1000 µm. *Bottom*: Quantification of Total and Cellular Organoid Area; each dot represents one organoid. Mean (red bar) ± SD (black bars). Two-tailed unpaired Student's *t*-test p-values indicated. **D**) Quantification of Ki-67 positive cells per high-power field under proliferation and maturation conditions for control and *Sox2$^{\Delta/\Delta}$* forestomach organoids. Mean ± SD shown. Statistical significance determined by two-way ANOVA with

Tukey's post hoc test for condition and genotype effects with p-values indicated. Images are representative of at least 3 independent experiments.

Supplemental Figure 6. Increased mortality in *Sox2*$^{\Delta/\Delta}$ mice exposed to bile acid. Kaplan-Meier survival curve for wildtype control (blue) and *Sox2*$^{\Delta/\Delta}$ (red) mice exposed to 0.3% deoxycholate (DOC) at pH 7.0 in drinking water for 1 week followed by maintenance 0.1% DOC. Number of mice per group indicated.

Supplemental Figure 7. Inflammatory cell infiltration at the squamocolumnar junction in *Sox2*$^{\Delta/\Delta}$ mice. A) H&E staining of the squamocolumnar junction (SCJx) in wildtype control and *Sox2*$^{\Delta/\Delta}$ mice showing inflammatory infiltrates in *Sox2*-deficient SCJx. Insets: magnified views. **B**) Immunostaining of forestomach and SCJx regions for CD8, STING, F4/80, and Ly6G. Insets highlight inflammatory cell infiltration at the SCJx in *Sox2*$^{\Delta/\Delta}$ mice. Scale bars: 100 µm. **C**) Quantification of F4/80+ and Ly6G+ cells at the SCJx using HALO image analysis software. Groups: 3 untreated controls, 4 untreated *Sox2*$^{\Delta/\Delta}$ mice, 3 >6 month DOC-treated controls, and 4 >6 month DOC-treated *Sox2*$^{\Delta/\Delta}$ mice. Data shown as means (red bar) ± SEMs. Significance determined by two-way ANOVA with Tukey's post hoc test for effects of treatment and genotype with p-values indicated. Images are representative of at least 3 independent experiments.

Supplemental Figure 8. Squamous forestomach cells contribute to squamocolumnar gland expansion in *Sox2*$^{\Delta/\Delta}$ mice. Confocal images of forestomaches from 1-month DOC-treated wildtype control and *Sox2*$^{\Delta/\Delta}$ mice stained for

Cytokeratin 7 (green) and tdTomato (red). Insets show magnified squamocolumnar junctions; overlapping CK7+ and tdTomato+ cells are highlighted in *Sox2*$^{\Delta/\Delta}$ mice. Scale bars: 100 µm. Images are representative of at least 3 independent experiments.

Supplemental Table 1. Demographics of Barrett's esophagus organoids. Organoid ID, Age at time of procedure, Sex, Race, Prague Classification (if available from endoscopy report), Pathology, Proton Pump Inhibitor use, and indication of (*) Successful Organoid Generation as defined by ability to expand, freeze down, and characterize the organoid line.

Supplemental Table 2. Gene set enrichment analysis of the differentially expressed genes from control and *Sox2*$^{\Delta/\Delta}$ forestomachs. Gene set enrichment analysis using the "Hallmarks" and "Cell Signatures" gene sets with metrics shown including Gene Set (GS), Size, Enrichment Score (ES), Normalized Enrichment Score (NES), Nominal p-value (Nom p-val), False Discovery Rate q-value (FDR q-val), Family-Wise Error Rate p-value (FWER p-val), Rank at Max, and Leading Edge Statistics.

Supplemental Table 3. GO biological processes for SOX2 activated or repressed direct transcriptional targets. Top 30 decreased and increased GO biological processes based SOX2 activated or repressed transcriptional targets including Fold Enrichment and P-values.

Supplemental Table 4. GO biological processes based on the differentially expressed proteins in the squamocolumnar junctional glands of control and *Sox2*$^{\Delta/\Delta}$ animals. Top 20 decreased and increased GO biological processes based on the differentially expressed proteins in the squamocolumnar junctional glands of control and *Sox2*$^{\Delta/\Delta}$ animals including Fold Enrichment and P-values.

Supplemental Table 5. Gene set enrichment analysis of the differentially expressed proteins in the squamocolumnar junctional glands of control and *Sox2*$^{\Delta/\Delta}$ animals. Gene set enrichment analysis of the differentially expressed proteins in the squamocolumnar junctional gland of wildtype control and *Sox2*$^{\Delta/\Delta}$ animals with metrics shown including Gene Set (GS), Size, Enrichment Score (ES), Normalized Enrichment Score (NES), Nominal p-value (Nom p-val), False Discovery Rate q-value (FDR q-val), Family-Wise Error Rate p-value (FWER p-val), Rank at Max, and Leading Edge Statistics.

Supplemental Table 6. Primary antibody information.

Supplemental Figure 1

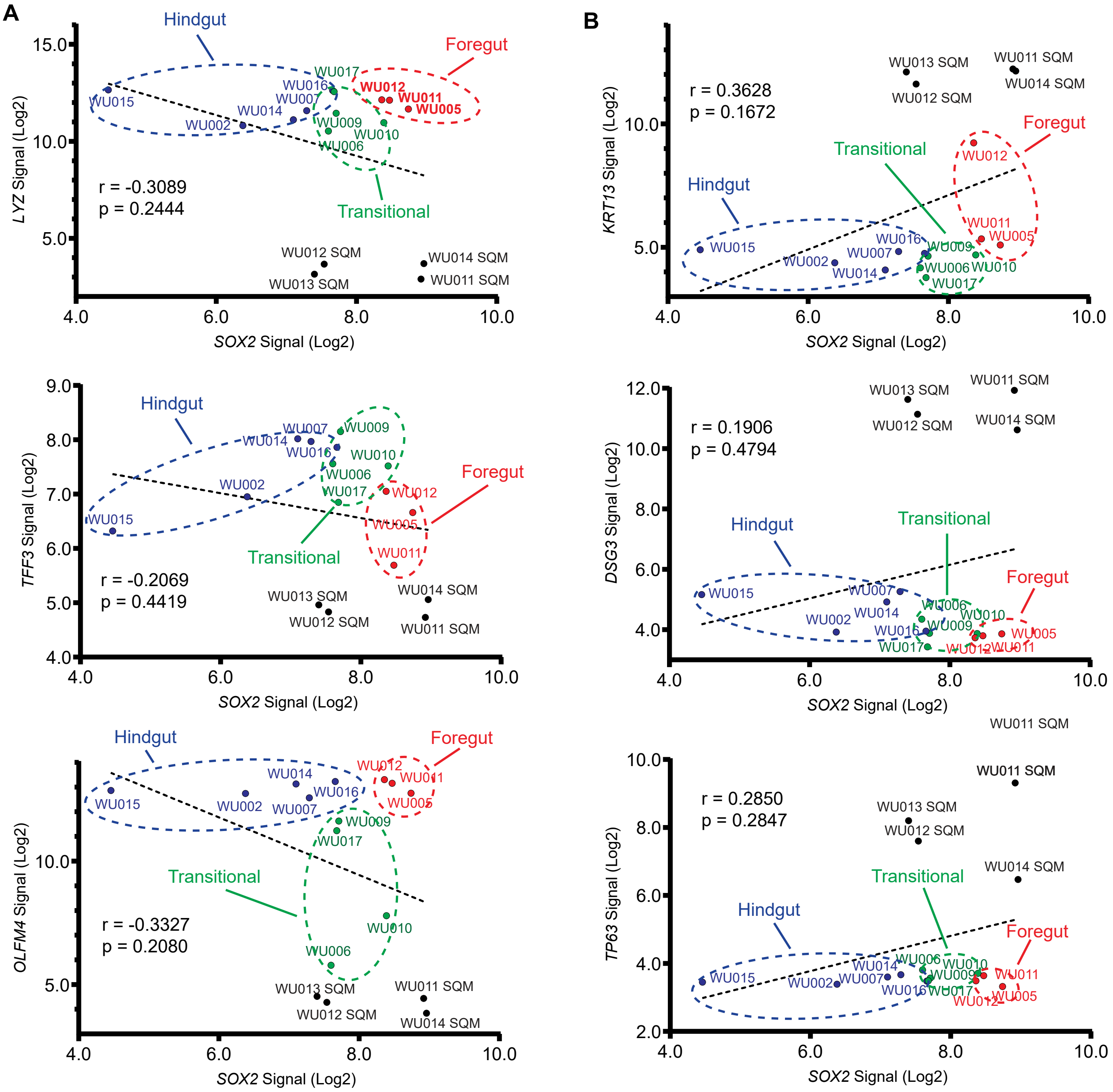

Supplemental Figure 2

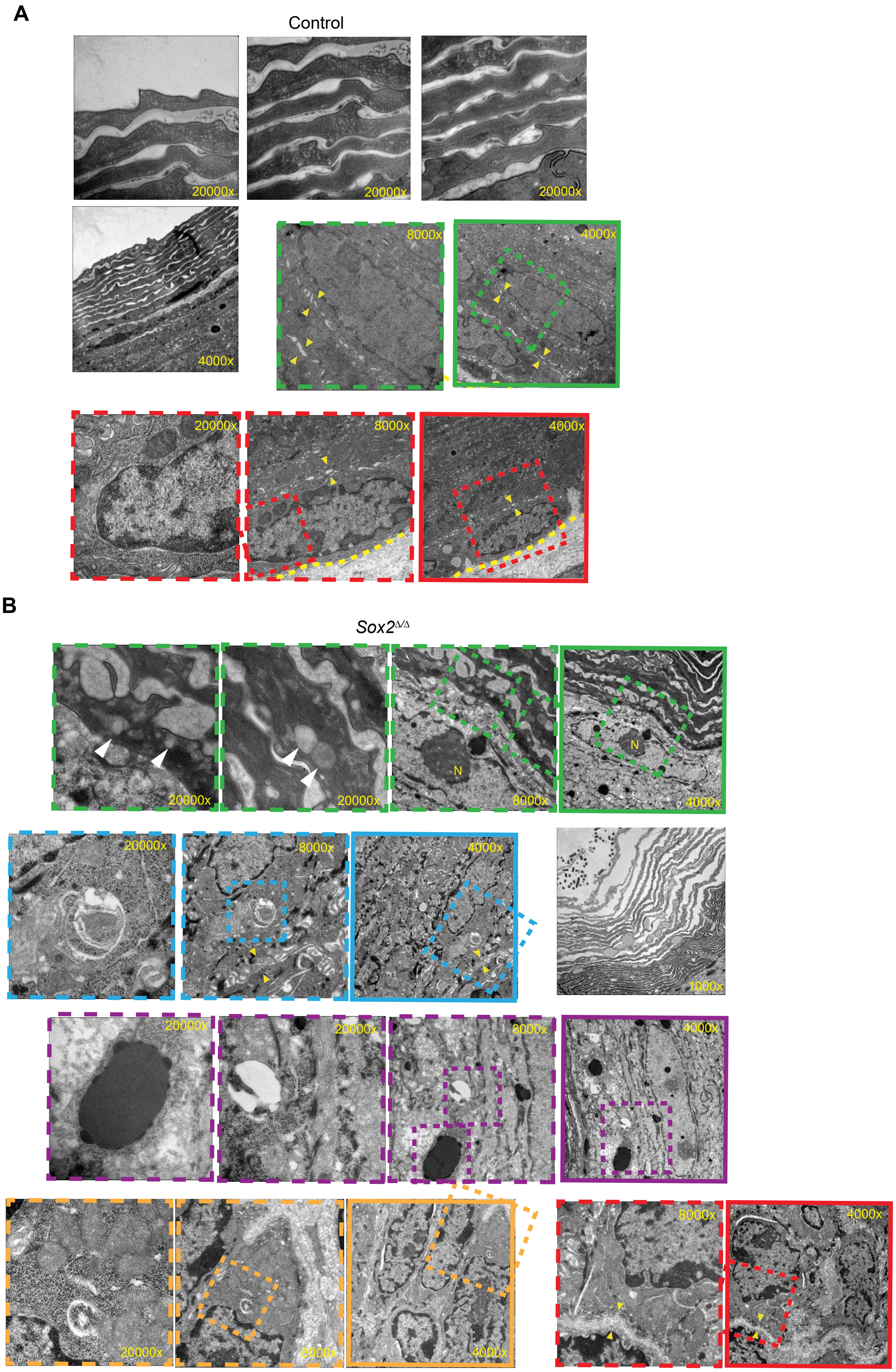

Supplemental Figure 3

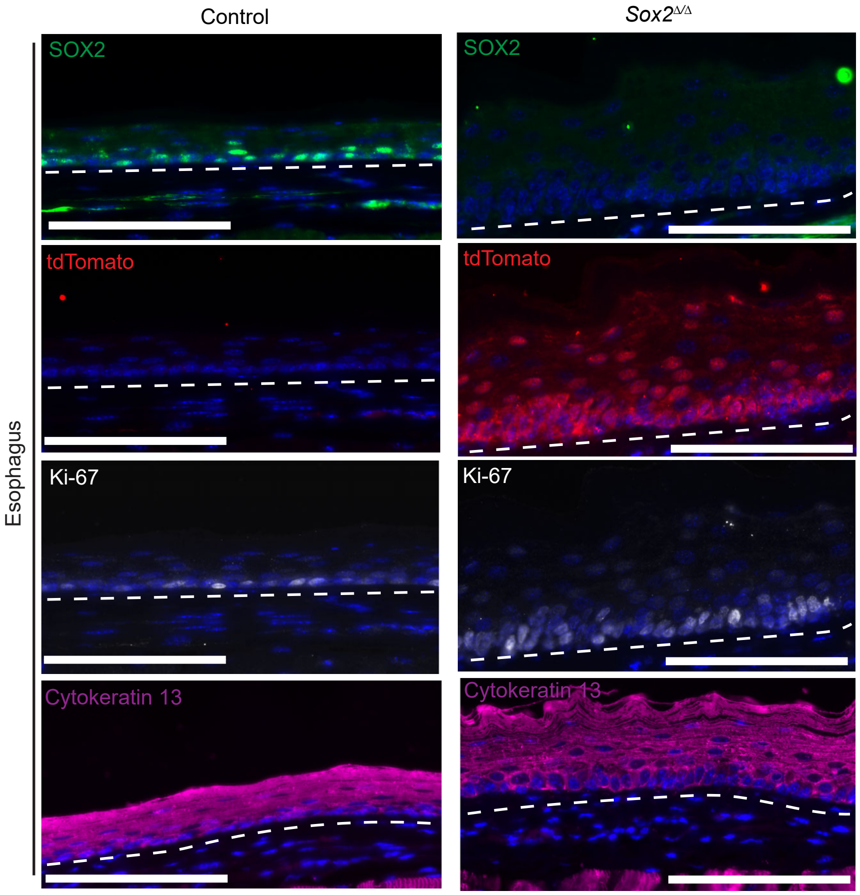

Supplemental Figure 4

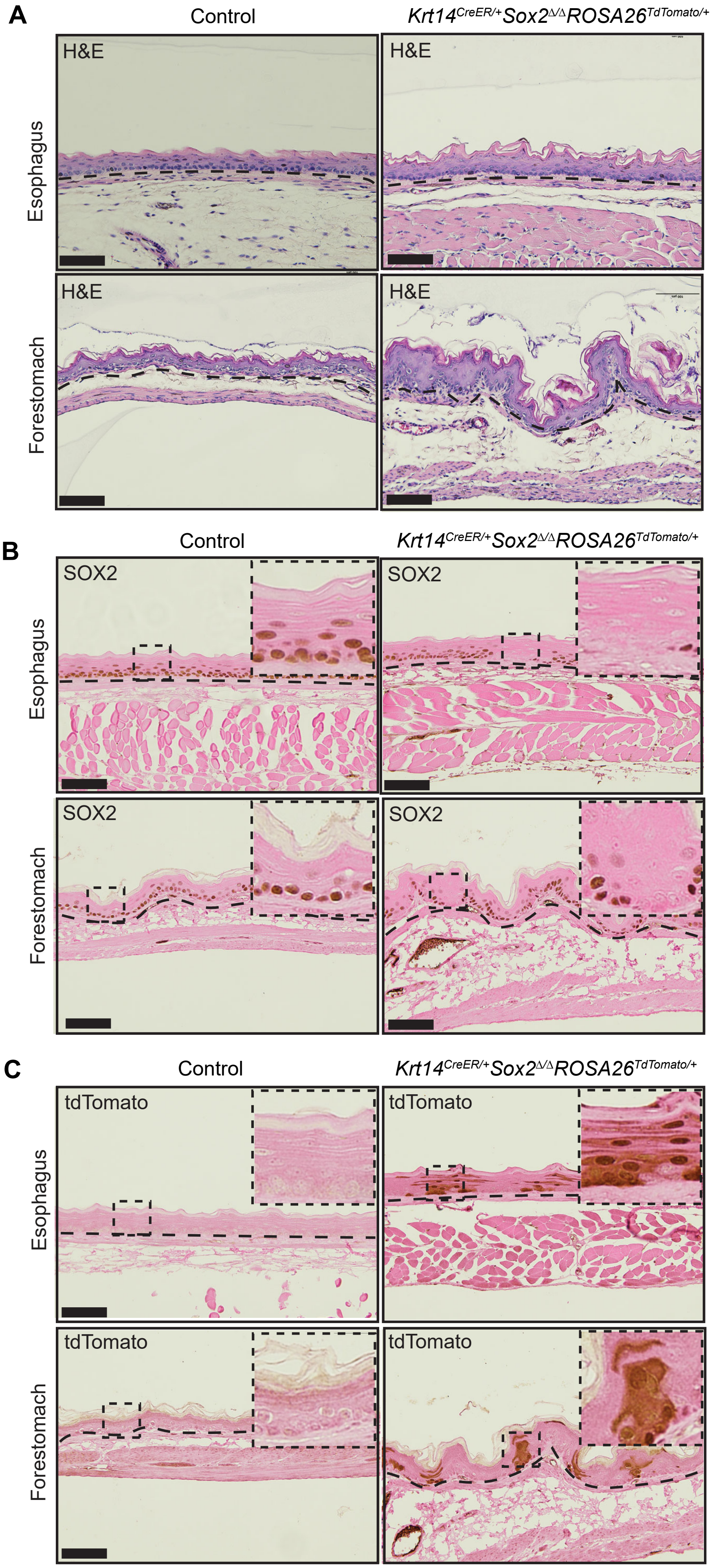

Supplemental Figure 5

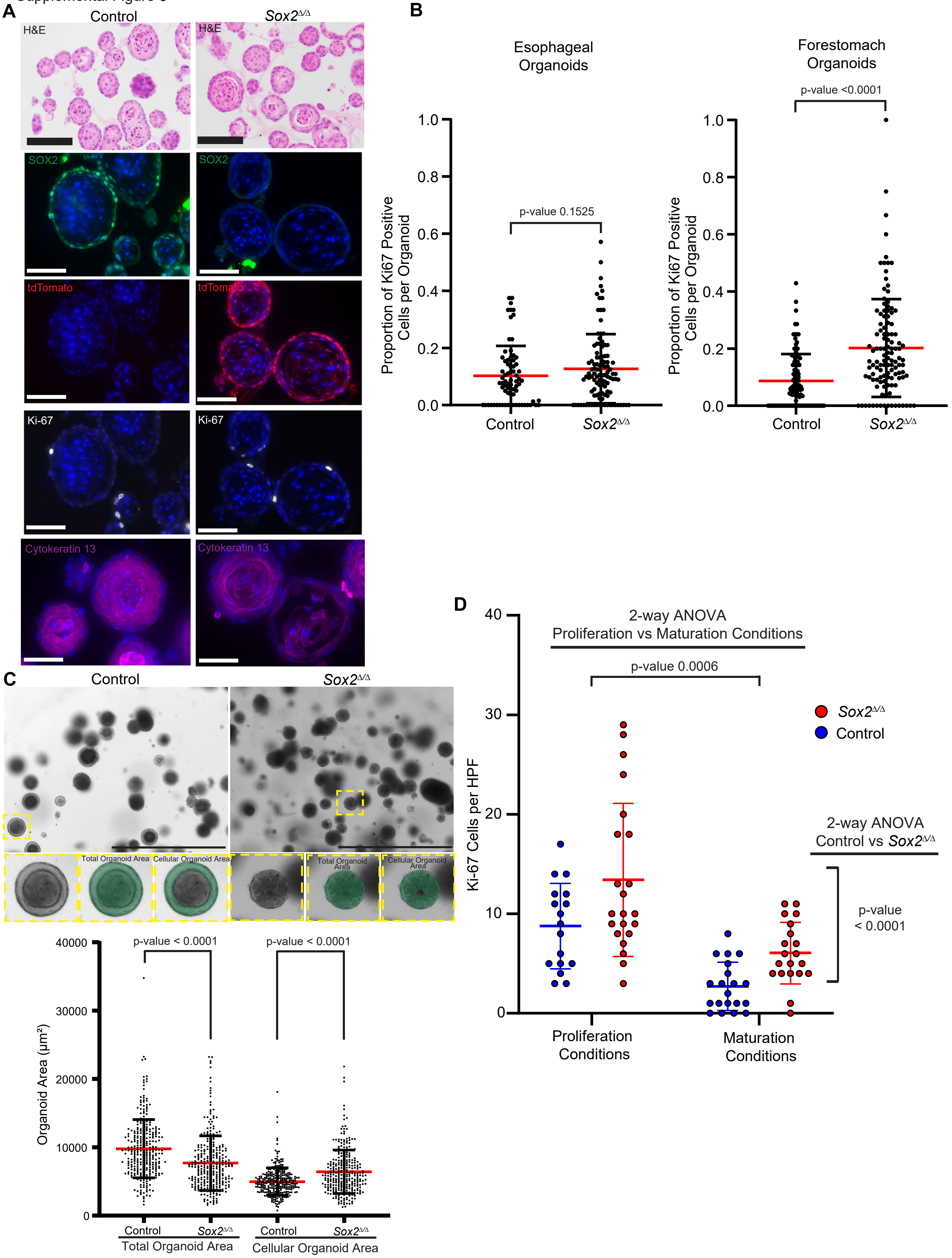

Supplemental Figure 6

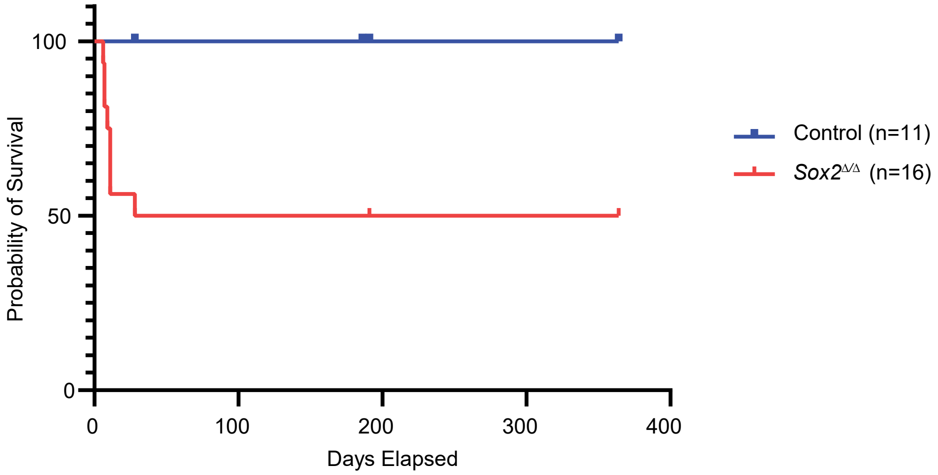

Supplemental Figure 7

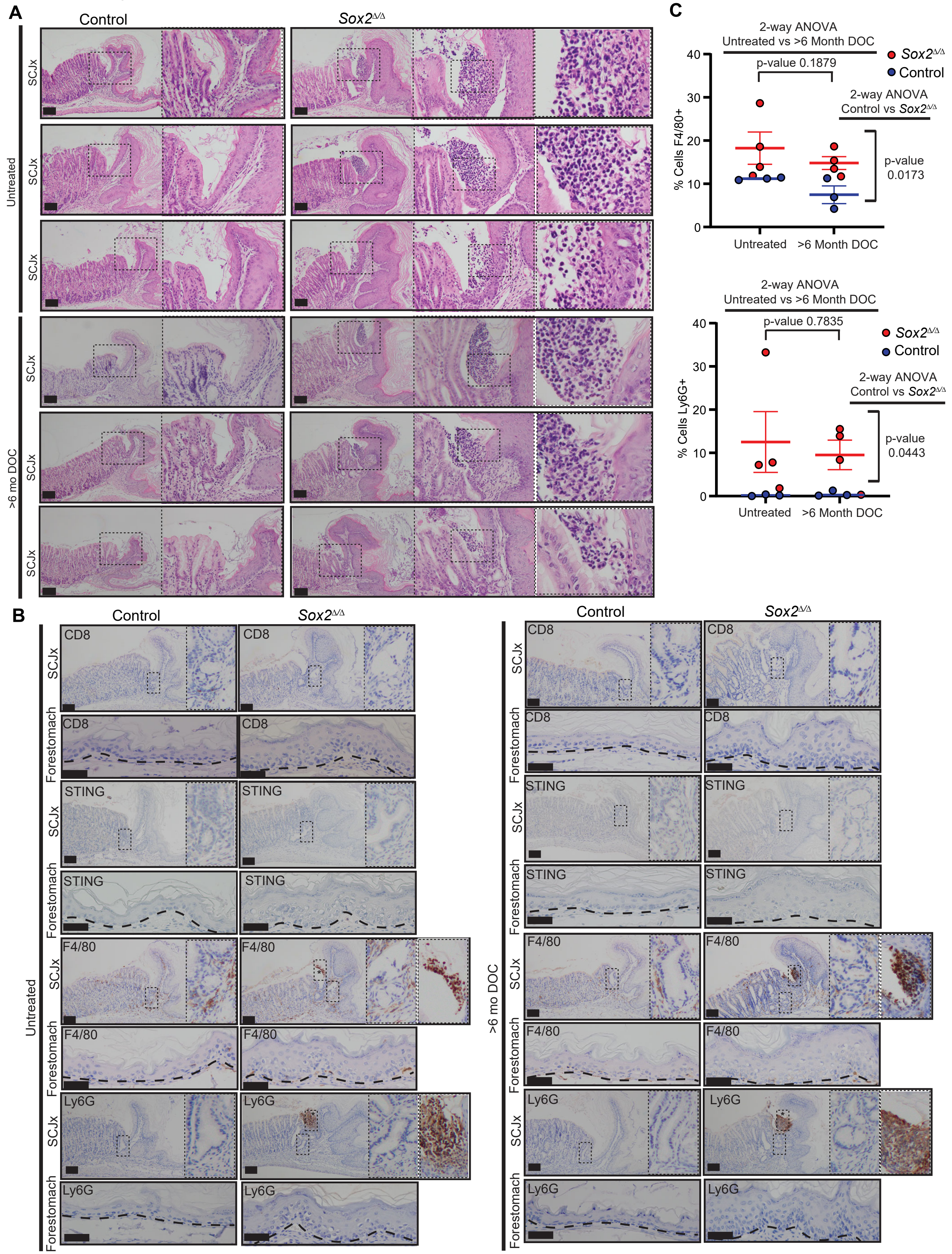

Supplemental Figure 8

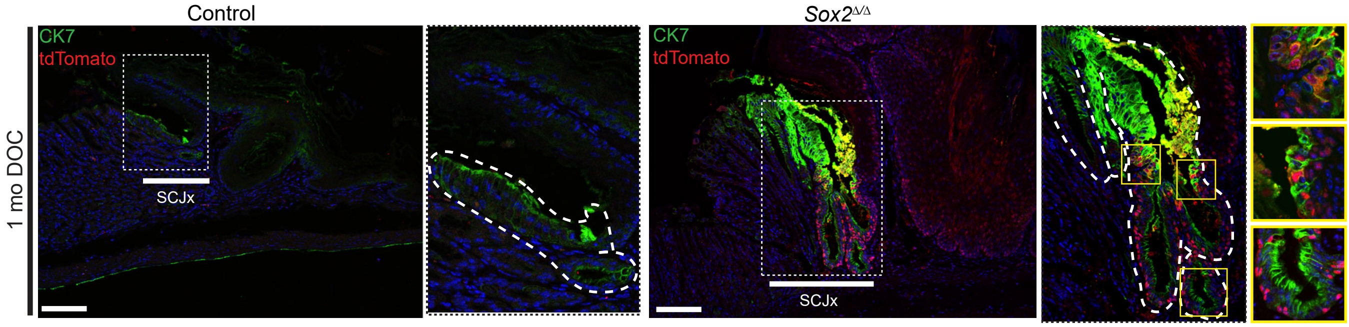

Supplemental Table 1. Demographics of Barrett's esophagus organoids

ID	Age	Sex	Race	Prague Classification	Pathology	Proton Pump Inhibitor	Successful Organoid Generation*
WU001	59	M	Caucasian	C10M12	Barrett's esophagus without dysplasia	Pantoprazole 40 mg BID	NO
WU002	84	M	Caucasian	C0M2	Barrett's esophagus without dysplasia	None	YES
WU004	70	M	Caucasian	C9M10	Barrett's esophagus without dysplasia	Esomeprazole 40 mg QD	NO
WU005	52	M	Caucasian	C0M2	Cardiac/Oxyntic mucosa with reactive changes, no Barrett's esophagus	Omeprazole 40 mg BID	YES
WU006	69	M	Caucasian	C1M4	Barrett's esophagus without dysplasia	Esomeprazole 40 mg BID	YES
WU007	43	F	Caucasian	C4M5	Barrett's esophagus without dysplasia	Omeprazole 40 mg QD	YES
WU008	56	M	Caucasian	Not provided	Barrett's esophagus without dysplasia	Omeprazole 40 mg BID	NO
WU009	69	M	Caucasian	C2M6	Barrett's esophagus without dysplasia	Omeprazole 20 mg QD	YES
WU010	71	F	Caucasian	C0M3	Barrett's esophagus indefinite for dysplasia	None	YES
WU011	35	M	Caucasian	C0M2	Squamocolumnar junction mucosa with focal Barrett's esophagus	None	YES
WU012	64	M	Caucasian	C0M2	Cardiooxyntic mucosa with chronic inflammation, no Barrett's esophagus	Esomeprazole 40 mg QD	YES
WU013	32	M	Caucasian	C0M3	Barrett's esophagus without dysplasia	Omeprazole 40 mg QD	NO
WU014	62	M	Caucasian	C1M3	Barrett's esophagus without dysplasia	Pantoprazole 40 mg BID	YES
WU015	61	M	Caucasian	C11M12	Barrett's esophagus without dysplasia	Pantoprazole 40 mg BID	YES
WU016	55	M	Caucasian	C10M12	Barrett's esophagus without dysplasia	Omeprazole 40 mg QD	YES
WU017	41	M	Caucasian	Not provided	Barrett's esophagus without dysplasia	None	YES
WU018	67	F	Caucasian	C1M3	Barrett's esophagus without dysplasia	Pantoprazole 40 mg BID	NO

Supplemental Table 2. Gene set enrichment analysis of the differentially expressed genes from control and *Sox2*$^{\Delta/\Delta}$ forestomachs

GSEA Report for *Sox2*$^{\Delta/\Delta}$ vs Control Forestomachs

HALLMARK Gene sets enriched in Control Forestomachs

	GS	SIZE	ES	NES	NOM p-val	FDR q-val	FWER p-val	RANK AT MAX	LEADING EDGE
1	HALLMARK_KRAS_SIGNALING_DN	191	0.31	1.31	0.041	0.867	0.537	1796	tags=15%, list=10%, signal=17%
2	HALLMARK_EPITHELIAL_MESENCHYMAL_TRANSITION	194	0.31	1.26	0.092	0.572	0.654	3417	tags=34%, list=19%, signal=41%
3	HALLMARK_HEME_METABOLISM	182	0.3	1.24	0.063	0.422	0.692	1275	tags=14%, list=7%, signal=15%
4	HALLMARK_ANGIOGENESIS	34	0.39	1.23	0.195	0.336	0.722	214	tags=9%, list=1%, signal=9%
5	HALLMARK_MYOGENESIS	192	0.29	1.22	0.085	0.288	0.75	1768	tags=20%, list=10%, signal=22%
6	HALLMARK_COAGULATION	131	0.3	1.19	0.115	0.282	0.811	1570	tags=14%, list=9%, signal=15%
7	HALLMARK_APICAL_SURFACE	44	0.34	1.12	0.28	0.343	0.904	45	tags=5%, list=0%, signal=5%
8	HALLMARK_UV_RESPONSE_DN	139	0.25	1.01	0.426	0.555	0.992	2818	tags=26%, list=16%, signal=31%
9	HALLMARK_PANCREAS_BETA_CELLS	40	0.3	0.96	0.477	0.65	0.996	2765	tags=25%, list=16%, signal=30%
10	HALLMARK_ANDROGEN_RESPONSE	94	0.25	0.94	0.584	0.621	0.998	1159	tags=15%, list=7%, signal=16%
11	HALLMARK_SPERMATOGENESIS	127	0.2	0.8	0.905	0.875	1	5076	tags=36%, list=29%, signal=50%

HALLMARK Gene sets enriched in *Sox2*$^{\Delta/\Delta}$ Forestomachs

	GS	SIZE	ES	NES	NOM p-val	FDR q-val	FWER p-val	RANK AT MAX	LEADING EDGE
1	HALLMARK_P53_PATHWAY	186	-0.54	-1.94	0	0	0	4316	tags=48%, list=24%, signal=63%
2	HALLMARK_MTORC1_SIGNALING	188	-0.51	-1.83	0	0.002	0.005	2820	tags=32%, list=16%, signal=38%
3	HALLMARK_OXIDATIVE_PHOSPHORYLATION	192	-0.47	-1.7	0	0.01	0.029	3931	tags=36%, list=22%, signal=46%
4	HALLMARK_MYC_TARGETS_V2	53	-0.55	-1.68	0.003	0.009	0.037	4034	tags=45%, list=23%, signal=58%
5	HALLMARK_HYPOXIA	197	-0.46	-1.66	0	0.009	0.046	2189	tags=27%, list=12%, signal=30%
6	HALLMARK_TNFA_SIGNALING_VIA_NFKB	188	-0.42	-1.51	0.001	0.05	0.279	4144	tags=39%, list=23%, signal=51%
7	HALLMARK_MYC_TARGETS_V1	177	-0.42	-1.48	0.002	0.059	0.356	4281	tags=41%, list=24%, signal=53%
8	HALLMARK_ESTROGEN_RESPONSE_EARLY	196	-0.41	-1.47	0	0.061	0.399	2473	tags=28%, list=14%, signal=32%
9	HALLMARK_PI3K_AKT_MTOR_SIGNALING	101	-0.42	-1.43	0.017	0.078	0.532	2954	tags=24%, list=17%, signal=28%
10	HALLMARK_PEROXISOME	102	-0.41	-1.4	0.024	0.1	0.66	2163	tags=22%, list=12%, signal=24%
11	HALLMARK_UNFOLDED_PROTEIN_RESPONSE	103	-0.41	-1.39	0.024	0.103	0.705	3771	tags=36%, list=21%, signal=45%
12	HALLMARK_GLYCOLYSIS	191	-0.37	-1.35	0.023	0.133	0.824	4363	tags=35%, list=25%, signal=46%
13	HALLMARK_ESTROGEN_RESPONSE_LATE	195	-0.37	-1.35	0.017	0.123	0.824	2473	tags=25%, list=14%, signal=28%
14	HALLMARK_DNA_REPAIR	141	-0.38	-1.32	0.046	0.146	0.893	4768	tags=40%, list=27%, signal=54%
15	HALLMARK_XENOBIOTIC_METABOLISM	189	-0.36	-1.3	0.055	0.169	0.938	3333	tags=31%, list=19%, signal=38%
16	HALLMARK_IL6_JAK_STAT3_SIGNALING	82	-0.39	-1.27	0.107	0.205	0.979	3895	tags=35%, list=22%, signal=45%
17	HALLMARK_CHOLESTEROL_HOMEOSTASIS	72	-0.39	-1.27	0.115	0.197	0.982	3590	tags=40%, list=20%, signal=50%
18	HALLMARK_ADIPOGENESIS	193	-0.34	-1.21	0.1	0.288	0.995	4170	tags=36%, list=23%, signal=47%
19	HALLMARK_FATTY_ACID_METABOLISM	153	-0.34	-1.21	0.13	0.274	0.995	3715	tags=31%, list=21%, signal=39%
20	HALLMARK_APOPTOSIS	155	-0.34	-1.21	0.129	0.266	0.996	3559	tags=26%, list=20%, signal=32%
21	HALLMARK_BILE_ACID_METABOLISM	110	-0.34	-1.15	0.224	0.39	0.999	2174	tags=17%, list=12%, signal=20%
22	HALLMARK_G2M_CHECKPOINT	190	-0.31	-1.12	0.247	0.463	1	3198	tags=26%, list=18%, signal=32%
23	HALLMARK_E2F_TARGETS	192	-0.3	-1.08	0.305	0.558	1	3740	tags=26%, list=21%, signal=32%
24	HALLMARK_UV_RESPONSE_UP	147	-0.3	-1.07	0.343	0.558	1	3248	tags=26%, list=18%, signal=31%
25	HALLMARK_INFLAMMATORY_RESPONSE	192	-0.29	-1.06	0.356	0.557	1	3932	tags=24%, list=22%, signal=31%
26	HALLMARK_IL2_STAT5_SIGNALING	191	-0.29	-1.06	0.367	0.547	1	4832	tags=35%, list=27%, signal=48%
27	HALLMARK_COMPLEMENT	184	-0.29	-1.05	0.364	0.537	1	2423	tags=16%, list=14%, signal=18%
28	HALLMARK_WNT_BETA_CATENIN_SIGNALING	39	-0.36	-1.04	0.396	0.538	1	3742	tags=28%, list=21%, signal=36%
29	HALLMARK_HEDGEHOG_SIGNALING	36	-0.36	-1.04	0.407	0.528	1	4661	tags=42%, list=26%, signal=56%
30	HALLMARK_APICAL_JUNCTION	195	-0.28	-1.02	0.428	0.557	1	3066	tags=22%, list=17%, signal=26%
31	HALLMARK_INTERFERON_GAMMA_RESPONSE	181	-0.28	-1.02	0.429	0.551	1	4902	tags=37%, list=28%, signal=51%
32	HALLMARK_ALLOGRAFT_REJECTION	182	-0.26	-0.94	0.637	0.729	1	4208	tags=27%, list=24%, signal=35%
33	HALLMARK_INTERFERON_ALPHA_RESPONSE	87	-0.28	-0.93	0.594	0.727	1	4923	tags=36%, list=28%, signal=49%
34	HALLMARK_PROTEIN_SECRETION	93	-0.28	-0.92	0.624	0.734	1	2164	tags=16%, list=12%, signal=18%
35	HALLMARK_KRAS_SIGNALING_UP	194	-0.25	-0.91	0.671	0.732	1	3766	tags=26%, list=21%, signal=33%
36	HALLMARK_TGF_BETA_SIGNALING	52	-0.29	-0.91	0.611	0.72	1	4163	tags=25%, list=23%, signal=33%
37	HALLMARK_NOTCH_SIGNALING	31	-0.31	-0.87	0.673	0.783	1	3184	tags=26%, list=18%, signal=31%
38	HALLMARK_MITOTIC_SPINDLE	194	-0.21	-0.75	0.948	0.945	1	5271	tags=34%, list=30%, signal=48%
39	HALLMARK_REACTIVE_OXYGEN_SPECIES_PATHWAY	47	-0.23	-0.69	0.921	0.966	1	4974	tags=30%, list=28%, signal=41%

Supplemental Table 2. Gene set enrichment analysis of the differentially expressed genes from control and *Sox2*$^{\Delta/\Delta}$ forestomachs

GSEA Report for *Sox2*$^{\Delta/\Delta}$ vs Control Forestomachs

CELL SIGNATURE Gene sets enriched in Control Forestomachs

	GS	SIZE	ES	NES	NOM p-va	FDR q-val	FWER p-v	RANK AT MAX	LEADING EDGE
1	DESCARTES_MAIN_FETAL_GOBLET_CELLS	38	0.87	2.76	0	0	0	675	tags=42%, list=4%, signal=44%
2	BUSSLINGER_GASTRIC_NECK_CELLS	35	0.77	2.43	0	0	0	593	tags=26%, list=3%, signal=27%
3	BUSSLINGER_GASTRIC_IMMATURE_PIT_CELLS	150	0.6	2.41	0	0.001	0.001	954	tags=24%, list=5%, signal=25%
4	DURANTE_ADULT_OLFACTORY_NEUROEPITHELIUM_SUSTENTACULA	41	0.71	2.31	0	0.001	0.002	659	tags=37%, list=4%, signal=38%
5	BUSSLINGER_GASTRIC_METALLOTHIONEIN_CELLS	86	0.6	2.23	0	0.003	0.008	518	tags=26%, list=3%, signal=26%
6	BUSSLINGER_GASTRIC_MATURE_PIT_CELLS	199	0.53	2.17	0	0.011	0.03	666	tags=18%, list=4%, signal=18%
7	DURANTE_ADULT_OLFACTORY_NEUROEPITHELIUM_RESPIRATORY_S	21	0.75	2.09	0.003	0.023	0.072	1614	tags=48%, list=9%, signal=52%
8	DESCARTES_FETAL_PANCREAS_ERYTHROBLASTS	108	0.54	2.07	0	0.026	0.091	2056	tags=25%, list=12%, signal=28%
9	BUSSLINGER_GASTRIC_PREZYMOGENIC_CELLS	56	0.6	2.04	0	0.029	0.115	2508	tags=36%, list=14%, signal=41%
10	HE_LIM_SUN_FETAL_LUNG_C1_LATE_TIP_CELL	28	0.67	2.04	0.007	0.028	0.121	1	tags=4%, list=0%, signal=4%
11	DESCARTES_MAIN_FETAL_PARIETAL_AND_CHIEF_CELLS	96	0.53	2.02	0	0.03	0.142	753	tags=15%, list=4%, signal=15%
12	DURANTE_ADULT_OLFACTORY_NEUROEPITHELIUM_RESPIRATORY_(	129	0.5	2	0	0.033	0.168	1349	tags=18%, list=8%, signal=19%
13	DESCARTES_FETAL_STOMACH_PARIETAL_AND_CHIEF_CELLS	37	0.64	2	0	0.031	0.17	34	tags=11%, list=0%, signal=11%
14	BUSSLINGER_DUODENAL_BCHE_CELLS	32	0.62	1.96	0.007	0.042	0.24	509	tags=19%, list=3%, signal=19%
15	DESCARTES_MAIN_FETAL_SKELETAL_MUSCLE_CELLS	136	0.48	1.91	0	0.06	0.336	2898	tags=35%, list=16%, signal=42%
16	BUSSLINGER_DUODENAL_EC_CELLS	29	0.63	1.9	0	0.06	0.35	3747	tags=55%, list=21%, signal=70%
17	DESCARTES_FETAL_EYE_SKELETAL_MUSCLE_CELLS	137	0.48	1.88	0	0.069	0.409	1812	tags=26%, list=10%, signal=29%
18	LAKE_ADULT_KIDNEY_C20_COLLECTING_DUCT_INTERCALATED_CEL	143	0.46	1.87	0	0.069	0.427	2842	tags=34%, list=16%, signal=40%
19	TRAVAGLINI_LUNG_SIGNALING_ALVEOLAR_EPITHELIAL_TYPE_2_CEL	64	0.53	1.87	0	0.066	0.431	538	tags=11%, list=3%, signal=11%
20	DESCARTES_FETAL_LUNG_NEUROENDOCRINE_CELLS	74	0.52	1.86	0.004	0.067	0.454	23	tags=4%, list=0%, signal=4%
21	TRAVAGLINI_LUNG_ALVEOLAR_EPITHELIAL_TYPE_2_CELL	121	0.47	1.8	0	0.1	0.633	77	tags=5%, list=0%, signal=5%
22	DESCARTES_FETAL_ADRENAL_ERYTHROBLASTS	80	0.49	1.77	0	0.115	0.686	735	tags=15%, list=4%, signal=16%
23	FAN_EMBRYONIC_CTX_EX_2_EXCITATORY_NEURON	15	0.67	1.77	0.023	0.116	0.702	2366	tags=47%, list=13%, signal=54%
24	GAO_LARGE_INTESTINE_24W_C7_GOBLET_PROGENITOR	15	0.67	1.75	0.036	0.123	0.739	3882	tags=73%, list=22%, signal=94%
25	HU_FETAL_RETINA_MULLER	38	0.54	1.75	0.024	0.119	0.742	1469	tags=32%, list=8%, signal=34%
26	DESCARTES_FETAL_STOMACH_ENS_NEURONS	63	0.49	1.73	0.013	0.13	0.784	5699	tags=62%, list=32%, signal=91%
27	DURANTE_ADULT_OLFACTORY_NEUROEPITHELIUM_OLFACTORY_EN	105	0.46	1.72	0.004	0.134	0.811	2072	tags=29%, list=12%, signal=32%
28	DESCARTES_FETAL_ADRENAL_STROMAL_CELLS	120	0.44	1.7	0	0.143	0.845	3532	tags=40%, list=20%, signal=50%
29	DESCARTES_FETAL_HEART_ELF3_AGBL2_POSITIVE_CELLS	35	0.54	1.7	0.014	0.14	0.849	823	tags=20%, list=5%, signal=21%
30	MENON_FETAL_KIDNEY_3_STROMAL_CELLS	68	0.47	1.69	0.008	0.143	0.862	4001	tags=57%, list=23%, signal=74%
31	BUSSLINGER_GASTRIC_CHIEF_CELLS	48	0.51	1.67	0.017	0.151	0.88	401	tags=10%, list=2%, signal=11%
32	MENON_FETAL_KIDNEY_0_CAP_MESENCHYME_CELLS	72	0.45	1.66	0.009	0.159	0.9	2398	tags=36%, list=13%, signal=42%
33	BUSSLINGER_GASTRIC_D_CELLS	27	0.57	1.66	0.027	0.156	0.905	1986	tags=26%, list=11%, signal=29%
34	MURARO_PANCREAS_EPSILON_CELL	38	0.51	1.65	0.029	0.159	0.914	1657	tags=32%, list=9%, signal=35%
35	DESCARTES_FETAL_ADRENAL_MYELOID_CELLS	137	0.42	1.65	0.005	0.156	0.916	1574	tags=21%, list=9%, signal=23%
36	AIZARANI_LIVER_C24_EPCAM_POS_BILE_DUCT_CELLS_3	175	0.41	1.65	0	0.152	0.918	1344	tags=22%, list=8%, signal=23%
37	LAKE_ADULT_KIDNEY_C28_INTERSTITIUM	82	0.44	1.64	0.009	0.156	0.933	4001	tags=49%, list=23%, signal=63%
38	HE_LIM_SUN_FETAL_LUNG_C3_PRIMITIVE_ERYTHROCYTE	90	0.44	1.62	0	0.168	0.944	735	tags=11%, list=4%, signal=12%
39	FAN_EMBRYONIC_CTX_OPC	49	0.49	1.61	0.045	0.167	0.95	2663	tags=37%, list=15%, signal=43%
40	DURANTE_ADULT_OLFACTORY_NEUROEPITHELIUM_BOWMANS_GLA	26	0.54	1.61	0.057	0.164	0.951	609	tags=27%, list=3%, signal=28%
41	DESCARTES_MAIN_FETAL_MUC13_DMBT1_POSITIVE_CELLS	44	0.48	1.61	0.033	0.164	0.953	1276	tags=18%, list=7%, signal=20%
42	BUSSLINGER_GASTRIC_ISTHMUS_CELLS	423	0.36	1.61	0	0.163	0.956	1814	tags=18%, list=10%, signal=20%
43	DESCARTES_FETAL_STOMACH_NEUROENDOCRINE_CELLS	44	0.49	1.6	0.041	0.162	0.959	2761	tags=34%, list=16%, signal=40%
44	DESCARTES_FETAL_INTESTINE_ENS_NEURONS	132	0.4	1.6	0	0.164	0.967	5699	tags=52%, list=32%, signal=76%
45	DESCARTES_FETAL_LUNG_BRONCHIOLAR_AND_ALVEOLAR_EPITHEL	48	0.48	1.6	0.029	0.161	0.968	830	tags=10%, list=5%, signal=11%
46	DESCARTES_FETAL_PANCREAS_ENS_GLIA	58	0.47	1.59	0.014	0.162	0.971	537	tags=14%, list=3%, signal=14%
47	BUSSLINGER_GASTRIC_LYZ_POSITIVE_CELLS	84	0.44	1.59	0.009	0.159	0.971	593	tags=15%, list=3%, signal=16%
48	DURANTE_ADULT_OLFACTORY_NEUROEPITHELIUM_OLFACTORY_HC	16	0.6	1.58	0.047	0.163	0.976	623	tags=25%, list=4%, signal=26%
49	DESCARTES_FETAL_MUSCLE_SKELETAL_MUSCLE_CELLS	184	0.38	1.56	0	0.182	0.987	3075	tags=33%, list=17%, signal=40%
50	GAO_LARGE_INTESTINE_ADULT_CF_GOBLET_CELL_SUBTYPE_1	20	0.56	1.56	0.051	0.179	0.987	4224	tags=70%, list=24%, signal=92%
51	ZHENG_CORD_BLOOD_C9_GRANULOCYTE_MACROPHAGE_PROGENI	84	0.43	1.55	0.031	0.181	0.988	2760	tags=35%, list=16%, signal=41%
52	DESCARTES_FETAL_ADRENAL_SCHWANN_CELLS	112	0.41	1.55	0.005	0.185	0.99	537	tags=10%, list=3%, signal=10%
53	DESCARTES_FETAL_SPLEEN_MYELOID_CELLS	105	0.4	1.54	0.019	0.186	0.99	2393	tags=27%, list=13%, signal=31%
54	DESCARTES_FETAL_ADRENAL_CHROMAFFIN_CELLS	77	0.43	1.54	0.033	0.184	0.991	4343	tags=47%, list=24%, signal=62%
55	DESCARTES_FETAL_LIVER_ERYTHROBLASTS	56	0.44	1.53	0.04	0.188	0.993	1991	tags=25%, list=11%, signal=28%
56	GAO_LARGE_INTESTINE_ADULT_CJ_IMMUNE_CELLS	453	0.33	1.51	0	0.203	0.996	3240	tags=31%, list=18%, signal=37%
57	LAKE_ADULT_KIDNEY_C24_ENDOTHELIAL_CELLS_AEA_AND_DVR	137	0.38	1.51	0	0.205	0.996	3246	tags=40%, list=18%, signal=49%
58	DESCARTES_MAIN_FETAL_ENS_NEURONS	27	0.5	1.51	0.063	0.202	0.996	4173	tags=59%, list=23%, signal=77%
59	DESCARTES_FETAL_THYMUS_VASCULAR_ENDOTHELIAL_CELLS	118	0.39	1.51	0.026	0.2	0.996	3040	tags=31%, list=17%, signal=38%
60	HE_LIM_SUN_FETAL_LUNG_C1_GHRL_POS_NEUROENDOCRINE_CELI	144	0.38	1.5	0.005	0.202	0.996	2472	tags=24%, list=14%, signal=28%
61	DESCARTES_FETAL_PANCREAS_ISLET_ENDOCRINE_CELLS	121	0.39	1.5	0.018	0.204	0.997	3577	tags=36%, list=20%, signal=44%
62	DESCARTES_FETAL_STOMACH_PDE1C_ACSM3_POSITIVE_CELLS	22	0.52	1.49	0.075	0.205	0.997	2751	tags=23%, list=15%, signal=27%
63	DESCARTES_FETAL_PLACENTA_EXTRAVILLOUS_TROPHOBLASTS	56	0.45	1.49	0.044	0.202	0.997	2323	tags=21%, list=13%, signal=25%
64	CUI_DEVELOPING_HEART_CORONARY_VASCULAR_ENDOTHELIAL_CI	39	0.48	1.49	0.064	0.204	0.998	2573	tags=31%, list=14%, signal=36%
65	LAKE_ADULT_KIDNEY_C26_MESANGIAL_CELLS	165	0.36	1.47	0.006	0.226	0.998	3896	tags=40%, list=22%, signal=51%
66	AIZARANI_LIVER_C33_STELLATE_CELLS_2	121	0.37	1.45	0.019	0.245	1	3907	tags=42%, list=22%, signal=54%
67	ZHONG_PFC_C2_THY1_POS_OPC	25	0.49	1.45	0.115	0.245	1	2479	tags=36%, list=14%, signal=42%
68	DESCARTES_FETAL_INTESTINE_STROMAL_CELLS	33	0.47	1.44	0.087	0.242	1	4354	tags=48%, list=25%, signal=64%
69	DESCARTES_FETAL_EYE_PHOTORECEPTOR_CELLS	26	0.49	1.44	0.079	0.239	1	3937	tags=38%, list=22%, signal=49%
70	MENON_FETAL_KIDNEY_6_COLLECTING_DUCT_CELLS	90	0.38	1.44	0.04	0.238	1	1990	tags=23%, list=11%, signal=26%
71	HU_FETAL_RETINA_BIPOLAR	78	0.39	1.44	0.04	0.242	1	3211	tags=26%, list=18%, signal=31%
72	DURANTE_ADULT_OLFACTORY_NEUROEPITHELIUM_OLFACTORY_MI	49	0.42	1.43	0.078	0.242	1	3083	tags=39%, list=17%, signal=47%
73	DESCARTES_FETAL_INTESTINE_CHROMAFFIN_CELLS	87	0.39	1.43	0.048	0.241	1	2288	tags=24%, list=13%, signal=28%
74	DESCARTES_MAIN_FETAL_UNIPOLAR_BRUSH_CELLS	23	0.48	1.42	0.099	0.258	1	6306	tags=57%, list=36%, signal=88%
75	DESCARTES_FETAL_EYE_ASTROCYTES	29	0.47	1.41	0.092	0.256	1	1926	tags=24%, list=11%, signal=27%
76	DESCARTES_FETAL_HEART_VISCERAL_NEURONS	75	0.39	1.4	0.082	0.273	1	2353	tags=24%, list=13%, signal=28%
77	BUSSLINGER_DUODENAL_EARLY_IMMATURE_ENTEROCYTES	99	0.36	1.4	0.048	0.274	1	744	tags=15%, list=4%, signal=16%
78	HE_LIM_SUN_FETAL_LUNG_C7_LATE_SCHWANN_CELL	92	0.38	1.39	0.07	0.272	1	2936	tags=29%, list=17%, signal=35%
79	DESCARTES_FETAL_THYMUS_STROMAL_CELLS	128	0.36	1.39	0.034	0.27	1	3823	tags=38%, list=22%, signal=48%
80	CUI_DEVELOPING_HEART_C3_FIBROBLAST_LIKE_CELL	109	0.37	1.39	0.031	0.267	1	3907	tags=40%, list=22%, signal=51%
81	FAN_EMBRYONIC_CTX_BIG_GROUPS_EXCITATORY_NEURON	42	0.43	1.39	0.11	0.264	1	5013	tags=50%, list=28%, signal=69%
82	HE_LIM_SUN_FETAL_LUNG_C7_MFNG_POS_DBH_POS_NEURON_CEL	15	0.53	1.39	0.118	0.261	1	4059	tags=53%, list=23%, signal=69%
83	DESCARTES_FETAL_PANCREAS_ENS_NEURONS	126	0.36	1.39	0.058	0.26	1	4533	tags=44%, list=26%, signal=58%
84	DESCARTES_FETAL_LUNG_VISCERAL_NEURONS	172	0.34	1.39	0.028	0.262	1	4599	tags=40%, list=26%, signal=53%
85	DESCARTES_FETAL_STOMACH_ERYTHROBLASTS	83	0.39	1.38	0.056	0.271	1	2056	tags=23%, list=12%, signal=26%
86	LAKE_ADULT_KIDNEY_C21_COLLECTING_DUCT_INTERCALATED_CEL	99	0.36	1.38	0.067	0.268	1	2387	tags=30%, list=13%, signal=35%
87	CUI_DEVELOPING_HEART_C6_EPICARDIAL_CELL	182	0.33	1.37	0.012	0.276	1	2708	tags=32%, list=15%, signal=37%
88	DESCARTES_MAIN_FETAL_CHROMAFFIN_CELLS	15	0.53	1.37	0.127	0.273	1	1756	tags=40%, list=10%, signal=44%
89	DESCARTES_FETAL_PANCREAS_STROMAL_CELLS	21	0.47	1.37	0.114	0.273	1	4354	tags=62%, list=25%, signal=82%
90	HE_LIM_SUN_FETAL_LUNG_C0_ADVENTITIAL_FIBROBLAST	67	0.39	1.36	0.099	0.277	1	2049	tags=21%, list=12%, signal=24%
91	DESCARTES_FETAL_MUSCLE_SCHWANN_CELLS	108	0.35	1.36	0.065	0.275	1	3129	tags=31%, list=18%, signal=37%
92	TRAVAGLINI_LUNG_MUCOUS_CELL	109	0.35	1.36	0.042	0.273	1	1614	tags=23%, list=9%, signal=25%

93	DESCARTES_FETAL_INTESTINE_ENS_GLIA	45	0.42	1.36	0.122	0.274	1	525	tags=9%, list=3%, signal=9%
94	DESCARTES_FETAL_STOMACH_MUC13_DMBT1_POSITIVE_CELLS	79	0.38	1.35	0.069	0.282	1	2367	tags=25%, list=13%, signal=29%
95	AIZARANI_LIVER_C7_EPCAM_POS_BILE_DUCT_CELLS_2	209	0.32	1.35	0.026	0.285	1	1733	tags=20%, list=10%, signal=22%
96	DESCARTES_FETAL_ADRENAL_SYMPATHOBLASTS	94	0.35	1.34	0.064	0.289	1	2539	tags=28%, list=14%, signal=32%
97	DURANTE ADULT OLFACTORY NEUROEPITHELIUM GLOBOSE BASA	30	0.43	1.34	0.123	0.287	1	2864	tags=33%, list=16%, signal=40%
98	DESCARTES_FETAL_KIDNEY_STROMAL_CELLS	130	0.34	1.33	0.073	0.303	1	3687	tags=34%, list=21%, signal=42%
99	CUI_DEVELOPING_HEART_5TH_WEEK_ATRIAL_CARDIOMYOCYTE	37	0.43	1.33	0.139	0.3	1	754	tags=11%, list=4%, signal=11%
100	BUSSLINGER_GASTRIC_OXYNTIC_ENTEROCHROMAFFIN_LIKE_CELLS	83	0.36	1.32	0.085	0.301	1	2931	tags=30%, list=17%, signal=36%
101	DESCARTES_FETAL_EYE_STROMAL_CELLS	86	0.35	1.32	0.057	0.305	1	4125	tags=44%, list=23%, signal=57%
102	DESCARTES_FETAL_HEART_SATB2_LRRC7_POSITIVE_CELLS	90	0.35	1.32	0.086	0.304	1	3020	tags=26%, list=17%, signal=31%
103	MANNO_MIDBRAIN_NEUROTYPES_HSERT	419	0.29	1.32	0	0.302	1	3796	tags=32%, list=21%, signal=39%
104	RUBENSTEIN_SKELETAL_MUSCLE_ENDOTHELIAL_CELLS	121	0.34	1.31	0.071	0.311	1	2752	tags=31%, list=15%, signal=36%
105	DESCARTES_FETAL_STOMACH_ENS_GLIA	44	0.4	1.3	0.114	0.318	1	3603	tags=43%, list=20%, signal=54%
106	DESCARTES_FETAL_INTESTINE_INTESTINAL_EPITHELIAL_CELLS	203	0.31	1.3	0.025	0.317	1	1419	tags=17%, list=8%, signal=19%
107	DESCARTES_FETAL_SPLEEN_ERYTHROBLASTS	56	0.37	1.3	0.138	0.315	1	1951	tags=20%, list=11%, signal=22%
108	LAKE ADULT KIDNEY C29 UNKNOWN NOVEL PT CFH POS SUBPO	78	0.36	1.3	0.101	0.312	1	3486	tags=35%, list=20%, signal=43%
109	CUI_DEVELOPING_HEART_RIGHT_VENTRICULAR_CARDIOMYOCYTE	22	0.45	1.29	0.167	0.32	1	4674	tags=55%, list=26%, signal=74%
110	DESCARTES_MAIN_FETAL_INHIBITORY_INTERNEURONS	26	0.43	1.29	0.153	0.325	1	543	tags=8%, list=3%, signal=8%
111	HE_LIM_SUN_FETAL_LUNG_C3_VENOUS_ENDOTHELIAL_CELL	74	0.36	1.29	0.118	0.324	1	2828	tags=28%, list=16%, signal=34%
112	BUSSLINGER_DUODENAL_K_CELLS	19	0.47	1.28	0.18	0.331	1	1269	tags=21%, list=7%, signal=23%
113	LAKE_ADULT_KIDNEY_C2_PODOCYTES	196	0.3	1.28	0.066	0.33	1	3486	tags=32%, list=20%, signal=39%
114	GAO_LARGE_INTESTINE_24W_C1_DCLK1POS_PROGENITOR	91	0.34	1.28	0.1	0.329	1	3532	tags=42%, list=20%, signal=52%
115	AIZARANI_LIVER_C17_HEPATOCYTES_3	90	0.35	1.28	0.103	0.327	1	3633	tags=36%, list=20%, signal=44%
116	DESCARTES_FETAL_LUNG_MESOTHELIAL_CELLS	81	0.36	1.27	0.128	0.336	1	3095	tags=31%, list=17%, signal=37%
117	BUSSLINGER_GASTRIC_PARIETAL_CELLS	236	0.3	1.27	0.028	0.335	1	1534	tags=12%, list=9%, signal=13%
118	LAKE_ADULT_KIDNEY_C4_PROXIMAL_TUBULE_EPITHELIAL_CELLS_S	159	0.31	1.27	0.077	0.336	1	3800	tags=35%, list=21%, signal=44%
119	LAKE_ADULT_KIDNEY_C15_CONNECTING_TUBULE	159	0.31	1.27	0.068	0.335	1	2860	tags=25%, list=16%, signal=29%
120	HE_LIM_SUN_FETAL_LUNG_C0_MESENCHYMAL_3_CELL	156	0.31	1.26	0.068	0.334	1	2771	tags=30%, list=16%, signal=35%
121	DESCARTES_FETAL_SPLEEN_VASCULAR_ENDOTHELIAL_CELLS	42	0.39	1.26	0.16	0.337	1	4466	tags=40%, list=25%, signal=54%
122	RUBENSTEIN_SKELETAL_MUSCLE_FAP_CELLS	169	0.3	1.26	0.065	0.335	1	3907	tags=38%, list=22%, signal=49%
123	MANNO_MIDBRAIN_NEUROTYPES_HDA2	468	0.27	1.25	0.038	0.344	1	3662	tags=29%, list=21%, signal=35%
124	DESCARTES_MAIN_FETAL_ISLET_ENDOCRINE_CELLS	54	0.37	1.25	0.169	0.346	1	3691	tags=41%, list=21%, signal=51%
125	GAO_LARGE_INTESTINE_24W_C5_LGR5POS_STEM_CELL	54	0.37	1.25	0.144	0.349	1	1593	tags=19%, list=9%, signal=20%
126	DESCARTES_FETAL_PANCREAS_CCL19_CCL21_POSITIVE_CELLS	44	0.37	1.25	0.182	0.347	1	1080	tags=23%, list=6%, signal=24%
127	HE_LIM_SUN_FETAL_LUNG_C1_INTERM_NEUROENDOCRINE_CELL	249	0.29	1.24	0.053	0.356	1	1585	tags=15%, list=9%, signal=17%
128	HU_FETAL_RETINA_AMACRINE	58	0.35	1.24	0.14	0.359	1	4292	tags=45%, list=24%, signal=59%
129	DURANTE_ADULT_OLFACTORY_NEUROEPITHELIUM_PERICYTES	71	0.36	1.23	0.172	0.361	1	1891	tags=21%, list=11%, signal=24%
130	DESCARTES_FETAL_KIDNEY_VASCULAR_ENDOTHELIAL_CELLS	77	0.34	1.23	0.141	0.361	1	5006	tags=47%, list=28%, signal=65%
131	ZHENG_CORD_BLOOD_C7_PUTATIVE_LYMPHOID_PRIMED_MULTIPOT	77	0.34	1.22	0.137	0.373	1	928	tags=13%, list=5%, signal=14%
132	AIZARANI_LIVER_C18_NK_NKT_CELLS_5	102	0.33	1.22	0.109	0.372	1	1422	tags=25%, list=8%, signal=26%
133	DURANTE_ADULT_OLFACTORY_NEUROEPITHELIUM_UNSPECIFIED	30	0.4	1.22	0.189	0.37	1	1173	tags=20%, list=7%, signal=21%
134	GAO_SMALL_INTESTINE_24W_C6_GOBLET_CELLS	33	0.4	1.22	0.163	0.375	1	1833	tags=30%, list=10%, signal=34%
135	DESCARTES_FETAL_INTESTINE_VASCULAR_ENDOTHELIAL_CELLS	39	0.38	1.22	0.203	0.373	1	1699	tags=23%, list=10%, signal=25%
136	HE LIM SUN FETAL LUNG C7 TM4SF4 POS CHODL POS NEURON	53	0.35	1.22	0.186	0.371	1	2617	tags=36%, list=15%, signal=42%
137	ZHENG CORD BLOOD C3 MEGAKARYOCYTE ERYTHROID PROGEN	96	0.32	1.22	0.164	0.37	1	2630	tags=28%, list=15%, signal=33%
138	LAKE ADULT KIDNEY C22 ENDOTHELIAL CELLS GLOMERULAR CAI	131	0.31	1.22	0.126	0.37	1	3896	tags=34%, list=22%, signal=44%
139	DESCARTES_FETAL_LIVER_HEMATOPOIETIC_STEM_CELLS	22	0.42	1.21	0.178	0.373	1	6015	tags=59%, list=34%, signal=89%
140	MANNO_MIDBRAIN_NEUROTYPES_HDA	453	0.26	1.21	0.016	0.381	1	3832	tags=28%, list=22%, signal=35%
141	DESCARTES_MAIN_FETAL_PHOTORECEPTOR_CELLS	62	0.35	1.2	0.21	0.385	1	3791	tags=29%, list=21%, signal=37%
142	AIZARANI_LIVER_C31_KUPFFER_CELLS_5	107	0.32	1.2	0.172	0.393	1	2727	tags=31%, list=15%, signal=36%
143	HE_LIM_SUN_FETAL_LUNG_C3_DEFINITIVE_ERYTHROCYTE	20	0.42	1.19	0.212	0.394	1	735	tags=20%, list=4%, signal=21%
144	HU_FETAL_RETINA_FIBROBLAST	353	0.27	1.19	0.063	0.394	1	1910	tags=19%, list=11%, signal=21%
145	CUI DEVELOPING HEART COMPACT VENTRICULAR CARDIOMYOCY	55	0.34	1.19	0.206	0.392	1	3907	tags=47%, list=22%, signal=60%
146	DESCARTES_FETAL_SPLEEN_STROMAL_CELLS	29	0.39	1.19	0.21	0.396	1	4542	tags=38%, list=26%, signal=51%
147	MANNO_MIDBRAIN_NEUROTYPES_HOMTN	351	0.26	1.19	0.032	0.394	1	4258	tags=31%, list=24%, signal=40%
148	DESCARTES_FETAL_EYE_LENS_FIBRE_CELLS	57	0.34	1.19	0.202	0.395	1	4291	tags=33%, list=24%, signal=44%
149	GAUTAM_EYE_IRIS_CILIARY_BODY_MGP_HIGH_FIBROBLASTS	81	0.32	1.18	0.173	0.4	1	3845	tags=36%, list=22%, signal=45%
150	DESCARTES_FETAL_KIDNEY_ERYTHROBLASTS	129	0.29	1.18	0.158	0.406	1	735	tags=9%, list=4%, signal=10%
151	DESCARTES_FETAL_HEART_SCHWANN_CELLS	51	0.36	1.17	0.228	0.411	1	1113	tags=14%, list=6%, signal=15%
152	HE_LIM_SUN_FETAL_LUNG_C1_MUC5AC_POS_ASCL1_POS_PROGEN	50	0.34	1.17	0.222	0.413	1	609	tags=6%, list=3%, signal=6%
153	ZHONG_PFC_MAJOR_TYPES_OPC	88	0.31	1.16	0.193	0.423	1	3677	tags=34%, list=21%, signal=43%
154	HE_LIM_SUN_FETAL_LUNG_C3_GRIA2_POS_ARTERIAL_ENDO_CELL	109	0.31	1.16	0.177	0.422	1	1827	tags=21%, list=10%, signal=23%
155	MANNO_MIDBRAIN_NEUROTYPES_HPROGM	285	0.27	1.16	0.123	0.426	1	2140	tags=19%, list=12%, signal=22%
156	DESCARTES_FETAL_CEREBELLUM_OLIGODENDROCYTES	64	0.34	1.16	0.237	0.429	1	3677	tags=34%, list=21%, signal=43%
157	MENON_FETAL_KIDNEY_9_ENDOTHELIAL_CELLS	89	0.31	1.16	0.225	0.428	1	1813	tags=21%, list=10%, signal=24%
158	DESCARTES_FETAL_PLACENTA_PAEP_MECOM_POSITIVE_CELLS	129	0.29	1.16	0.17	0.425	1	2084	tags=20%, list=12%, signal=23%
159	GAO_LARGE_INTESTINE_24W_C10_ENTEROCYTE	52	0.34	1.15	0.24	0.427	1	1857	tags=19%, list=10%, signal=21%
160	DESCARTES_FETAL_LUNG_CILIATED_EPITHELIAL_CELLS	368	0.25	1.15	0.075	0.426	1	3266	tags=18%, list=18%, signal=22%
161	LAKE_ADULT_KIDNEY_C27_VASCULAR_SMOOTH_MUSCLE_CELLS_AI	147	0.29	1.15	0.187	0.426	1	3729	tags=40%, list=21%, signal=50%
162	MANNO_MIDBRAIN_NEUROTYPES_HRGL1	336	0.25	1.15	0.078	0.431	1	2745	tags=22%, list=15%, signal=25%
163	AIZARANI_LIVER_C14_HEPATOCYTES_2	196	0.27	1.15	0.146	0.43	1	3633	tags=32%, list=20%, signal=40%
164	TRAVAGLINI_LUNG_NEUROENDOCRINE_CELL	115	0.3	1.14	0.187	0.442	1	3049	tags=25%, list=17%, signal=30%
165	GAO_LARGE_INTESTINE_24W_C8_GOBLET_CELL	33	0.38	1.14	0.261	0.442	1	1639	tags=33%, list=9%, signal=37%
166	HE_LIM_SUN_FETAL_LUNG_C0_AIRWAY_FIBROBLAST	25	0.39	1.14	0.3	0.443	1	1955	tags=24%, list=11%, signal=27%
167	TRAVAGLINI_LUNG_VASCULAR_SMOOTH_MUSCLE_CELL	55	0.33	1.14	0.272	0.441	1	1881	tags=33%, list=11%, signal=36%
168	DESCARTES_FETAL_HEART_LYMPHATIC_ENDOTHELIAL_CELLS	29	0.39	1.14	0.262	0.442	1	1946	tags=17%, list=11%, signal=19%
169	MENON_FETAL_KIDNEY_4_PODOCYTES	187	0.27	1.13	0.178	0.444	1	2169	tags=24%, list=12%, signal=27%
170	HU_FETAL_RETINA_BLOOD	238	0.26	1.13	0.2	0.454	1	1275	tags=13%, list=7%, signal=13%
171	DESCARTES_FETAL_STOMACH_MESOTHELIAL_CELLS	29	0.38	1.12	0.267	0.468	1	3420	tags=45%, list=19%, signal=55%
172	BUSSLINGER_DUODENAL_LATE_IMMATURE_ENTEROCYTES	165	0.27	1.12	0.204	0.466	1	1501	tags=18%, list=8%, signal=20%
173	DESCARTES_FETAL_HEART_VASCULAR_ENDOTHELIAL_CELLS	16	0.42	1.11	0.322	0.477	1	4583	tags=44%, list=26%, signal=59%
174	HE_LIM_SUN_FETAL_LUNG_C6_MUC16_POS_CILIATED_CELL	55	0.33	1.11	0.279	0.483	1	2659	tags=27%, list=15%, signal=32%
175	DESCARTES_FETAL_HEART_STROMAL_CELLS	32	0.35	1.11	0.272	0.483	1	5539	tags=63%, list=31%, signal=91%
176	CUI_DEVELOPING_HEART_C5_VALVAR_CELL	181	0.27	1.11	0.205	0.482	1	2901	tags=27%, list=16%, signal=32%
177	FAN_EMBRYONIC_CTX_NSC_1	15	0.42	1.11	0.318	0.48	1	5431	tags=67%, list=31%, signal=96%
178	DESCARTES_MAIN_FETAL_EXTRAVILLOUS_TROPHOBLASTS	95	0.3	1.11	0.301	0.479	1	2125	tags=19%, list=12%, signal=21%
179	DESCARTES_FETAL_ADRENAL_SLC26A4_PAEP_POSITIVE_CELLS	35	0.36	1.1	0.299	0.49	1	4145	tags=37%, list=23%, signal=48%
180	BUSSLINGER_GASTRIC_G_CELLS	93	0.3	1.1	0.279	0.489	1	3819	tags=37%, list=21%, signal=46%
181	DESCARTES_FETAL_MUSCLE_MYELOID_CELLS	107	0.29	1.1	0.277	0.487	1	2614	tags=24%, list=15%, signal=28%
182	AIZARANI_LIVER_C2_KUPFFER_CELLS_1	176	0.27	1.1	0.218	0.487	1	1584	tags=18%, list=9%, signal=19%
183	LAKE ADULT KIDNEY C16 COLLECTING SYSTEM PRINCIPAL CELLS	130	0.28	1.1	0.246	0.487	1	2860	tags=25%, list=16%, signal=30%
184	AIZARANI_LIVER_C20_LSECS_3	275	0.25	1.1	0.213	0.486	1	2361	tags=21%, list=13%, signal=24%
185	DESCARTES_MAIN_FETAL_SYMPATHOBLASTS	44	0.34	1.1	0.319	0.488	1	3996	tags=41%, list=22%, signal=53%
186	TRAVAGLINI_LUNG_MYOFIBROBLAST_CELL	61	0.32	1.09	0.275	0.49	1	1803	tags=20%, list=10%, signal=22%
187	HE_LIM_SUN_FETAL_LUNG_C3_LYMPHATIC_ENDOTHELIAL_CELL	352	0.24	1.09	0.192	0.487	1	3499	tags=25%, list=20%, signal=31%
188	MANNO_MIDBRAIN_NEUROTYPES_HRN	303	0.25	1.09	0.216	0.488	1	3882	tags=30%, list=22%, signal=37%

189	DESCARTES_MAIN_FETAL_PAEP_MECOM_POSITIVE_CELLS	103	0.29	1.09	0.25	0.487	1	3652 tags=32%, list=21%, signal=40%
190	DESCARTES_MAIN_FETAL_INTESTINAL_EPITHELIAL_CELLS	67	0.31	1.09	0.304	0.489	1	2156 tags=21%, list=12%, signal=24%
191	MENON_FETAL_KIDNEY_2_NEPHRON_PROGENITOR_CELLS	36	0.33	1.09	0.316	0.487	1	2190 tags=31%, list=12%, signal=35%
192	AIZARANI_LIVER_C4_EPCAM_POS_BILE_DUCT_CELLS_1	183	0.26	1.09	0.283	0.485	1	1601 tags=17%, list=9%, signal=18%
193	HE_LIM_SUN_FETAL_LUNG_C0_ASPN_POS_CHONDROCYTE	112	0.28	1.09	0.267	0.488	1	2512 tags=19%, list=14%, signal=22%
194	TRAVAGLINI_LUNG_MACROPHAGE_CELL	185	0.26	1.09	0.298	0.486	1	1890 tags=22%, list=11%, signal=24%
195	VANGURP_PANCREATIC_ALPHA_CELL	35	0.34	1.08	0.299	0.493	1	1987 tags=29%, list=11%, signal=32%
196	DESCARTES_FETAL_HEART_CLC_IL5RA_POSITIVE_CELLS	17	0.4	1.08	0.333	0.494	1	2727 tags=41%, list=15%, signal=49%
197	DURANTE_ADULT_OLFACTORY_NEUROEPITHELIUM_MACROPHAGES	69	0.31	1.08	0.326	0.492	1	1064 tags=19%, list=6%, signal=20%
198	TRAVAGLINI_LUNG_ALVEOLAR_FIBROBLAST_CELL	158	0.26	1.07	0.263	0.508	1	3357 tags=33%, list=19%, signal=40%
199	TRAVAGLINI_LUNG_AIRWAY_SMOOTH_MUSCLE_CELL	148	0.26	1.07	0.29	0.507	1	2959 tags=28%, list=17%, signal=34%
200	HAY_BONE_MARROW_CD34_POS_EO_B_MAST	15	0.41	1.07	0.344	0.505	1	2648 tags=33%, list=15%, signal=39%
201	MANNO_MIDBRAIN_NEUROTYPES_HNBM	254	0.25	1.07	0.256	0.503	1	2508 tags=20%, list=14%, signal=23%
202	DESCARTES_FETAL_HEART_MYELOID_CELLS	104	0.28	1.07	0.34	0.506	1	1422 tags=18%, list=8%, signal=20%
203	LAKE_ADULT_KIDNEY_C13_THICK_ASCENDING_LIMB	118	0.28	1.07	0.322	0.506	1	3339 tags=24%, list=19%, signal=29%
204	TRAVAGLINI_LUNG_SEROUS_CELL	77	0.3	1.07	0.36	0.504	1	1146 tags=17%, list=6%, signal=18%
205	DESCARTES_MAIN_FETAL_ERYTHROBLASTS	195	0.25	1.07	0.273	0.506	1	898 tags=9%, list=5%, signal=10%
206	AIZARANI_LIVER_C39_EPCAM_POS_BILE_DUCT_CELLS_4	172	0.27	1.07	0.28	0.505	1	1601 tags=19%, list=9%, signal=20%
207	HE_LIM_SUN_FETAL_LUNG_C7_MID_SCHWANN_CELL	253	0.24	1.06	0.274	0.511	1	2483 tags=23%, list=14%, signal=26%
208	MANNO_MIDBRAIN_NEUROTYPES_HOPC	337	0.24	1.06	0.29	0.52	1	3060 tags=23%, list=17%, signal=27%
209	LAKE_ADULT_KIDNEY_C25_ENDOTHELIAL_CELLS_UNASSIGNED	35	0.34	1.06	0.34	0.52	1	3973 tags=34%, list=22%, signal=44%
210	DESCARTES_FETAL_MUSCLE_SATELLITE_CELLS	39	0.33	1.06	0.328	0.519	1	4210 tags=31%, list=24%, signal=40%
211	DESCARTES_MAIN_FETAL_OLIGODENDROCYTES	40	0.33	1.05	0.34	0.526	1	5423 tags=45%, list=31%, signal=65%
212	GAO_LARGE_INTESTINE_ADULT_CA_ENTEROENDOCRINE_CELLS	269	0.25	1.05	0.301	0.532	1	3094 tags=20%, list=17%, signal=24%
213	DESCARTES_FETAL_CEREBRUM_OLIGODENDROCYTES	47	0.31	1.05	0.379	0.532	1	1887 tags=23%, list=11%, signal=26%
214	DESCARTES_MAIN_FETAL_METANEPHRIC_CELLS	27	0.36	1.05	0.323	0.531	1	4295 tags=37%, list=24%, signal=49%
215	DESCARTES_FETAL_LIVER_HEPATOBLASTS	372	0.23	1.05	0.268	0.529	1	2209 tags=19%, list=12%, signal=21%
216	AIZARANI_LIVER_C6_KUPFFER_CELLS_2	201	0.25	1.04	0.336	0.529	1	1422 tags=17%, list=8%, signal=18%
217	HE_LIM_SUN_FETAL_LUNG_C2_SPP1_POS_MACROPHAGE_CELL	45	0.32	1.04	0.369	0.529	1	2634 tags=24%, list=15%, signal=29%
218	HE_LIM_SUN_FETAL_LUNG_C3_DEFINITIVE_RETICULOCYTE	101	0.28	1.04	0.335	0.53	1	2030 tags=19%, list=11%, signal=21%
219	HAY_BONE_MARROW_CD34_POS_PRE_PC	99	0.27	1.04	0.371	0.53	1	2719 tags=27%, list=15%, signal=32%
220	AIZARANI_LIVER_C11_HEPATOCYTES_1	259	0.24	1.04	0.36	0.536	1	3084 tags=26%, list=17%, signal=31%
221	DESCARTES_MAIN_FETAL_PDE11A_FAM19A2_POSITIVE_CELLS	40	0.32	1.04	0.358	0.536	1	4602 tags=45%, list=26%, signal=61%
222	MENON_FETAL_KIDNEY_8_CONNECTING_TUBULE_CELLS	244	0.24	1.04	0.369	0.534	1	2796 tags=22%, list=16%, signal=26%
223	TRAVAGLINI_LUNG_LYMPHATIC_CELL	193	0.25	1.03	0.349	0.537	1	2981 tags=25%, list=17%, signal=30%
224	DESCARTES_FETAL_INTESTINE_ERYTHROBLASTS	132	0.27	1.03	0.384	0.535	1	735 tags=9%, list=4%, signal=9%
225	DESCARTES_FETAL_MUSCLE_STROMAL_CELLS	74	0.29	1.03	0.402	0.539	1	4079 tags=42%, list=23%, signal=54%
226	HU_FETAL_RETINA_RPE	269	0.24	1.03	0.36	0.54	1	2158 tags=19%, list=12%, signal=22%
227	HU_FETAL_RETINA_PHOTORECEPTOR	65	0.28	1.03	0.382	0.537	1	3211 tags=28%, list=18%, signal=34%
228	DURANTE_ADULT_OLFACTORY_NEUROEPITHELIUM_FIBROBLASTS_S	80	0.28	1.03	0.348	0.537	1	4244 tags=43%, list=24%, signal=56%
229	MANNO_MIDBRAIN_NEUROTYPES_HNBML1	289	0.24	1.03	0.37	0.534	1	2508 tags=19%, list=14%, signal=21%
230	MURARO_PANCREAS_ENDOTHELIAL_CELL	338	0.23	1.03	0.363	0.539	1	3246 tags=26%, list=18%, signal=32%
231	DESCARTES_FETAL_ADRENAL_MEGAKARYOCYTES	84	0.27	1.02	0.407	0.561	1	3995 tags=35%, list=22%, signal=44%
232	BUSSLINGER_DUODENAL_TUFT_CELLS	71	0.28	1.02	0.419	0.562	1	2498 tags=27%, list=14%, signal=31%
233	RUBENSTEIN_SKELETAL_MUSCLE_FBN1_FAP_CELLS	266	0.24	1.01	0.384	0.563	1	2121 tags=18%, list=12%, signal=21%
234	HAY_BONE_MARROW_CD34_POS_MKP	60	0.29	1.01	0.399	0.563	1	1881 tags=23%, list=11%, signal=26%
235	DESCARTES_FETAL_KIDNEY_METANEPHRIC_CELLS	107	0.27	1.01	0.376	0.561	1	2149 tags=21%, list=12%, signal=23%
236	BUSSLINGER_GASTRIC_X_CELLS	182	0.25	1.01	0.375	0.56	1	1945 tags=17%, list=11%, signal=19%
237	DESCARTES_MAIN_FETAL_MYELOID_CELLS	70	0.29	1.01	0.419	0.565	1	2939 tags=27%, list=17%, signal=32%
238	VANGURP_PANCREATIC_DELTA_CELL	70	0.27	1.01	0.44	0.568	1	2950 tags=26%, list=17%, signal=31%
239	DESCARTES_FETAL_CEREBELLUM_GRANULE_NEURONS	24	0.35	1	0.393	0.573	1	2625 tags=17%, list=15%, signal=20%
240	AIZARANI_LIVER_C21_STELLATE_CELLS_1	186	0.24	1	0.447	0.576	1	3921 tags=31%, list=22%, signal=40%
241	DESCARTES_FETAL_LUNG_MYELOID_CELLS	127	0.26	1	0.401	0.587	1	2614 tags=25%, list=15%, signal=29%
242	HU_FETAL_RETINA_RPC	97	0.26	0.98	0.464	0.618	1	1848 tags=14%, list=10%, signal=16%
243	DESCARTES_FETAL_LIVER_MESOTHELIAL_CELLS	267	0.23	0.98	0.479	0.62	1	3568 tags=27%, list=20%, signal=34%
244	GAO_LARGE_INTESTINE_24W_C2_MKI67POS_PROGENITOR	109	0.26	0.98	0.461	0.618	1	1546 tags=16%, list=9%, signal=17%
245	MANNO_MIDBRAIN_NEUROTYPES_HRGL2B	402	0.21	0.98	0.579	0.632	1	3060 tags=22%, list=17%, signal=26%
246	HE_LIM_SUN_FETAL_LUNG_C0_RESTING_CHONDROCYTE	104	0.25	0.98	0.485	0.629	1	2739 tags=21%, list=15%, signal=25%
247	GAO_LARGE_INTESTINE_ADULT_CG_GOBLET_CELL_SUBTYPE_2	38	0.3	0.98	0.429	0.627	1	2512 tags=26%, list=14%, signal=31%
248	FAN_EMBRYONIC_CTX_BRAIN_ENDOTHELIAL_2	291	0.22	0.98	0.557	0.625	1	1972 tags=19%, list=11%, signal=21%
249	GAO_LARGE_INTESTINE_ADULT_CE_OLFM4HIGH_STEM_CELL	272	0.23	0.97	0.503	0.626	1	2940 tags=21%, list=17%, signal=25%
250	HE_LIM_SUN_FETAL_LUNG_C0_MYOFIBROBLAST_1_CELL	56	0.28	0.97	0.444	0.629	1	3091 tags=25%, list=17%, signal=30%
251	LAKE_ADULT_KIDNEY_C3_PROXIMAL_TUBULE_EPITHELIAL_CELLS_S	197	0.23	0.97	0.5	0.627	1	3782 tags=28%, list=21%, signal=36%
252	FAN_OVARY_CL11_MURAL_GRANULOSA_CELL	398	0.21	0.96	0.581	0.645	1	2190 tags=18%, list=12%, signal=20%
253	DESCARTES_FETAL_MUSCLE_ERYTHROBLASTS	103	0.25	0.96	0.508	0.649	1	1094 tags=9%, list=6%, signal=9%
254	HE_LIM_SUN_FETAL_LUNG_C1_PULMONARY_NEUROENDOCRINE_CE	215	0.23	0.96	0.577	0.66	1	4773 tags=37%, list=27%, signal=50%
255	HE_LIM_SUN_FETAL_LUNG_C5_LATE_PRO_B_CELL	22	0.33	0.95	0.475	0.663	1	2461 tags=36%, list=14%, signal=42%
256	HE_LIM_SUN_FETAL_LUNG_C2_DC2_CELL	48	0.29	0.95	0.452	0.662	1	1658 tags=17%, list=9%, signal=18%
257	DESCARTES_FETAL_LUNG_MEGAKARYOCYTES	135	0.24	0.95	0.582	0.666	1	3748 tags=33%, list=21%, signal=41%
258	DESCARTES_MAIN_FETAL_LENS_FIBRE_CELLS	135	0.23	0.94	0.586	0.693	1	4291 tags=32%, list=24%, signal=42%
259	HAY_BONE_MARROW_EOSINOPHIL	46	0.28	0.94	0.518	0.693	1	206 tags=7%, list=1%, signal=7%
260	DURANTE_ADULT_OLFACTORY_NEUROEPITHELIUM_IMMATURE_NEU	132	0.24	0.94	0.58	0.694	1	4340 tags=33%, list=24%, signal=44%
261	DESCARTES_FETAL_MUSCLE_VASCULAR_ENDOTHELIAL_CELLS	59	0.27	0.94	0.504	0.692	1	1699 tags=20%, list=10%, signal=22%
262	CUI_DEVELOPING_HEART_C2_CARDIOMYOCYTE	85	0.26	0.94	0.55	0.693	1	2988 tags=25%, list=17%, signal=30%
263	DESCARTES_FETAL_PLACENTA_VASCULAR_ENDOTHELIAL_CELLS	85	0.25	0.94	0.561	0.691	1	4400 tags=38%, list=25%, signal=50%
264	DESCARTES_FETAL_PANCREAS_MESOTHELIAL_CELLS	62	0.27	0.93	0.53	0.698	1	3034 tags=29%, list=17%, signal=35%
265	MURARO_PANCREAS_DELTA_CELL	231	0.22	0.93	0.687	0.698	1	3719 tags=26%, list=21%, signal=33%
266	DESCARTES_MAIN_FETAL_RETINAL_PROGENITORS_AND_MULLER_G	45	0.28	0.92	0.56	0.737	1	3444 tags=31%, list=19%, signal=38%
267	DESCARTES_FETAL_PLACENTA_SMOOTH_MUSCLE_CELLS	71	0.26	0.91	0.566	0.738	1	3773 tags=30%, list=21%, signal=37%
268	DESCARTES_MAIN_FETAL_MESANGIAL_CELLS	15	0.34	0.91	0.536	0.739	1	1617 tags=20%, list=9%, signal=22%
269	BUSSLINGER_ESOPHAGEAL_EARLY_SUPRABASAL_CELLS	65	0.26	0.91	0.597	0.736	1	1453 tags=17%, list=8%, signal=18%
270	DESCARTES_FETAL_EYE_MICROGLIA	135	0.23	0.91	0.644	0.735	1	2896 tags=21%, list=16%, signal=25%
271	HE_LIM_SUN_FETAL_LUNG_C7_COL20A1_POS_SCHWANN_CELL	68	0.25	0.91	0.594	0.736	1	3224 tags=26%, list=18%, signal=32%
272	DESCARTES_FETAL_PANCREAS_DUCTAL_CELLS	97	0.24	0.9	0.651	0.752	1	1733 tags=14%, list=10%, signal=16%
273	DESCARTES_MAIN_FETAL_SCHWANN_CELLS	36	0.29	0.89	0.54	0.772	1	4293 tags=33%, list=24%, signal=44%
274	DESCARTES_FETAL_LUNG_STROMAL_CELLS	40	0.28	0.89	0.549	0.772	1	3619 tags=33%, list=20%, signal=41%
275	ZHONG_PFC_C7_ORG_UNDERGOING_NEURONAL_DIFFERENTIATION	57	0.25	0.89	0.626	0.77	1	2484 tags=19%, list=14%, signal=22%
276	TRAVAGLINI_LUNG_PERICYTE_CELL	110	0.24	0.89	0.668	0.779	1	1907 tags=16%, list=11%, signal=18%
277	DESCARTES_MAIN_FETAL_RETINAL_PIGMENT_CELLS	107	0.24	0.89	0.674	0.778	1	3806 tags=30%, list=21%, signal=38%
278	ZHENG_CORD_BLOOD_C10_MULTILYMPHOID_PROGENITOR	85	0.24	0.87	0.721	0.809	1	3935 tags=29%, list=22%, signal=38%
279	DESCARTES_FETAL_LIVER_STELLATE_CELLS	127	0.22	0.87	0.686	0.808	1	3214 tags=25%, list=18%, signal=31%
280	MANNO_MIDBRAIN_NEUROTYPES_HNBML5	418	0.19	0.87	0.954	0.808	1	3725 tags=23%, list=21%, signal=29%
281	HAY_BONE_MARROW_CD34_POS_HSC	95	0.23	0.86	0.707	0.84	1	3161 tags=26%, list=18%, signal=32%
282	ZHONG_PFC_C8_UNKNOWN_NEUROD2_POS_INTERNEURON	79	0.24	0.86	0.695	0.842	1	2479 tags=20%, list=14%, signal=23%
283	HE_LIM_SUN_FETAL_LUNG_C0_VASCULAR_SMC_1_CELL	112	0.22	0.85	0.795	0.846	1	1972 tags=17%, list=11%, signal=19%
284	HE_LIM_SUN_FETAL_LUNG_C3_AEROCYTE	82	0.24	0.85	0.716	0.845	1	2954 tags=23%, list=17%, signal=28%

285	FAN_EMBRYONIC_CTX_ASTROCYTE_2	141	0.22	0.85	0.798	0.843	1	2639	tags=21%, list=15%, signal=24%
286	DESCARTES_MAIN_FETAL_STELLATE_CELLS	42	0.25	0.84	0.735	0.854	1	4039	tags=29%, list=23%, signal=37%
287	DESCARTES_FETAL_PLACENTA_MEGAKARYOCYTES	160	0.21	0.84	0.852	0.857	1	2905	tags=19%, list=16%, signal=23%
288	DESCARTES_MAIN_FETAL_VISCERAL_NEURONS	42	0.26	0.84	0.695	0.857	1	1703	tags=19%, list=10%, signal=21%
289	FAN_EMBRYONIC_CTX_EX_4_EXCITATORY_NEURON	131	0.21	0.84	0.833	0.856	1	4373	tags=33%, list=25%, signal=43%
290	GAUTAM_EYE_IRIS_CILIARY_BODY_MONOCYTES	108	0.22	0.84	0.815	0.859	1	2354	tags=16%, list=13%, signal=18%
291	HE LIM SUN FETAL LUNG C7 TM4SF4 POS PENK POS NEURON (	113	0.22	0.84	0.814	0.859	1	4563	tags=39%, list=26%, signal=52%
292	DESCARTES_MAIN_FETAL_ENDOCARDIAL_CELLS	72	0.23	0.83	0.757	0.863	1	4366	tags=38%, list=25%, signal=50%
293	DESCARTES_FETAL_PANCREAS_VASCULAR_ENDOTHELIAL_CELLS	53	0.24	0.83	0.747	0.865	1	1699	tags=17%, list=10%, signal=19%
294	DESCARTES_FETAL_PLACENTA_STROMAL_CELLS	58	0.24	0.83	0.705	0.864	1	2301	tags=17%, list=13%, signal=20%
295	DESCARTES_FETAL_PANCREAS_LYMPHATIC_ENDOTHELIAL_CELLS	41	0.25	0.83	0.74	0.865	1	1846	tags=17%, list=10%, signal=19%
296	HE_LIM_SUN_FETAL_LUNG_C1_AT1_CELL	39	0.26	0.82	0.715	0.865	1	3196	tags=26%, list=18%, signal=31%
297	DESCARTES_MAIN_FETAL_LYMPHATIC_ENDOTHELIAL_CELLS	65	0.23	0.82	0.766	0.867	1	3471	tags=29%, list=20%, signal=36%
298	DESCARTES_MAIN_FETAL_HEPATOBLASTS	73	0.23	0.81	0.773	0.878	1	3350	tags=22%, list=19%, signal=27%
299	HE_LIM_SUN_FETAL_LUNG_C0_MYL4_POS_SMC_CELL	109	0.21	0.81	0.856	0.881	1	2742	tags=20%, list=15%, signal=24%
300	HE_LIM_SUN_FETAL_LUNG_C7_SST_POS_NEURON_CELL	349	0.18	0.81	0.981	0.879	1	5440	tags=40%, list=31%, signal=56%
301	DESCARTES_FETAL_KIDNEY_MESANGIAL_CELLS	46	0.24	0.81	0.761	0.879	1	4120	tags=26%, list=23%, signal=34%
302	ZHENG CORD BLOOD C2 PUTATIVE BASOPHIL EOSINOPHIL MAST	90	0.22	0.8	0.868	0.897	1	2667	tags=20%, list=15%, signal=23%
303	DESCARTES_FETAL_EYE_BIPOLAR_CELLS	20	0.28	0.79	0.735	0.9	1	12845	tags=100%, list=72%, signal=361%
304	HE_LIM_SUN_FETAL_LUNG_C0_MYOFIBROBLAST_3_CELL	25	0.27	0.79	0.748	0.908	1	362	tags=8%, list=2%, signal=8%
305	DESCARTES_FETAL_LUNG_LYMPHATIC_ENDOTHELIAL_CELLS	51	0.23	0.78	0.791	0.911	1	4466	tags=39%, list=25%, signal=52%
306	CUI_DEVELOPING_HEART_RIGHT_ATRIAL_CARDIOMYOCYTE	36	0.25	0.78	0.812	0.912	1	3075	tags=28%, list=17%, signal=34%
307	HE_LIM_SUN_FETAL_LUNG_C7_KCNIP4_POS_NEURON_CELL	102	0.21	0.78	0.872	0.912	1	3434	tags=25%, list=19%, signal=30%
308	GAO_ESOPHAGUS_25W_C1_CILIATED_EPITHELIAL_CELLS	389	0.17	0.77	1	0.916	1	3349	tags=19%, list=19%, signal=23%
309	DESCARTES_FETAL_LIVER_VASCULAR_ENDOTHELIAL_CELLS	108	0.2	0.77	0.94	0.919	1	1673	tags=14%, list=9%, signal=15%
310	DESCARTES_MAIN_FETAL_AMACRINE_CELLS	18	0.27	0.77	0.773	0.917	1	4169	tags=39%, list=23%, signal=51%
311	DESCARTES_FETAL_STOMACH_CILIATED_EPITHELIAL_CELLS	240	0.18	0.77	0.963	0.916	1	4866	tags=28%, list=27%, signal=38%
312	HAY_BONE_MARROW_CD34_POS_MULTILIN	19	0.27	0.76	0.76	0.922	1	3115	tags=32%, list=18%, signal=38%
313	LAKE_ADULT_KIDNEY_C14_DISTAL_CONVOLUTED_TUBULE	187	0.18	0.76	0.963	0.921	1	2024	tags=13%, list=11%, signal=15%
314	DESCARTES_MAIN_FETAL_GANGLION_CELLS	19	0.27	0.75	0.79	0.926	1	4643	tags=37%, list=26%, signal=50%
315	CUI_DEVELOPING_HEART_LEFT_ATRIAL_CARDIOMYOCYTE	17	0.28	0.75	0.802	0.928	1	5447	tags=47%, list=31%, signal=68%
316	DESCARTES_FETAL_INTESTINE_LYMPHATIC_ENDOTHELIAL_CELLS	26	0.25	0.74	0.816	0.928	1	4721	tags=42%, list=27%, signal=58%
317	LAKE_ADULT_KIDNEY_C1_EPITHELIAL_CELLS_UNASSIGNED	28	0.24	0.74	0.835	0.935	1	696	tags=11%, list=4%, signal=11%
318	DESCARTES_FETAL_THYMUS_THYMOCYTES	19	0.26	0.72	0.808	0.942	1	5185	tags=42%, list=29%, signal=59%
319	DESCARTES_MAIN_FETAL_GRANULE_NEURONS	16	0.26	0.71	0.829	0.951	1	3056	tags=25%, list=17%, signal=30%
320	HE_LIM_SUN_FETAL_LUNG_C0_MID_AIRWAY_SMC_1_CELL	65	0.2	0.68	0.984	0.969	1	2959	tags=20%, list=17%, signal=24%
321	HU_FETAL_RETINA_HORIZONTAL	33	0.21	0.67	0.924	0.976	1	2775	tags=24%, list=16%, signal=29%
322	DESCARTES_FETAL_CEREBRUM_LIMBIC_SYSTEM_NEURONS	21	0.21	0.61	0.949	0.994	1	1173	tags=14%, list=7%, signal=15%
323	DESCARTES_MAIN_FETAL_CILIATED_EPITHELIAL_CELLS	474	0.13	0.59	1	0.996	1	5389	tags=32%, list=30%, signal=45%
324	DESCARTES_FETAL_CEREBELLUM_INHIBITORY_INTERNEURONS	25	0.18	0.54	0.98	1	1	3646	tags=24%, list=21%, signal=30%
325	DESCARTES_MAIN_FETAL_CARDIOMYOCYTES	26	0.16	0.47	0.997	1	1	4184	tags=27%, list=24%, signal=35%
326	DESCARTES_FETAL_CEREBELLUM_UNIPOLAR_BRUSH_CELLS	21	0.15	0.45	0.997	0.999	1	4890	tags=29%, list=28%, signal=39%

CELL SIGNATURE Gene sets enriched in *Sox2* $^{\Delta/\Delta}$ Forestomachs

	GS	SIZE	ES	NES	NOM p-va	FDR q-val	FWER p-v	RANK AT MAX	LEADING EDGE
1	RUBENSTEIN_SKELETAL_MUSCLE_T_CELLS	137	-0.64	-2.23	0	0	0	3229	tags=51%, list=18%, signal=62%
2	DESCARTES_MAIN_FETAL_SQUAMOUS_EPITHELIAL_CELLS	83	-0.65	-2.14	0	0	0	1335	tags=41%, list=8%, signal=44%
3	DESCARTES_FETAL_LUNG_SQUAMOUS_EPITHELIAL_CELLS	158	-0.57	-2	0	0.001	0.004	2465	tags=44%, list=14%, signal=51%
4	BUSSLINGER_ESOPHAGEAL_LATE_SUPRABASAL_CELLS	126	-0.56	-1.93	0	0.002	0.011	2215	tags=37%, list=12%, signal=41%
5	DESCARTES_MAIN_FETAL_ELF3_AGBL2_POSITIVE_CELLS	130	-0.54	-1.9	0	0.003	0.022	3598	tags=40%, list=20%, signal=50%
6	RUBENSTEIN_SKELETAL_MUSCLE_B_CELLS	139	-0.54	-1.89	0	0.003	0.029	2966	tags=37%, list=17%, signal=45%
7	DESCARTES_MAIN_FETAL_ACINAR_CELLS	50	-0.6	-1.85	0	0.005	0.051	675	tags=38%, list=4%, signal=39%
8	TRAVAGLINI_LUNG_CD4_NAIVE_T_CELL	100	-0.55	-1.84	0	0.006	0.061	3492	tags=50%, list=20%, signal=62%
9	RUBENSTEIN_SKELETAL_MUSCLE_SATELLITE_CELLS	258	-0.49	-1.82	0	0.007	0.086	3293	tags=40%, list=19%, signal=49%
10	DESCARTES_FETAL_PANCREAS_ACINAR_CELLS	61	-0.58	-1.8	0	0.01	0.13	675	tags=30%, list=4%, signal=31%
11	RUBENSTEIN_SKELETAL_MUSCLE_MYELOID_CELLS	291	-0.47	-1.77	0	0.015	0.212	2548	tags=30%, list=14%, signal=34%
12	DESCARTES_FETAL_STOMACH_SQUAMOUS_EPITHELIAL_CELLS	53	-0.57	-1.76	0	0.016	0.24	1027	tags=32%, list=6%, signal=34%
13	TRAVAGLINI_LUNG_DIFFERENTIATING_BASAL_CELL	144	-0.5	-1.75	0	0.018	0.291	1827	tags=33%, list=10%, signal=37%
14	DESCARTES_MAIN_FETAL_URETERIC_BUD_CELLS	26	-0.64	-1.73	0.003	0.021	0.345	651	tags=23%, list=4%, signal=24%
15	ZHONG_PFC_C2_UNKNOWN_NPC	74	-0.53	-1.72	0.001	0.025	0.422	4654	tags=47%, list=26%, signal=64%
16	RUBENSTEIN_SKELETAL_MUSCLE_NK_CELLS	191	-0.47	-1.71	0	0.027	0.474	3293	tags=34%, list=19%, signal=41%
17	DESCARTES MAIN FETAL CORNEAL AND CONJUNCTIVAL EPITHEL	208	-0.47	-1.7	0	0.028	0.507	2480	tags=26%, list=14%, signal=30%
18	DESCARTES_FETAL_MUSCLE_MEGAKARYOCYTES	198	-0.47	-1.7	0	0.027	0.513	4572	tags=41%, list=26%, signal=55%
19	BUSSLINGER_DUODENAL_TRANSIT_AMPLIFYING_CELLS	148	-0.48	-1.69	0	0.028	0.55	3030	tags=42%, list=17%, signal=50%
20	GAUTAM_EYE_IRIS_CILIARY_BODY_WIF1_HIGH_FIBROBLASTS	97	-0.5	-1.68	0.003	0.03	0.593	3476	tags=40%, list=20%, signal=50%
21	HE_LIM_SUN_FETAL_LUNG_C1_SQUAMOUS_CELL	216	-0.45	-1.65	0	0.044	0.734	2963	tags=37%, list=17%, signal=44%
22	FAN_OVARY_CL10_PUTATIVE_EARLY_ATRESIA_GRANULOSA_CELL	241	-0.44	-1.63	0	0.055	0.827	4208	tags=40%, list=24%, signal=52%
23	FAN_OVARY_CL12_T_LYMPHOCYTE_NK_CELL_2	141	-0.46	-1.62	0.001	0.06	0.867	3785	tags=38%, list=21%, signal=47%
24	FAN OVARY CL2 PUTATIVE EARLY ATRETIC FOLLICLE THECAL CE	82	-0.49	-1.62	0.003	0.058	0.871	3200	tags=41%, list=18%, signal=50%
25	GAUTAM_EYE_CORNEA_ELF3_HIGH_EPITHELIAL_CELLS	142	-0.46	-1.61	0.001	0.056	0.877	4851	tags=44%, list=27%, signal=61%
26	TRAVAGLINI_LUNG_BASAL_CELL	163	-0.45	-1.61	0	0.056	0.881	3527	tags=37%, list=20%, signal=46%
27	GAO_ESOPHAGUS_25W_C2_KRT6BPOS_SECRETORY_PROGENITOR_	61	-0.51	-1.6	0.015	0.062	0.92	2221	tags=30%, list=13%, signal=34%
28	HAY_BONE_MARROW_IMMATURE_NEUTROPHIL	172	-0.45	-1.59	0	0.063	0.933	4434	tags=47%, list=25%, signal=61%
29	TRAVAGLINI_LUNG_BRONCHIAL_VESSEL_1_CELL	182	-0.44	-1.59	0.001	0.063	0.939	4136	tags=42%, list=23%, signal=55%
30	AIZARANI_LIVER_C3_NK_NKT_CELLS_2	136	-0.46	-1.59	0.002	0.064	0.942	3955	tags=41%, list=22%, signal=53%
31	AIZARANI_LIVER_C1_NK_NKT_CELLS_1	134	-0.46	-1.59	0	0.063	0.945	4012	tags=38%, list=23%, signal=49%
32	FAN_OVARY_CL0_XBP1_SELK_HIGH_STROMAL_CELL	111	-0.46	-1.58	0.001	0.064	0.954	3200	tags=40%, list=18%, signal=48%
33	HAY_BONE_MARROW_NAIVE_T_CELL	319	-0.42	-1.58	0	0.062	0.956	3893	tags=36%, list=22%, signal=45%
34	DESCARTES_MAIN_FETAL_CCL19_CCL21_POSITIVE_CELLS	162	-0.44	-1.58	0.001	0.063	0.962	3241	tags=33%, list=18%, signal=40%
35	CUI_DEVELOPING_HEART_C9_B_T_CELL	134	-0.45	-1.58	0.004	0.062	0.966	2911	tags=24%, list=16%, signal=28%
36	RUBENSTEIN_SKELETAL_MUSCLE_PCV_ENDOTHELIAL_CELLS	180	-0.44	-1.57	0.001	0.064	0.97	3218	tags=34%, list=18%, signal=42%
37	DURANTE_ADULT_OLFACTORY_NEUROEPITHELIUM_CD8_T_CELLS	65	-0.49	-1.56	0.014	0.068	0.978	2928	tags=32%, list=16%, signal=39%
38	FAN_OVARY_CL4_T_LYMPHOCYTE_NK_CELL_1	219	-0.43	-1.56	0	0.07	0.98	3927	tags=34%, list=22%, signal=43%
39	LAKE_ADULT_KIDNEY_C12_THICK_ASCENDING_LIMB	327	-0.41	-1.55	0	0.073	0.982	3218	tags=31%, list=18%, signal=37%
40	GAUTAM_EYE_IRIS_CILIARY_BODY_FIBROBLASTS	95	-0.46	-1.53	0.003	0.094	0.996	3288	tags=34%, list=19%, signal=41%
41	GAUTAM_EYE_CHOROID_SCLERA_CHOROID_ENDOTHELIAL_CELLS	121	-0.44	-1.52	0.005	0.1	0.998	3143	tags=29%, list=18%, signal=35%
42	FAN_OVARY_CL13_MONOCYTE_MACROPHAGE	403	-0.39	-1.51	0	0.102	0.998	4287	tags=37%, list=24%, signal=47%
43	TRAVAGLINI_LUNG_CLUB_CELL	88	-0.45	-1.51	0.009	0.103	0.998	2940	tags=42%, list=17%, signal=50%
44	ZHONG_PFC_C1_MICROGLIA	236	-0.41	-1.5	0.005	0.107	1	4617	tags=39%, list=26%, signal=51%
45	GAUTAM_EYE_CORNEA_MELANOCYTES	239	-0.41	-1.5	0	0.105	1	4615	tags=40%, list=26%, signal=54%
46	HAY_BONE_MARROW_DENDRITIC_CELL	162	-0.42	-1.5	0.006	0.105	1	3068	tags=27%, list=17%, signal=32%
47	ZHONG_PFC_C1_ASTROCYTE	28	-0.54	-1.5	0.032	0.104	1	3425	tags=43%, list=19%, signal=53%
48	TRAVAGLINI_LUNG_NEUTROPHIL_CELL	303	-0.4	-1.5	0	0.104	1	3966	tags=35%, list=22%, signal=44%
49	HE_LIM_SUN_FETAL_LUNG_C1_PROXIMAL_BASAL_CELL	171	-0.41	-1.49	0.005	0.114	1	2459	tags=26%, list=14%, signal=30%
50	HE_LIM_SUN_FETAL_LUNG_C1_PROXIMAL_SECRETORY_3_CELL	196	-0.41	-1.49	0.004	0.114	1	1932	tags=23%, list=11%, signal=25%
51	HAY_BONE_MARROW_NK_CELLS	315	-0.39	-1.48	0.001	0.112	1	3744	tags=28%, list=21%, signal=34%

52	ZHONG_PFC_C8_ORG_PROLIFERATING	59	-0.47	-1.48	0.026	0.114	1	4654	tags=49%, list=26%, signal=66%
53	HE LIM SUN FETAL LUNG C7 FGFBP2 POS NEURAL PROGENITOR	46	-0.49	-1.47	0.032	0.12	1	2497	tags=26%, list=14%, signal=30%
54	LAKE_ADULT_KIDNEY_C7_PROXIMAL_TUBULE_EPITHELIAL_CELLS_S	105	-0.44	-1.47	0.012	0.12	1	2944	tags=34%, list=17%, signal=41%
55	HE_LIM_SUN_FETAL_LUNG_C1_LATE_BASAL_CELL	96	-0.43	-1.45	0.023	0.146	1	3973	tags=38%, list=22%, signal=48%
56	ZHONG_PFC_C1_OPC	224	-0.39	-1.44	0.005	0.159	1	4617	tags=38%, list=26%, signal=50%
57	MANNO_MIDBRAIN_NEUROTYPES_BASAL	64	-0.45	-1.44	0.034	0.161	1	4060	tags=42%, list=23%, signal=54%
58	FAN_EMBRYONIC_CTX_NSC_2	224	-0.39	-1.43	0.006	0.162	1	4707	tags=38%, list=26%, signal=52%
59	GAUTAM_EYE_CORNEA_MONOCYTES	227	-0.39	-1.43	0.005	0.17	1	3914	tags=30%, list=22%, signal=38%
60	MENON_FETAL_KIDNEY_1_EMBRYONIC_RED_BLOOD_CELLS	69	-0.44	-1.42	0.033	0.175	1	3253	tags=33%, list=18%, signal=41%
61	CUI_DEVELOPING_HEART_C8_MACROPHAGE	249	-0.38	-1.41	0.002	0.193	1	1708	tags=17%, list=10%, signal=19%
62	GAO SMALL INTESTINE 24W C4 ENTEROCYTE PROGENITOR SUBT	89	-0.42	-1.4	0.044	0.2	1	3602	tags=33%, list=20%, signal=41%
63	AIZARANI_LIVER_C34_MHC_II_POS_B_CELLS	114	-0.41	-1.4	0.02	0.208	1	3084	tags=25%, list=17%, signal=31%
64	BUSSLINGER_DUODENAL_DIFFERENTIATING_STEM_CELLS	246	-0.38	-1.38	0.01	0.23	1	3200	tags=37%, list=18%, signal=44%
65	LAKE_ADULT_KIDNEY_C8_DECENDING_THIN_LIMB	231	-0.37	-1.38	0.011	0.228	1	3065	tags=29%, list=17%, signal=35%
66	GAUTAM_EYE_CHOROID_SCLERA_MELANOCYTES	145	-0.39	-1.38	0.027	0.23	1	4476	tags=40%, list=25%, signal=53%
67	HE_LIM_SUN_FETAL_LUNG_C1_MID_BASAL_CELL	129	-0.4	-1.38	0.023	0.229	1	3946	tags=38%, list=22%, signal=48%
68	LAKE_ADULT_KIDNEY_C9_THIN_ASCENDING_LIMB	230	-0.37	-1.38	0.011	0.228	1	2675	tags=28%, list=15%, signal=33%
69	GAUTAM_EYE_IRIS_CILIARY_BODY_MELANOCYTES	224	-0.37	-1.37	0.014	0.244	1	3886	tags=32%, list=22%, signal=40%
70	FAN_OVARY_CL18_B_LYMPHOCYTE	376	-0.36	-1.37	0.008	0.242	1	4611	tags=35%, list=26%, signal=46%
71	GAUTAM_EYE_IRIS_CILIARY_BODY_CYTOTOXIC_T_CELLS	68	-0.42	-1.36	0.065	0.246	1	4168	tags=41%, list=23%, signal=54%
72	AIZARANI_LIVER_C22_RESIDENT_B_CELLS_2	31	-0.49	-1.36	0.101	0.243	1	1970	tags=26%, list=11%, signal=29%
73	HAY_BONE_MARROW_NEUTROPHIL	409	-0.36	-1.36	0.003	0.243	1	2808	tags=23%, list=16%, signal=27%
74	TRAVAGLINI_LUNG_NATURAL_KILLER_CELL	117	-0.4	-1.36	0.035	0.245	1	3743	tags=31%, list=21%, signal=39%
75	DURANTE_ADULT_OLFACTORY_NEUROEPITHELIUM_CD4_T_CELLS	27	-0.5	-1.36	0.089	0.244	1	1555	tags=26%, list=9%, signal=28%
76	BUSSLINGER_ESOPHAGEAL_QUIESCENT_BASAL_CELLS	65	-0.43	-1.36	0.057	0.245	1	3803	tags=45%, list=21%, signal=57%
77	HAY_BONE_MARROW_MONOCYTE	193	-0.38	-1.36	0.016	0.244	1	2941	tags=24%, list=17%, signal=29%
78	ZHENG CORD BLOOD C5 SIMILAR TO HSC C6 PUTATIVE ALTEREI	60	-0.43	-1.36	0.069	0.242	1	4205	tags=40%, list=24%, signal=52%
79	TRAVAGLINI_LUNG_CLASSICAL_MONOCYTE_CELL	246	-0.36	-1.35	0.014	0.254	1	3385	tags=27%, list=19%, signal=33%
80	DESCARTES_FETAL_PANCREAS_SMOOTH_MUSCLE_CELLS	29	-0.5	-1.35	0.101	0.256	1	732	tags=14%, list=4%, signal=14%
81	GAO_LARGE_INTESTINE_24W_C11_PANETH_LIKE_CELL	284	-0.36	-1.34	0.008	0.261	1	3093	tags=25%, list=17%, signal=30%
82	LAKE ADULT KIDNEY C18 COLLECTING DUCT PRINCIPAL CELLS M	278	-0.36	-1.34	0.017	0.259	1	3299	tags=31%, list=19%, signal=37%
83	HE_LIM_SUN_FETAL_LUNG_C2_ADC_1_CELL	146	-0.38	-1.33	0.037	0.273	1	2432	tags=20%, list=14%, signal=23%
84	TRAVAGLINI_LUNG_PROLIFERATING_NK_T_CELL	118	-0.39	-1.33	0.061	0.276	1	4617	tags=37%, list=26%, signal=50%
85	DESCARTES_MAIN_FETAL_CLC_IL5RA_POSITIVE_CELLS	151	-0.37	-1.33	0.04	0.282	1	4607	tags=34%, list=26%, signal=46%
86	FAN_EMBRYONIC_CTX_BRAIN_EFFECTOR_T_CELL	108	-0.39	-1.32	0.047	0.288	1	3679	tags=30%, list=21%, signal=37%
87	FAN_EMBRYONIC_CTX_MICROGLIA_1	143	-0.38	-1.32	0.066	0.29	1	4710	tags=39%, list=27%, signal=53%
88	DESCARTES_MAIN_FETAL_ADRENOCORTICAL_CELLS	58	-0.42	-1.32	0.095	0.289	1	5244	tags=43%, list=30%, signal=61%
89	GAUTAM_EYE_CORNEA_CYTOTOXIC_T_CELLS	126	-0.38	-1.32	0.054	0.291	1	2923	tags=22%, list=16%, signal=26%
90	HE_LIM_SUN_FETAL_LUNG_C1_CLUB_CELL	60	-0.42	-1.31	0.102	0.296	1	1896	tags=22%, list=11%, signal=24%
91	HE_LIM_SUN_FETAL_LUNG_C1_EARLY_AIRWAY_PROGENITOR_CELL	35	-0.46	-1.31	0.125	0.296	1	5076	tags=43%, list=29%, signal=60%
92	DESCARTES_FETAL_LUNG_CSH1_CSH2_POSITIVE_CELLS	173	-0.37	-1.31	0.037	0.31	1	2796	tags=20%, list=16%, signal=23%
93	TRAVAGLINI_LUNG_CD4_MEMORY_EFFECTOR_T_CELL	61	-0.42	-1.3	0.079	0.31	1	4022	tags=41%, list=23%, signal=53%
94	FAN_EMBRYONIC_CTX_BIG_GROUPS_BRAIN_IMMUNE	125	-0.38	-1.3	0.068	0.309	1	3188	tags=27%, list=18%, signal=33%
95	DESCARTES FETAL EYE CORNEAL AND CONJUNCTIVAL EPITHELI/	214	-0.36	-1.29	0.043	0.33	1	2423	tags=30%, list=14%, signal=34%
96	HE_LIM_SUN_FETAL_LUNG_C5_PRO_B_CELL	423	-0.33	-1.29	0.021	0.335	1	4905	tags=35%, list=28%, signal=47%
97	HE_LIM_SUN_FETAL_LUNG_C1_SMG_BASAL_CELL	347	-0.34	-1.29	0.03	0.332	1	4033	tags=29%, list=23%, signal=37%
98	HE_LIM_SUN_FETAL_LUNG_C1_PROXIMAL_SECRETORY_1_CELL	92	-0.39	-1.29	0.085	0.338	1	1909	tags=20%, list=11%, signal=22%
99	GAUTAM_EYE_IRIS_CILIARY_BODY_PUTATIVE_STEM_CELLS	253	-0.35	-1.29	0.042	0.338	1	5375	tags=39%, list=30%, signal=55%
100	BUSSLINGER_GASTRIC_PPP1R1B_POSITIVE_CELLS	92	-0.39	-1.28	0.089	0.339	1	3200	tags=50%, list=18%, signal=61%
101	DESCARTES_MAIN_FETAL_SMOOTH_MUSCLE_CELLS	37	-0.45	-1.28	0.128	0.338	1	1109	tags=14%, list=6%, signal=14%
102	CUI_DEVELOPING_HEART_TRABECULAR_VENTRICULAR_CARDIOMYC	101	-0.38	-1.28	0.089	0.338	1	3707	tags=33%, list=21%, signal=41%
103	BUSSLINGER_DUODENAL_STEM_CELLS	260	-0.34	-1.28	0.041	0.336	1	3200	tags=33%, list=18%, signal=39%
104	FAN_EMBRYONIC_CTX_BRAIN_NAIVE_LIKE_T_CELL	132	-0.37	-1.28	0.082	0.337	1	3532	tags=29%, list=20%, signal=36%
105	ZHONG_PFC_MAJOR_TYPES_NPCS	133	-0.37	-1.28	0.083	0.339	1	4617	tags=35%, list=26%, signal=47%
106	BUSSLINGER_ESOPHAGEAL_PROLIFERATING_BASAL_CELLS	58	-0.41	-1.28	0.121	0.34	1	3559	tags=47%, list=20%, signal=58%
107	HE_LIM_SUN_FETAL_LUNG_C2_PROMONOCYTE_LIKE_CELL	262	-0.34	-1.28	0.037	0.338	1	5271	tags=39%, list=30%, signal=55%
108	HE_LIM_SUN_FETAL_LUNG_C4_ILC2_CELL	139	-0.37	-1.27	0.083	0.342	1	2846	tags=25%, list=16%, signal=30%
109	GAUTAM_EYE_CORNEA_FIBROBLASTS	144	-0.36	-1.27	0.078	0.339	1	2103	tags=21%, list=12%, signal=23%
110	HE_LIM_SUN_FETAL_LUNG_C2_DC1_CELL	285	-0.34	-1.27	0.035	0.338	1	2683	tags=21%, list=15%, signal=24%
111	TRAVAGLINI_LUNG_MYELOID_DENDRITIC_TYPE_1_CELL	16	-0.53	-1.27	0.17	0.337	1	4164	tags=56%, list=23%, signal=73%
112	FAN_EMBRYONIC_CTX_BIG_GROUPS_MICROGLIA	336	-0.33	-1.27	0.027	0.335	1	2215	tags=20%, list=12%, signal=22%
113	DURANTE ADULT OLFACTORY NEUROEPITHELIUM RESPIRATORY (	26	-0.47	-1.27	0.166	0.338	1	1335	tags=27%, list=8%, signal=29%
114	TRAVAGLINI_LUNG_PLASMACYTOID_DENDRITIC_CELL	104	-0.37	-1.26	0.102	0.347	1	2988	tags=23%, list=17%, signal=28%
115	FAN_OVARY_CL5_HEALTHY_SELECTABLE_FOLLICLE_THECAL_CELL	289	-0.34	-1.26	0.045	0.354	1	3505	tags=29%, list=20%, signal=36%
116	FAN_EMBRYONIC_CTX_BRAIN_B_CELL	80	-0.38	-1.26	0.128	0.355	1	3084	tags=24%, list=17%, signal=29%
117	HE_LIM_SUN_FETAL_LUNG_C3_DEFINITIVE_ERYTHROBLAST	24	-0.47	-1.25	0.159	0.365	1	690	tags=17%, list=4%, signal=17%
118	RUBENSTEIN_SKELETAL_MUSCLE_SMOOTH_MUSCLE_CELLS	401	-0.33	-1.25	0.044	0.363	1	3178	tags=26%, list=18%, signal=31%
119	LAKE_ADULT_KIDNEY_C10_THIN_ASCENDING_LIMB	302	-0.33	-1.25	0.049	0.378	1	4018	tags=34%, list=23%, signal=44%
120	FAN_OVARY_CL14_MATURE_SMOOTH_MUSCLE_CELL	278	-0.33	-1.24	0.058	0.378	1	3980	tags=32%, list=22%, signal=41%
121	DESCARTES_FETAL_MUSCLE_LYMPHOID_CELLS	97	-0.37	-1.24	0.126	0.397	1	3955	tags=30%, list=22%, signal=38%
122	GAO_LARGE_INTESTINE_ADULT_CH_MKI67HIGH_CELLS	90	-0.37	-1.24	0.134	0.397	1	4607	tags=36%, list=26%, signal=48%
123	HE_LIM_SUN_FETAL_LUNG_C0_EARLY_FIBROBLAST	68	-0.39	-1.24	0.164	0.395	1	4617	tags=40%, list=26%, signal=53%
124	GAUTAM_EYE_IRIS_CILIARY_BODY_SMOOTH_MUSCLE_CELLS	243	-0.33	-1.23	0.088	0.396	1	4596	tags=36%, list=26%, signal=48%
125	TRAVAGLINI_LUNG_BRONCHIAL_VESSEL_2_CELL	243	-0.33	-1.23	0.081	0.396	1	4115	tags=33%, list=23%, signal=43%
126	AIZARANI_LIVER_C5_NK_NKT_CELLS_3	112	-0.36	-1.23	0.123	0.395	1	3901	tags=31%, list=22%, signal=40%
127	DESCARTES_FETAL_HEART_ERYTHROBLASTS	241	-0.34	-1.23	0.069	0.393	1	4920	tags=41%, list=28%, signal=55%
128	FAN_EMBRYONIC_CTX_BIG_GROUPS_CAJAL_RETZIUS	447	-0.32	-1.23	0.051	0.405	1	2555	tags=18%, list=14%, signal=21%
129	HE_LIM_SUN_FETAL_LUNG_C2_APOE_POS_M2_MACROPHAGE_CELL	431	-0.32	-1.22	0.04	0.406	1	5082	tags=38%, list=29%, signal=52%
130	HE_LIM_SUN_FETAL_LUNG_C1_PROXIMAL_SECRETORY_2_CELL	268	-0.33	-1.22	0.081	0.414	1	1909	tags=19%, list=11%, signal=21%
131	HE_LIM_SUN_FETAL_LUNG_C3_MID_CAP_CELL	51	-0.4	-1.22	0.188	0.416	1	1529	tags=16%, list=9%, signal=17%
132	DURANTE_ADULT_OLFACTORY_NEUROEPITHELIUM_DENDRITIC_CEL	99	-0.36	-1.22	0.126	0.415	1	918	tags=12%, list=5%, signal=13%
133	AIZARANI_LIVER_C30_HEPATOCYTES_4	371	-0.32	-1.22	0.067	0.416	1	3146	tags=21%, list=18%, signal=25%
134	TRAVAGLINI_LUNG_CAPILLARY_INTERMEDIATE_2_CELL	337	-0.32	-1.22	0.074	0.413	1	4164	tags=33%, list=23%, signal=43%
135	AIZARANI_LIVER_C38_RESIDENT_B_CELLS_3	22	-0.46	-1.22	0.217	0.412	1	1970	tags=23%, list=11%, signal=26%
136	HE_LIM_SUN_FETAL_LUNG_C4_ACTIVATED_NK_CELL	46	-0.4	-1.21	0.18	0.417	1	4558	tags=41%, list=26%, signal=55%
137	DESCARTES_FETAL_HEART_EPICARDIAL_FAT_CELLS	90	-0.37	-1.21	0.152	0.416	1	2171	tags=18%, list=12%, signal=20%
138	AIZARANI_LIVER_C8_RESIDENT_B_CELLS_1	33	-0.44	-1.21	0.199	0.414	1	3164	tags=33%, list=18%, signal=40%
139	TRAVAGLINI_LUNG_NATURAL_KILLER_T_CELL	26	-0.45	-1.21	0.202	0.415	1	1480	tags=19%, list=8%, signal=21%
140	HE_LIM_SUN_FETAL_LUNG_C2_ADC_2_CELL	85	-0.37	-1.21	0.162	0.415	1	4114	tags=39%, list=23%, signal=50%
141	HE_LIM_SUN_FETAL_LUNG_C2_PDC_CELL	131	-0.35	-1.21	0.135	0.42	1	3744	tags=27%, list=21%, signal=34%
142	TRAVAGLINI_LUNG_NONCLASSICAL_MONOCYTE_CELL	169	-0.34	-1.2	0.127	0.434	1	4229	tags=34%, list=24%, signal=44%
143	DURANTE ADULT OLFACTORY NEUROEPITHELIUM RESPIRATORY I	30	-0.43	-1.2	0.214	0.442	1	1843	tags=30%, list=10%, signal=33%
144	BUSSLINGER_ESOPHAGEAL_DENDRITIC_CELLS	184	-0.33	-1.2	0.149	0.442	1	4335	tags=34%, list=24%, signal=45%
145	TRAVAGLINI_LUNG_CD8_NAIVE_T_CELL	122	-0.35	-1.2	0.164	0.441	1	3679	tags=27%, list=21%, signal=34%
146	HE_LIM_SUN_FETAL_LUNG_C4_ILC3_CELL	66	-0.38	-1.19	0.193	0.443	1	2548	tags=23%, list=14%, signal=26%
147	DESCARTES_FETAL_CEREBELLUM_MICROGLIA	477	-0.31	-1.19	0.07	0.452	1	2919	tags=20%, list=16%, signal=23%

148	DESCARTES_FETAL_KIDNEY_URETERIC_BUD_CELLS	237	-0.32	-1.19	0.119	0.452	1	1909	tags=22%, list=11%, signal=24%
149	CUI_DEVELOPING_HEART_SMOOTH_MUSCLE_CELL	46	-0.4	-1.19	0.214	0.451	1	551	tags=13%, list=3%, signal=13%
150	DESCARTES_MAIN_FETAL_SYNCYTIOTROPHOBLASTS_AND_VILLOUS	70	-0.37	-1.19	0.196	0.449	1	4285	tags=30%, list=24%, signal=39%
151	HE LIM SUN FETAL LUNG C5 CD5 POS CCL22 NEG MATURE B C	136	-0.34	-1.18	0.153	0.454	1	4089	tags=32%, list=23%, signal=42%
152	DESCARTES_FETAL_EYE_SMOOTH_MUSCLE_CELLS	56	-0.38	-1.17	0.224	0.481	1	4079	tags=30%, list=23%, signal=39%
153	TRAVAGLINI_LUNG_GOBLET_CELL	130	-0.34	-1.17	0.19	0.486	1	1821	tags=24%, list=10%, signal=26%
154	GAO_STOMACH_24W_C1_PROCRPOS_MULTIPOTENT_PROGENITOR	33	-0.41	-1.17	0.248	0.483	1	3379	tags=27%, list=19%, signal=34%
155	HE_LIM_SUN_FETAL_LUNG_C2_NEUTROPHIL_CELL	52	-0.38	-1.17	0.231	0.49	1	1392	tags=19%, list=8%, signal=21%
156	GAUTAM_EYE_IRIS_CILIARY_BODY_ACTIVATED_T_CELLS	106	-0.34	-1.16	0.223	0.506	1	3659	tags=28%, list=21%, signal=35%
157	DESCARTES_FETAL_INTESTINE_LYMPHOID_CELLS	126	-0.34	-1.16	0.209	0.507	1	3955	tags=26%, list=22%, signal=33%
158	DESCARTES_MAIN_FETAL_ANTIGEN_PRESENTING_CELLS	87	-0.35	-1.16	0.232	0.508	1	2914	tags=24%, list=16%, signal=29%
159	HE_LIM_SUN_FETAL_LUNG_C0_MESENCHYMAL_2_CELL	270	-0.31	-1.16	0.164	0.507	1	4617	tags=32%, list=26%, signal=42%
160	FAN_EMBRYONIC_CTX_BRAIN_MYELOID	120	-0.34	-1.16	0.217	0.51	1	1652	tags=13%, list=9%, signal=14%
161	DESCARTES_FETAL_CEREBRUM_MICROGLIA	348	-0.31	-1.16	0.156	0.508	1	2715	tags=17%, list=15%, signal=20%
162	CUI_DEVELOPING_HEART_C1_5TH_WEEK_CARDIAC_CELLS	77	-0.36	-1.16	0.227	0.509	1	2316	tags=22%, list=13%, signal=25%
163	HAY_BONE_MARROW_PLATELET	225	-0.31	-1.15	0.175	0.519	1	3831	tags=25%, list=22%, signal=31%
164	ZHONG_PFC_MAJOR_TYPES_MICROGLIA	399	-0.3	-1.15	0.142	0.523	1	2067	tags=17%, list=12%, signal=19%
165	ZHONG_PFC_C3_UNKNOWN_INP	36	-0.41	-1.15	0.261	0.526	1	4607	tags=42%, list=26%, signal=56%
166	DESCARTES_MAIN_FETAL_IGFBP1_DKK1_POSITIVE_CELLS	128	-0.33	-1.15	0.228	0.526	1	3065	tags=22%, list=17%, signal=26%
167	HE_LIM_SUN_FETAL_LUNG_C0_VASCULAR_SMC_2_CELL	153	-0.32	-1.15	0.205	0.525	1	1313	tags=12%, list=7%, signal=13%
168	HE_LIM_SUN_FETAL_LUNG_C7_SCHWANN_PRECURSOR_CELL	219	-0.31	-1.15	0.184	0.523	1	4803	tags=32%, list=27%, signal=43%
169	TRAVAGLINI_LUNG_ADVENTITIAL_FIBROBLAST_CELL	274	-0.31	-1.14	0.173	0.532	1	3212	tags=24%, list=18%, signal=29%
170	HAY_BONE_MARROW_PRO_B	265	-0.31	-1.14	0.19	0.532	1	3755	tags=28%, list=21%, signal=34%
171	TRAVAGLINI_LUNG_CAPILLARY_AEROCYTE_CELL	41	-0.39	-1.14	0.28	0.532	1	3096	tags=32%, list=17%, signal=38%
172	DESCARTES_FETAL_KIDNEY_MEGAKARYOCYTES	102	-0.34	-1.14	0.236	0.529	1	3351	tags=24%, list=19%, signal=29%
173	ZHONG_PFC_C5_BCL11B_CALB2_POS_INP	22	-0.43	-1.14	0.294	0.528	1	2438	tags=27%, list=14%, signal=32%
174	LAKE ADULT KIDNEY C19 COLLECTING DUCT INTERCALATED CEL	281	-0.3	-1.14	0.184	0.53	1	3384	tags=29%, list=19%, signal=35%
175	HE_LIM_SUN_FETAL_LUNG_C4_TREG_CELL	34	-0.41	-1.13	0.284	0.537	1	4194	tags=35%, list=24%, signal=46%
176	GAUTAM_EYE_CORNEA_TGFBI_HIGH_EPITHELIAL_CELLS	100	-0.34	-1.13	0.251	0.538	1	3535	tags=30%, list=20%, signal=37%
177	ZHENG_CORD_BLOOD_C8_PUTATIVE_LYMPHOID_PRIMED_MULTIPOT	77	-0.35	-1.13	0.247	0.543	1	2914	tags=29%, list=16%, signal=34%
178	GAO_ESOPHAGUS_25W_C4_FGFR1HIGH_EPITHELIAL_CELLS	180	-0.31	-1.13	0.229	0.547	1	3996	tags=27%, list=22%, signal=34%
179	DESCARTES_MAIN_FETAL_TROPHOBLAST_GIANT_CELLS	110	-0.33	-1.13	0.244	0.544	1	2526	tags=15%, list=14%, signal=18%
180	DESCARTES_MAIN_FETAL_CSH1_CSH2_POSITIVE_CELLS	66	-0.35	-1.12	0.297	0.566	1	4312	tags=33%, list=24%, signal=44%
181	FAN OVARY CL6 PUTATIVE EARLY ATRETIC FOLLICLE THECAL CE	284	-0.3	-1.12	0.207	0.569	1	3218	tags=25%, list=18%, signal=30%
182	DESCARTES_FETAL_MUSCLE_SMOOTH_MUSCLE_CELLS	67	-0.35	-1.12	0.268	0.569	1	1356	tags=12%, list=8%, signal=13%
183	DESCARTES_FETAL_ADRENAL_LYMPHOID_CELLS	128	-0.32	-1.11	0.281	0.576	1	3955	tags=28%, list=22%, signal=36%
184	GAO SMALL INTESTINE 24W C3 ENTEROCYTE PROGENITOR SUBT	38	-0.38	-1.11	0.299	0.587	1	3108	tags=34%, list=17%, signal=41%
185	HE_LIM_SUN_FETAL_LUNG_C2_S100A12_HI_CLASSICAL_MONOCYTE	30	-0.4	-1.11	0.313	0.589	1	814	tags=10%, list=5%, signal=10%
186	BUSSLINGER_GASTRIC_REG3A_POSITIVE_CELLS	29	-0.4	-1.11	0.332	0.593	1	1229	tags=31%, list=7%, signal=33%
187	HE_LIM_SUN_FETAL_LUNG_C0_MID_AIRWAY_SMC_2_CELL	177	-0.31	-1.1	0.262	0.595	1	3980	tags=25%, list=22%, signal=32%
188	HE LIM SUN FETAL LUNG C3 CYCLING DEFINITIVE ERYTHROBLAS	398	-0.29	-1.1	0.227	0.597	1	4686	tags=33%, list=26%, signal=44%
189	DESCARTES_MAIN_FETAL_VASCULAR_ENDOTHELIAL_CELLS	41	-0.37	-1.1	0.324	0.611	1	395	tags=7%, list=2%, signal=7%
190	HE LIM SUN FETAL LUNG C1 PROXIMAL SECRETORY PROGENITO	120	-0.32	-1.1	0.298	0.609	1	4509	tags=38%, list=25%, signal=50%
191	DESCARTES_MAIN_FETAL_THYMIC_EPITHELIAL_CELLS	133	-0.31	-1.1	0.303	0.607	1	3781	tags=23%, list=21%, signal=29%
192	HE_LIM_SUN_FETAL_LUNG_C1_EARLY_TIP_CELL	127	-0.32	-1.1	0.297	0.604	1	4617	tags=33%, list=26%, signal=44%
193	AIZARANI_LIVER_C28_NK_NKT_CELLS_6	90	-0.33	-1.09	0.321	0.608	1	3950	tags=29%, list=22%, signal=37%
194	HAY_BONE_MARROW_CD34_POS_GRAN	23	-0.42	-1.09	0.356	0.606	1	3328	tags=26%, list=19%, signal=32%
195	GAO_ESOPHAGUS_25W_C3_FGFR1LOW_EPITHELIAL_CELLS	46	-0.36	-1.08	0.356	0.637	1	6498	tags=52%, list=37%, signal=82%
196	ZHONG_PFC_MAJOR_TYPES_ASTROCYTES	307	-0.29	-1.08	0.287	0.635	1	3367	tags=24%, list=19%, signal=29%
197	DESCARTES_FETAL_PLACENTA_MYELOID_CELLS	121	-0.31	-1.08	0.317	0.639	1	1619	tags=16%, list=9%, signal=17%
198	DESCARTES_FETAL_SPLEEN_LYMPHOID_CELLS	92	-0.32	-1.08	0.343	0.637	1	1466	tags=13%, list=8%, signal=14%
199	DESCARTES_FETAL_PANCREAS_MYELOID_CELLS	176	-0.3	-1.08	0.303	0.635	1	438	tags=8%, list=2%, signal=8%
200	MANNO_MIDBRAIN_NEUROTYPES_HPROGBP	277	-0.29	-1.08	0.289	0.633	1	3370	tags=22%, list=19%, signal=27%
201	HE_LIM_SUN_FETAL_LUNG_C6_DEUTEROSOMAL_CELL	196	-0.3	-1.08	0.328	0.635	1	4659	tags=32%, list=26%, signal=43%
202	ZHONG_PFC_C4_PTGDS_POS_OPC	136	-0.31	-1.08	0.328	0.633	1	3247	tags=24%, list=18%, signal=29%
203	DESCARTES_FETAL_LUNG_VASCULAR_ENDOTHELIAL_CELLS	39	-0.37	-1.08	0.37	0.631	1	356	tags=8%, list=2%, signal=8%
204	CUI_DEVELOPING_HEART_CARDIAC_FIBROBLASTS	97	-0.32	-1.08	0.335	0.629	1	1260	tags=11%, list=7%, signal=12%
205	DESCARTES_FETAL_ADRENAL_CSH1_CSH2_POSITIVE_CELLS	132	-0.31	-1.07	0.332	0.636	1	4161	tags=30%, list=23%, signal=39%
206	DESCARTES_FETAL_CEREBELLUM_PURKINJE_NEURONS	44	-0.36	-1.07	0.358	0.633	1	2130	tags=16%, list=12%, signal=18%
207	AIZARANI_LIVER_C32_MVECS_3	111	-0.31	-1.07	0.342	0.633	1	2473	tags=17%, list=14%, signal=20%
208	CUI_DEVELOPING_HEART_C7_MAST_CELL	145	-0.31	-1.07	0.33	0.631	1	3328	tags=26%, list=19%, signal=31%
209	CUI_DEVELOPING_HEART_VASCULAR_ENDOTHELIAL_CELL	171	-0.3	-1.07	0.342	0.635	1	3304	tags=27%, list=19%, signal=33%
210	DESCARTES_FETAL_KIDNEY_MYELOID_CELLS	147	-0.3	-1.07	0.352	0.65	1	1446	tags=12%, list=8%, signal=12%
211	FAN_OVARY_CL17_PUTATIVE_APOPTOTIC_SMOOTH_MUSCLE_CELL	205	-0.29	-1.06	0.326	0.663	1	3074	tags=24%, list=17%, signal=29%
212	GAUTAM EYE IRIS CILIARY BODY CRYAA HIGH CILIARY BODY CE	77	-0.33	-1.06	0.367	0.661	1	4880	tags=40%, list=27%, signal=55%
213	HE_LIM_SUN_FETAL_LUNG_C2_CXCL9_POS_MACROPHAGE_CELL	108	-0.31	-1.06	0.378	0.659	1	3597	tags=30%, list=20%, signal=37%
214	TRAVAGLINI_LUNG_BASOPHIL_MAST_2_CELL	458	-0.28	-1.05	0.331	0.673	1	4735	tags=32%, list=27%, signal=42%
215	RUBENSTEIN_SKELETAL_MUSCLE_PERICYTES	131	-0.3	-1.05	0.387	0.673	1	2944	tags=26%, list=17%, signal=31%
216	FAN_EMBRYONIC_CTX_MICROGLIA_3	18	-0.42	-1.04	0.373	0.696	1	1821	tags=28%, list=10%, signal=31%
217	DESCARTES_FETAL_HEART_ENDOCARDIAL_CELLS	28	-0.38	-1.04	0.397	0.695	1	2782	tags=18%, list=16%, signal=21%
218	HE_LIM_SUN_FETAL_LUNG_C4_ILCP_CELL	168	-0.29	-1.04	0.381	0.701	1	3009	tags=21%, list=17%, signal=25%
219	DURANTE_ADULT_OLFACTORY_NEUROEPITHELIUM_NK_CELLS	68	-0.32	-1.04	0.401	0.71	1	2976	tags=22%, list=17%, signal=26%
220	FAN_OVARY_CL9_PUTATIVE_APOPTOTIC_ENDOTHELIAL_CELL	328	-0.28	-1.04	0.389	0.709	1	3811	tags=28%, list=21%, signal=35%
221	GAUTAM_EYE_IRIS_CILIARY_BODY_CILIARY_BODY_CELLS	94	-0.31	-1.04	0.407	0.711	1	1350	tags=14%, list=8%, signal=15%
222	CUI_DEVELOPING_HEART_ENDOCARDIAL_CELL	53	-0.34	-1.04	0.418	0.709	1	2786	tags=19%, list=16%, signal=22%
223	DESCARTES_MAIN_FETAL_HEMATOPOIETIC_STEM_CELLS	35	-0.37	-1.03	0.428	0.707	1	4787	tags=26%, list=27%, signal=35%
224	TRAVAGLINI_LUNG_LIPOFIBROBLAST_CELL	139	-0.29	-1.03	0.406	0.713	1	4536	tags=35%, list=26%, signal=46%
225	GAUTAM_EYE_CORNEA_CONJUNCTIVAL_CELLS	89	-0.31	-1.03	0.402	0.712	1	4749	tags=46%, list=27%, signal=63%
226	HAY_BONE_MARROW_FOLLICULAR_B_CELL	120	-0.3	-1.03	0.411	0.714	1	4748	tags=32%, list=27%, signal=43%
227	HE_LIM_SUN_FETAL_LUNG_C5_CD5_NEG_MATURE_B_CELL	46	-0.34	-1.03	0.443	0.718	1	2473	tags=26%, list=14%, signal=30%
228	GAUTAM_EYE_CHOROID_SCLERA_MONOCYTES	120	-0.3	-1.03	0.413	0.717	1	5100	tags=34%, list=29%, signal=48%
229	BUSSLINGER_DUODENAL_MATURE_ENTEROCYTES	241	-0.28	-1.03	0.418	0.717	1	3707	tags=27%, list=21%, signal=33%
230	ZHONG_PFC_C9_ORG_OTHER	81	-0.31	-1.02	0.436	0.725	1	3513	tags=31%, list=20%, signal=38%
231	HU_FETAL_RETINA_MICROGLIA	347	-0.27	-1.02	0.434	0.727	1	4144	tags=28%, list=23%, signal=35%
232	MENON_FETAL_KIDNEY_7_LOOPOF_HENLE_CELLS_DISTAL	244	-0.27	-1.02	0.443	0.727	1	3907	tags=29%, list=22%, signal=36%
233	DESCARTES_FETAL_PLACENTA_SYNCYTIOTROPHOBLASTS_AND_VIL	52	-0.34	-1.02	0.448	0.727	1	4295	tags=33%, list=24%, signal=43%
234	HAY_BONE_MARROW_EARLY_ERYTHROBLAST	85	-0.31	-1.02	0.457	0.726	1	4913	tags=46%, list=28%, signal=63%
235	MENON_FETAL_KIDNEY_5_PROXIMAL_TUBULE_CELLS	97	-0.3	-1.01	0.435	0.734	1	3005	tags=25%, list=17%, signal=30%
236	FAN_OVARY_CL7_ANGEIOGENIC_ENDOTHELIAL_CELL	224	-0.27	-1.01	0.452	0.755	1	4211	tags=30%, list=24%, signal=39%
237	GAUTAM_EYE_IRIS_CILIARY_BODY_MEG3_HIGH_FIBROBLASTS	102	-0.3	-1.01	0.458	0.754	1	2152	tags=18%, list=12%, signal=20%
238	DESCARTES_MAIN_FETAL_AFP_ALB_POSITIVE_CELLS	80	-0.31	-1	0.462	0.762	1	3715	tags=19%, list=21%, signal=24%
239	GAUTAM_EYE_CHOROID_SCLERA_FIBROBLASTS	193	-0.28	-1	0.461	0.759	1	4391	tags=32%, list=25%, signal=42%
240	AIZARANI_LIVER_C23_KUPFFER_CELLS_3	192	-0.28	-1	0.465	0.76	1	1602	tags=13%, list=9%, signal=14%
241	HE_LIM_SUN_FETAL_LUNG_C0_PERICYTE	181	-0.28	-1	0.487	0.765	1	1261	tags=10%, list=7%, signal=11%
242	TRAVAGLINI_LUNG_CAPILLARY_INTERMEDIATE_1_CELL	150	-0.28	-1	0.482	0.769	1	3096	tags=21%, list=17%, signal=26%
243	ZHENG_CORD_BLOOD_C6_HSC_MULTIPOTENT_PROGENITOR	80	-0.3	-0.99	0.477	0.776	1	1729	tags=18%, list=10%, signal=19%

244	FAN_OVARY_CL3_MATURE_CUMULUS_GRANULOSA_CELL_1	224	-0.27	-0.99	0.49	0.775	1	4555	tags=28%, list=26%, signal=37%
245	AIZARANI_LIVER_C25_KUPFFER_CELLS_4	150	-0.28	-0.99	0.492	0.779	1	1499	tags=15%, list=8%, signal=16%
246	GAUTAM_EYE_CHOROID_SCLERA_ACTIVATED_T_CELLS	105	-0.29	-0.99	0.497	0.779	1	3596	tags=27%, list=20%, signal=33%
247	HE_LIM_SUN_FETAL_LUNG_C2_HSC_ELP_CELL	352	-0.26	-0.99	0.507	0.779	1	3593	tags=23%, list=20%, signal=28%
248	HE_LIM_SUN_FETAL_LUNG_C0_MESENCHYMAL_1_CELL	269	-0.27	-0.99	0.514	0.776	1	3476	tags=22%, list=20%, signal=27%
249	MANNO_MIDBRAIN_NEUROTYPES_HPROGFPM	318	-0.26	-0.98	0.53	0.787	1	3353	tags=23%, list=19%, signal=28%
250	GAUTAM EYE IRIS CILIARY BODY PIGMENTED CILIARY BODY CEL	111	-0.29	-0.98	0.52	0.787	1	2505	tags=14%, list=14%, signal=17%
251	DESCARTES_FETAL_SPLEEN_MESOTHELIAL_CELLS	181	-0.27	-0.98	0.505	0.784	1	2423	tags=17%, list=14%, signal=20%
252	TRAVAGLINI_LUNG_B_CELL	142	-0.28	-0.98	0.508	0.784	1	3084	tags=23%, list=17%, signal=27%
253	FAN_EMBRYONIC_CTX_BRAIN_ENDOTHELIAL_1	410	-0.25	-0.98	0.538	0.782	1	2643	tags=16%, list=15%, signal=18%
254	DESCARTES_FETAL_EYE_PDE11A_FAM19A2_POSITIVE_CELLS	18	-0.38	-0.98	0.498	0.785	1	5262	tags=33%, list=30%, signal=47%
255	GAUTAM_EYE_IRIS_CILIARY_BODY_SCHWANN_CELLS	85	-0.3	-0.98	0.509	0.784	1	4262	tags=27%, list=24%, signal=35%
256	DESCARTES_MAIN_FETAL_PDE1C_ACSM3_POSITIVE_CELLS	236	-0.27	-0.98	0.531	0.784	1	2574	tags=19%, list=14%, signal=22%
257	DESCARTES_FETAL_CEREBRUM_MEGAKARYOCYTES	317	-0.26	-0.97	0.558	0.793	1	3935	tags=22%, list=22%, signal=28%
258	HE_LIM_SUN_FETAL_LUNG_C2_PLATELET_CELL	327	-0.25	-0.97	0.576	0.808	1	4492	tags=28%, list=25%, signal=37%
259	HAY_BONE_MARROW_CD34_POS_PRE_B	85	-0.29	-0.97	0.53	0.807	1	3716	tags=27%, list=21%, signal=34%
260	GAUTAM EYE IRIS CILIARY BODY CILIARY BODY ENDOTHELIAL CI	324	-0.25	-0.96	0.544	0.807	1	4981	tags=29%, list=28%, signal=40%
261	ZHONG_PFC_C2_SOX5_BCL11B_POS_EXCITATORY_NEURON	24	-0.37	-0.96	0.528	0.819	1	3358	tags=29%, list=19%, signal=36%
262	DESCARTES_FETAL_THYMUS_THYMIC_EPITHELIAL_CELLS	198	-0.26	-0.95	0.55	0.83	1	3946	tags=25%, list=22%, signal=31%
263	HE_LIM_SUN_FETAL_LUNG_C2_PROMYELOCYTE_LIKE_CELL	43	-0.32	-0.95	0.54	0.835	1	2664	tags=19%, list=15%, signal=22%
264	DESCARTES_FETAL_LIVER_MYELOID_CELLS	159	-0.27	-0.95	0.591	0.836	1	1288	tags=12%, list=7%, signal=13%
265	VANGURP_PANCREATIC_BETA_CELL	108	-0.28	-0.95	0.553	0.836	1	3544	tags=23%, list=20%, signal=29%
266	FAN_EMBRYONIC_CTX_BIG_GROUPS_GLIAL	130	-0.27	-0.95	0.572	0.833	1	3396	tags=28%, list=19%, signal=34%
267	HU_FETAL_RETINA_RGC	413	-0.24	-0.95	0.641	0.831	1	3747	tags=21%, list=21%, signal=26%
268	DESCARTES_FETAL_HEART_SMOOTH_MUSCLE_CELLS	36	-0.33	-0.94	0.531	0.839	1	1704	tags=11%, list=10%, signal=12%
269	ZHONG_PFC_MAJOR_TYPES_INTERNEURON	16	-0.39	-0.94	0.556	0.839	1	148	tags=6%, list=1%, signal=6%
270	DESCARTES_MAIN_FETAL_HORIZONTAL_CELLS	28	-0.35	-0.94	0.554	0.838	1	1326	tags=14%, list=7%, signal=15%
271	HE_LIM_SUN_FETAL_LUNG_C4_T_ALPHA_BETA_ENTRY_CELL	62	-0.3	-0.94	0.574	0.837	1	3258	tags=23%, list=18%, signal=28%
272	ZHENG CORD BLOOD C4 PUTATIVE EARLY ERYTHROID COMMITM	89	-0.28	-0.94	0.553	0.834	1	4817	tags=40%, list=27%, signal=55%
273	DESCARTES_FETAL_STOMACH_MYELOID_CELLS	64	-0.3	-0.94	0.581	0.833	1	1708	tags=14%, list=10%, signal=16%
274	DESCARTES_FETAL_STOMACH_LYMPHOID_CELLS	72	-0.29	-0.94	0.595	0.833	1	2715	tags=15%, list=15%, signal=18%
275	DESCARTES_FETAL_PLACENTA_AFP_ALB_POSITIVE_CELLS	146	-0.27	-0.94	0.601	0.835	1	1861	tags=10%, list=10%, signal=11%
276	GAO_STOMACH_24W_C5_PUTATIVE_PIT_CELL_PROGENITOR	53	-0.3	-0.94	0.568	0.836	1	5192	tags=40%, list=29%, signal=56%
277	CUI_DEVELOPING_HEART_VALVAR_ENDOTHELIAL_CELL	124	-0.27	-0.94	0.589	0.833	1	4536	tags=32%, list=26%, signal=43%
278	DESCARTES_FETAL_PLACENTA_TROPHOBLAST_GIANT_CELLS	48	-0.31	-0.94	0.581	0.835	1	1183	tags=17%, list=7%, signal=18%
279	LAKE ADULT KIDNEY C5 PROXIMAL TUBULE EPITHELIAL CELLS S	365	-0.25	-0.93	0.657	0.835	1	3586	tags=20%, list=20%, signal=25%
280	DESCARTES_MAIN_FETAL_MEGAKARYOCYTES	159	-0.27	-0.93	0.616	0.833	1	3558	tags=22%, list=20%, signal=27%
281	TRAVAGLINI_LUNG_FIBROMYOCYTE_CELL	22	-0.36	-0.93	0.57	0.831	1	5220	tags=41%, list=29%, signal=58%
282	DESCARTES_MAIN_FETAL_EPICARDIAL_FAT_CELLS	34	-0.33	-0.93	0.563	0.829	1	2113	tags=21%, list=12%, signal=23%
283	HE_LIM_SUN_FETAL_LUNG_C1_LATE_AIRWAY_PROGENITOR_CELL	22	-0.36	-0.93	0.575	0.829	1	2678	tags=23%, list=15%, signal=27%
284	ZHONG_PFC_C6_DLX5_GAD1_GAD2_POS_INTERNEURON	15	-0.39	-0.93	0.58	0.837	1	1442	tags=13%, list=8%, signal=14%
285	DESCARTES_FETAL_THYMUS_ANTIGEN_PRESENTING_CELLS	152	-0.26	-0.92	0.62	0.843	1	2914	tags=19%, list=16%, signal=23%
286	DESCARTES_FETAL_KIDNEY_LYMPHOID_CELLS	151	-0.26	-0.92	0.614	0.842	1	3957	tags=24%, list=22%, signal=30%
287	LAKE ADULT KIDNEY C17 COLLECTING SYSTEM PCS STRESSED	222	-0.25	-0.92	0.66	0.841	1	3721	tags=24%, list=21%, signal=30%
288	HAY_BONE_MARROW_PLASMA_CELL	94	-0.28	-0.92	0.599	0.841	1	3277	tags=23%, list=18%, signal=29%
289	LAKE ADULT KIDNEY C6 PROXIMAL TUBULE EPITHELIAL CELLS F	160	-0.26	-0.92	0.635	0.841	1	3443	tags=22%, list=19%, signal=27%
290	DESCARTES_FETAL_INTESTINE_SMOOTH_MUSCLE_CELLS	43	-0.31	-0.92	0.596	0.844	1	1248	tags=14%, list=7%, signal=15%
291	FAN OVARY CL1 GPRC5A TNFRS12A HIGH SELECTABLE FOLLICLE	344	-0.24	-0.92	0.702	0.848	1	3133	tags=25%, list=18%, signal=29%
292	DESCARTES_FETAL_CEREBRUM_INHIBITORY_NEURONS	36	-0.32	-0.92	0.592	0.847	1	520	tags=8%, list=3%, signal=9%
293	CUI DEVELOPING HEART 5TH WEEK VENTRICULAR CARDIOMYOCY	73	-0.28	-0.91	0.621	0.85	1	4976	tags=33%, list=28%, signal=45%
294	DESCARTES_FETAL_CEREBRUM_VASCULAR_ENDOTHELIAL_CELLS	443	-0.23	-0.9	0.762	0.892	1	2202	tags=13%, list=12%, signal=14%
295	MANNO_MIDBRAIN_NEUROTYPES_HNPROG	199	-0.25	-0.89	0.7	0.894	1	3198	tags=19%, list=18%, signal=22%
296	HE_LIM_SUN_FETAL_LUNG_C4_CD4_T_CELL	30	-0.32	-0.89	0.626	0.892	1	1761	tags=20%, list=10%, signal=22%
297	AIZARANI_LIVER_C13_LSECS_2	259	-0.24	-0.89	0.737	0.89	1	2169	tags=14%, list=12%, signal=15%
298	DESCARTES_FETAL_HEART_LYMPHOID_CELLS	85	-0.27	-0.89	0.669	0.89	1	3950	tags=26%, list=22%, signal=33%
299	DESCARTES_FETAL_CEREBRUM_SKOR2_NPSR1_POSITIVE_CELLS	48	-0.3	-0.89	0.63	0.889	1	807	tags=8%, list=5%, signal=9%
300	HAY_BONE_MARROW_CD8_T_CELL	55	-0.29	-0.89	0.659	0.888	1	3955	tags=29%, list=22%, signal=37%
301	TRAVAGLINI_LUNG_BASOPHIL_MAST_1_CELL	260	-0.24	-0.88	0.748	0.897	1	4822	tags=30%, list=27%, signal=41%
302	DESCARTES_MAIN_FETAL_NEUROENDOCRINE_CELLS	28	-0.32	-0.88	0.671	0.91	1	845	tags=7%, list=5%, signal=7%
303	GAO_LARGE_INTESTINE_ADULT_CI_MESENCHYMAL_CELLS	304	-0.23	-0.88	0.788	0.909	1	5010	tags=28%, list=28%, signal=38%
304	DESCARTES_FETAL_HEART_MEGAKARYOCYTES	81	-0.27	-0.88	0.707	0.908	1	699	tags=9%, list=4%, signal=9%
305	AIZARANI_LIVER_C10_MVECS_1	252	-0.24	-0.88	0.774	0.908	1	3212	tags=18%, list=18%, signal=21%
306	DURANTE ADULT OLFACTORY NEUROEPITHELIUM VASCULAR SMC	84	-0.27	-0.87	0.703	0.912	1	858	tags=8%, list=5%, signal=9%
307	DESCARTES_MAIN_FETAL_MESOTHELIAL_CELLS	21	-0.34	-0.87	0.635	0.912	1	4499	tags=29%, list=25%, signal=38%
308	MENON_FETAL_KIDNEY_10_IMMUNE_CELLS	64	-0.28	-0.87	0.701	0.914	1	2955	tags=22%, list=17%, signal=26%
309	DESCARTES_FETAL_ADRENAL_VASCULAR_ENDOTHELIAL_CELLS	111	-0.26	-0.87	0.72	0.911	1	800	tags=6%, list=5%, signal=7%
310	DESCARTES_FETAL_STOMACH_STROMAL_CELLS	46	-0.29	-0.87	0.668	0.911	1	3303	tags=22%, list=19%, signal=27%
311	ZHONG_PFC_C1_NEUROD1_POS_EXCITATORY_NEURON	24	-0.33	-0.87	0.649	0.91	1	534	tags=8%, list=3%, signal=9%
312	DESCARTES_FETAL_STOMACH_VASCULAR_ENDOTHELIAL_CELLS	27	-0.32	-0.87	0.66	0.908	1	1669	tags=11%, list=9%, signal=12%
313	CUI_DEVELOPING_HEART_C4_ENDOTHELIAL_CELL	140	-0.25	-0.86	0.734	0.91	1	3167	tags=20%, list=18%, signal=24%
314	DESCARTES_FETAL_SPLEEN_AFP_ALB_POSITIVE_CELLS	167	-0.24	-0.86	0.772	0.912	1	3440	tags=20%, list=19%, signal=25%
315	DURANTE ADULT OLFACTORY NEUROEPITHELIUM MATURE NEURO	176	-0.24	-0.86	0.773	0.911	1	2533	tags=16%, list=14%, signal=19%
316	FAN_EMBRYONIC_CTX_BIG_GROUPS_BRAIN_ENDOTHELIAL	344	-0.23	-0.86	0.828	0.913	1	2643	tags=15%, list=15%, signal=18%
317	VANGURP_PANCREATIC_GAMMA_CELL	43	-0.29	-0.86	0.709	0.91	1	255	tags=5%, list=1%, signal=5%
318	LAKE_ADULT_KIDNEY_C23_ENDOTHELIAL_CELLS_AVR	105	-0.26	-0.86	0.758	0.911	1	3212	tags=23%, list=18%, signal=28%
319	HE LIM SUN FETAL LUNG C3 INTERMEDIATE LYMPHATIC ENDO C	357	-0.23	-0.86	0.835	0.909	1	3973	tags=24%, list=22%, signal=30%
320	GAUTAM EYE IRIS CILIARY BODY COL9A1 HIGH CILIARY BODY CI	89	-0.26	-0.86	0.755	0.908	1	2630	tags=16%, list=15%, signal=18%
321	TRAVAGLINI_LUNG_IGSF21_DENDRITIC_CELL	40	-0.29	-0.86	0.679	0.905	1	130	tags=8%, list=1%, signal=8%
322	TRAVAGLINI_LUNG_ALVEOLAR_EPITHELIAL_TYPE_1_CELL	359	-0.23	-0.85	0.857	0.916	1	3380	tags=22%, list=19%, signal=26%
323	DESCARTES_FETAL_INTESTINE_MYELOID_CELLS	177	-0.23	-0.85	0.783	0.914	1	1728	tags=12%, list=10%, signal=14%
324	HE_LIM_SUN_FETAL_LUNG_C3_OMD_POS_ENDOTHELIAL_CELL	322	-0.22	-0.85	0.86	0.913	1	2673	tags=14%, list=15%, signal=17%
325	MURARO_PANCREAS_PANCREATIC_POLYPEPTIDE_CELL	140	-0.24	-0.85	0.795	0.912	1	4163	tags=29%, list=23%, signal=37%
326	HE_LIM_SUN_FETAL_LUNG_C0_LATE_AIRWAY_SMC_CELL	107	-0.25	-0.85	0.772	0.911	1	4068	tags=26%, list=23%, signal=34%
327	FAN_OVARY_CL16_LYMPHATIC_ENDOTHELIAL_CELL	213	-0.23	-0.84	0.812	0.912	1	3363	tags=24%, list=19%, signal=29%
328	DESCARTES_FETAL_EYE_VASCULAR_ENDOTHELIAL_CELLS	81	-0.26	-0.84	0.749	0.914	1	800	tags=7%, list=5%, signal=8%
329	DESCARTES_FETAL_CEREBELLUM_SLC24A4_PEX5L_POSITIVE_CELL	30	-0.31	-0.84	0.713	0.913	1	1246	tags=10%, list=7%, signal=11%
330	DESCARTES_FETAL_PLACENTA_LYMPHOID_CELLS	115	-0.24	-0.83	0.794	0.926	1	3955	tags=22%, list=22%, signal=28%
331	HE_LIM_SUN_FETAL_LUNG_C4_CD8_T_CELL	43	-0.28	-0.83	0.736	0.924	1	3413	tags=23%, list=19%, signal=29%
332	HE_LIM_SUN_FETAL_LUNG_C1_GHRL_POS_NE_PRECURSOR_CELL	392	-0.22	-0.83	0.891	0.921	1	2825	tags=16%, list=16%, signal=18%
333	MANNO_MIDBRAIN_NEUROTYPES_HPROGFPL	301	-0.22	-0.83	0.865	0.922	1	3353	tags=21%, list=19%, signal=26%
334	DESCARTES_MAIN_FETAL_DUCTAL_CELLS	50	-0.27	-0.83	0.744	0.92	1	6152	tags=44%, list=35%, signal=67%
335	TRAVAGLINI_LUNG_ARTERY_CELL	126	-0.24	-0.83	0.81	0.919	1	3893	tags=23%, list=22%, signal=29%
336	DESCARTES_MAIN_FETAL_SLC26A4_PAEP_POSITIVE_CELLS	213	-0.23	-0.83	0.865	0.919	1	3369	tags=21%, list=19%, signal=26%
337	CUI_DEVELOPING_HEART_TRABECULAR_ATRIAL_CARDIOMYOCYTE	165	-0.23	-0.83	0.849	0.918	1	3130	tags=19%, list=18%, signal=23%
338	AIZARANI_LIVER_C9_LSECS_1	286	-0.22	-0.82	0.878	0.921	1	1812	tags=11%, list=10%, signal=12%
339	HE_LIM_SUN_FETAL_LUNG_C1_PULMONARY_NE_PRECURSOR_CELL	284	-0.22	-0.82	0.884	0.92	1	1005	tags=7%, list=6%, signal=7%

340	DURANTE_ADULT_OLFACTORY_NEUROEPITHELIUM_MONOCYTES	32	-0.29	-0.82	0.745	0.926	1	3402	tags=25%, list=19%, signal=31%
341	GAO_SMALL_INTESTINE_24W_C7_SECRETORY_PROGENITOR	38	-0.28	-0.82	0.738	0.924	1	6436	tags=37%, list=36%, signal=58%
342	DESCARTES_FETAL_INTESTINE_MESOTHELIAL_CELLS	61	-0.26	-0.82	0.774	0.922	1	3247	tags=23%, list=18%, signal=28%
343	HE_LIM_SUN_FETAL_LUNG_C3_ARTERIAL_ENDOTHELIAL_CELL	259	-0.22	-0.82	0.892	0.92	1	2401	tags=14%, list=14%, signal=16%
344	DESCARTES_FETAL_MUSCLE_LYMPHATIC_ENDOTHELIAL_CELLS	58	-0.26	-0.81	0.78	0.933	1	2031	tags=10%, list=11%, signal=12%
345	AIZARANI_LIVER_C29_MVECS_2	297	-0.21	-0.81	0.914	0.93	1	3268	tags=19%, list=18%, signal=23%
346	TRAVAGLINI_LUNG_CD8_MEMORY_EFFECTOR_T_CELL	22	-0.31	-0.81	0.742	0.929	1	3955	tags=45%, list=22%, signal=58%
347	DURANTE_ADULT_OLFACTORY_NEUROEPITHELIUM_PLASMA_CELLS	15	-0.34	-0.81	0.719	0.927	1	1970	tags=20%, list=11%, signal=22%
348	AIZARANI_LIVER_C12_NK_NKT_CELLS_4	40	-0.27	-0.8	0.769	0.939	1	3901	tags=30%, list=22%, signal=38%
349	MANNO_MIDBRAIN_NEUROTYPES_HRGL2C	302	-0.21	-0.8	0.926	0.94	1	2656	tags=15%, list=15%, signal=17%
350	DESCARTES_MAIN_FETAL_ASTROCYTES	33	-0.28	-0.79	0.774	0.942	1	787	tags=9%, list=4%, signal=9%
351	DESCARTES_FETAL_PLACENTA_IGFBP1_DKK1_POSITIVE_CELLS	104	-0.24	-0.79	0.859	0.946	1	3003	tags=17%, list=17%, signal=21%
352	DURANTE_ADULT_OLFACTORY_NEUROEPITHELIUM_B_CELLS	29	-0.28	-0.77	0.792	0.963	1	2914	tags=17%, list=16%, signal=21%
353	CUI_DEVELOPING_HEART_LEFT_VENTRICULAR_CARDIOMYOCYTE	37	-0.27	-0.77	0.803	0.962	1	4460	tags=32%, list=25%, signal=43%
354	DESCARTES_MAIN_FETAL_MICROGLIA	123	-0.23	-0.77	0.902	0.963	1	1535	tags=11%, list=9%, signal=11%
355	GAO_SMALL_INTESTINE_24W_C9_ENTEROENDOCRINE_CELL	44	-0.25	-0.76	0.846	0.969	1	14	tags=2%, list=0%, signal=2%
356	LAKE_ADULT_KIDNEY_C11_THIN_ASCENDING_LIMB	157	-0.22	-0.76	0.917	0.97	1	5149	tags=32%, list=29%, signal=44%
357	ZHENG_CORD_BLOOD_C1_PUTATIVE_MEGAKARYOCYTE_PROGENIT	94	-0.23	-0.76	0.892	0.967	1	3842	tags=27%, list=22%, signal=34%
358	DESCARTES_FETAL_EYE_GANGLION_CELLS	32	-0.27	-0.76	0.837	0.966	1	520	tags=6%, list=3%, signal=6%
359	DESCARTES_MAIN_FETAL_THYMOCYTES	70	-0.23	-0.76	0.862	0.967	1	3560	tags=20%, list=20%, signal=25%
360	DESCARTES_FETAL_LUNG_LYMPHOID_CELLS	113	-0.22	-0.76	0.916	0.965	1	3955	tags=23%, list=22%, signal=29%
361	DESCARTES_MAIN_FETAL_BIPOLAR_CELLS	46	-0.25	-0.75	0.848	0.966	1	1962	tags=9%, list=11%, signal=10%
362	DESCARTES_MAIN_FETAL_SATELLITE_CELLS	47	-0.25	-0.75	0.844	0.966	1	1760	tags=11%, list=10%, signal=12%
363	DESCARTES_FETAL_LIVER_MEGAKARYOCYTES	113	-0.22	-0.75	0.918	0.968	1	3384	tags=18%, list=19%, signal=22%
364	HE_LIM_SUN_FETAL_LUNG_C2_GMP_CELL	32	-0.26	-0.74	0.825	0.968	1	2489	tags=13%, list=14%, signal=15%
365	DESCARTES_MAIN_FETAL_BRONCHIOLAR_AND_ALVEOLAR_EPITHEL	30	-0.27	-0.74	0.845	0.966	1	4389	tags=23%, list=25%, signal=31%
366	DESCARTES_FETAL_STOMACH_LYMPHATIC_ENDOTHELIAL_CELLS	18	-0.3	-0.74	0.833	0.967	1	5439	tags=44%, list=31%, signal=64%
367	DESCARTES_FETAL_SPLEEN_MEGAKARYOCYTES	87	-0.23	-0.74	0.911	0.967	1	3384	tags=18%, list=19%, signal=23%
368	DESCARTES_FETAL_PANCREAS_LYMPHOID_CELLS	110	-0.22	-0.74	0.92	0.966	1	3955	tags=23%, list=22%, signal=29%
369	GAO_SMALL_INTESTINE_24W_C2_PROCRPOS_PROGENITOR	22	-0.28	-0.73	0.85	0.965	1	5610	tags=45%, list=32%, signal=66%
370	ZHONG_PFC_C4_UNKNOWN_INP	75	-0.22	-0.72	0.913	0.972	1	3117	tags=21%, list=18%, signal=26%
371	HE_LIM_SUN_FETAL_LUNG_C2_BASOPHIL_CELL	95	-0.22	-0.72	0.934	0.973	1	4334	tags=23%, list=24%, signal=30%
372	DESCARTES_MAIN_FETAL_LYMPHOID_CELLS	106	-0.21	-0.71	0.943	0.976	1	3872	tags=20%, list=22%, signal=25%
373	ZHONG_PFC_C3_ASTROCYTE	343	-0.18	-0.7	0.995	0.982	1	3485	tags=16%, list=20%, signal=20%
374	DURANTE_ADULT_OLFACTORY_NEUROEPITHELIUM_MAST_CELLS	71	-0.22	-0.69	0.928	0.985	1	3901	tags=24%, list=22%, signal=31%
375	TRAVAGLINI_LUNG_VEIN_CELL	35	-0.24	-0.68	0.93	0.99	1	29	tags=3%, list=0%, signal=3%
376	HE_LIM_SUN_FETAL_LUNG_C7_PCP4_POS_NEURON_CELL	51	-0.22	-0.68	0.933	0.989	1	815	tags=6%, list=5%, signal=6%
377	DESCARTES_FETAL_LIVER_LYMPHOID_CELLS	92	-0.2	-0.67	0.967	0.99	1	3215	tags=15%, list=18%, signal=18%
378	DESCARTES_FETAL_CEREBELLUM_ASTROCYTES	117	-0.19	-0.67	0.985	0.989	1	1512	tags=8%, list=9%, signal=8%
379	ZHONG_PFC_C3_MICROGLIA	445	-0.17	-0.66	1	0.99	1	4733	tags=23%, list=27%, signal=31%
380	DESCARTES_MAIN_FETAL_SATB2_LRRC7_POSITIVE_CELLS	82	-0.2	-0.66	0.974	0.988	1	1048	tags=5%, list=6%, signal=5%
381	FAN_EMBRYONIC_CTX_ASTROCYTE_1	75	-0.19	-0.62	0.986	0.998	1	1823	tags=9%, list=10%, signal=10%
382	DESCARTES_FETAL_CEREBRUM_ASTROCYTES	151	-0.17	-0.62	0.995	0.997	1	1312	tags=6%, list=7%, signal=6%
383	HE_LIM_SUN_FETAL_LUNG_C0_INTERM_CHONDROCYTE	66	-0.19	-0.6	0.991	0.999	1	3650	tags=14%, list=21%, signal=17%
384	HE_LIM_SUN_FETAL_LUNG_C0_MYOFIBROBLAST_2_CELL	86	-0.18	-0.58	0.999	0.999	1	1823	tags=8%, list=10%, signal=9%
385	HE_LIM_SUN_FETAL_LUNG_C3_HMOX1_POS_PRIMITIVE_ERYTHROBL	18	-0.23	-0.57	0.962	0.998	1	4433	tags=28%, list=25%, signal=37%
386	DESCARTES_FETAL_EYE_RETINAL_PIGMENT_CELLS	62	-0.17	-0.54	0.995	0.998	1	4058	tags=16%, list=23%, signal=21%
387	GAO_LARGE_INTESTINE_24W_C6_SECRETORY_PROGENITOR	16	-0.2	-0.48	0.99	0.999	1	721	tags=6%, list=4%, signal=7%

Supplemental Table 3. GO biological processes for SOX2 activated or repressed direct transcriptional targets.

GO BIOLOGICAL PROCESSES COMPLETE FOR DIRECT ACTIVATED SOX2 TRANSCRIPTIONAL TARGETS

	GO BIOLOGICAL PROCESS COMPLETE	+/-	Fold Enrichment	P-value
1	Retinal Cone Cell Development	+	8.48	2.82E-04
2	Retinal Cone Cell Differentiation	+	7.88	3.86E-04
3	Serine Family Amino Acid Metabolic Process	+	5.25	7.86E-05
4	Camera-type Eye Photoreceptor Cell Differentiation	+	4.47	4.92E-04
5	Regulation of Transcription from RNA polymerase II Promoter in Response to Stress	+	4.47	4.92E-04
6	Endoderm Formation	+	4.35	5.81E-04
7	Regulation of Neuron Projection Regeneration	+	4.35	5.81E-04
8	Eye Photoreceptor Cell Development	+	4.14	9.80E-05
9	Alpha-Amino Acid Biosynthetic Process	+	4.01	1.45E-04
10	Regulation of Protein Autophosphorylation	+	4.00	5.24E-04
11	Amino Acid Biosynthetic Process	+	3.79	1.27E-04
12	Negative Regulation of Osteoblast Diffferentiation	+	3.55	6.74E-04
13	Eye Photoreceptor Cell Differentiation	+	3.55	6.74E-04
14	Response to Hydrogen Peroxide	+	3.43	3.75E-05
15	Cellular Response to Glucose Starvation	+	3.43	8.66E-04
16	Regulation of Heart Growth	+	3.13	2.56E-04
17	Positive Regualtion of Gliogenesis	+	2.96	6.76E-04
18	Protein Dephosphorylation	+	2.83	1.93E-05
19	Secondary Alcohol Metabolic Process	+	2.68	1.74E-04
20	Regulation of Gliogenesis	+	2.68	1.74E-04
21	Epidermal Cell Differentiation	+	2.66	8.73E-06
22	Amino Acid Metabolic Process	+	2.65	6.82E-07
23	Alpha-Amino Acid Metabolic Process	+	2.64	4.21E-04
24	Keratinocyte Differentiation	+	2.63	3.20E-04
25	Dephosphorylation	+	2.60	7.49E-06
26	Camera-type Eye Morphogenesis	+	2.59	3.07E-04
27	Response to Reactive Oxygen Species	+	2.55	1.62E-04
28	Response to Calcium Ion	+	2.52	5.15E-04
29	Eye Morphogenesis	+	2.42	2.66E-04
30	Carboxylic Acid Biosynthetic Process	+	2.39	2.54E-05

GO BIOLOGICAL PROCESSES COMPLETE FOR DIRECT REPRESSED SOX2 TRANSCRIPTIONAL TARGETS

	GO BIOLOGICAL PROCESS COMPLETE	+/-	Fold Enrichment	P-value
1	Glutamine Family Amino Acid Biosynthetic Process	+	8.41	8.14E-04
2	Dosage Compensation by Inactivation of X Chromosome	+	7.72	3.66E-04
3	Sex-Chromosome Dosage Compensation	+	6.91	5.96E-04
4	Atrioventricular Valve Morphogenesis	+	5.89	4.71E-04
5	Peptide Cross-Linking	+	5.30	3.43E-04
6	Response to Dexamethasone	+	5.28	8.20E-04
7	Negative Regulation of Osteoclast Differentiation	+	4.80	2.80E-04
8	Ventricular Septum Morphogenesis	+	4.19	6.70E-04
9	Positive Regulation of Epithelial Cell Apoptotic Process	+	4.10	7.65E-04
10	Fibroblast Proliferation	+	3.77	7.08E-04
11	Heterochromatin Organization	+	3.72	2.32E-05
12	Positive Regulation of Fat Cell Differentiation	+	3.44	7.77E-04
13	Heterochromatin Formation	+	3.43	2.75E-04
14	Maintenance of Protein Location in Cell	+	3.39	8.63E-04
15	Cardiac Septum Morphogenesis	+	3.24	7.29E-04
16	Transforming Growth Factor Beta Receptor Signaling Pathway	+	3.22	2.86E-04
17	Ventricular Septum Development	+	3.20	8.03E-04
18	Negative Regulation of Myeloid Cell Differentiation	+	3.16	3.45E-04
19	T Cell Proliferation	+	3.00	5.44E-04
20	Regulation of Potassium Ion Transport	+	2.97	5.93E-04
21	Regulation of DNA replication	+	2.92	3.02E-04
22	Odontogenesis	+	2.85	5.67E-04

23	Cellular Response to Transforming Growth Factor Beta Stimulus	+	2.82	2.87E-04
24	Regulation of Nucleocytoplastmic Transport	+	2.80	3.11E-04
25	Transforming Growth Factor Beta Receptor Superfamily Signaling Pathway	+	2.77	5.05E-05
26	Transmembrane Receptor Protein Serine/Threonine Kinase Signaling Pathway	+	2.73	2.77E-05
27	Response to Transforming Growth Factor Beta	+	2.71	4.23E-04
28	Regulation of Transforming Growth Factor Beta Receptor Signaling Pathway	+	2.64	5.70E-04
29	Regulation of Cellular Response to Transforming Growth Factor Beta Stimulus	+	2.58	7.07E-04
30	Negative Regulation of Kinase Activity	+	2.53	1.65E-04

Supplemental Table 4. GO biological processes based on the differentially expressed proteins in the squamocolumnar junctional glands of control and *Sox2*$^{\Delta/\Delta}$ animals.

GO BIOLOGICAL PROCESSES COMPLETE DECREASED IN *Sox2*$^{\Delta/\Delta}$ SQJX GLANDS COMPARED TO CONTROL

	GO BIOLOGICAL PROCESS COMPLETE	+/-	Fold Enrichment	P-value
1	Bundle of His Cell-Purkinje Myocyte Adhesion Involved in Cell Communication	+	> 100	3.27E-05
2	Cardiac Muscle Cell-Cardiac Muscle Cell Adhesion	+	> 100	4.57E-05
3	Peptide Cross-Linking	+	> 100	1.09E-09
4	Intermediate Filament Organization	+	71.06	1.62E-13
5	Keratinization	+	67.74	3.01E-10
6	Intermediate Filament Cytoskeleton Organization	+	56.69	1.05E-12
7	Intermediate Filament-Based Process	+	56.10	1.14E-12
8	Keratinocyte Differentiation	+	49.71	4.14E-15
9	Establishment of Skin Barrier	+	47.58	3.40E-05
10	Epidermal Cell Differentiation	+	31.72	3.90E-13
11	Skin Development	+	28.12	2.78E-16
12	Epidermis Development	+	21.12	1.84E-12
13	Epithelial Cell Differentiation	+	13.53	3.21E-12
14	Supramolecular Fiber Organization	+	10.30	1.31E-07
15	Epithelium Development	+	7.19	7.75E-09
16	Cytoskeleton Organization	+	4.99	4.92E-05
17	Tissue Development	+	4.68	1.16E-06
18	Animal Organ Development	+	3.40	7.29E-06
19	Cell Differentiation	+	2.77	4.25E-05
20	Cellular Developmental Process	+	2.77	4.27E-05

GO BIOLOGICAL PROCESSES COMPLETE INCREASED IN *Sox2*$^{\Delta/\Delta}$ SQJX GLANDS COMPARED TO CONTROL

	GO BIOLOGICAL PROCESS COMPLETE	+/-	Fold Enrichment	P-value
1	Negative Regulation of Protein K48-Linked Deubiquitination	+	27.24	1.35E-03
2	Carnitine Metabolic Process, CoA-Linked	+	27.24	4.93E-05
3	Epidermal Growth Fator Catabolic Process	+	27.24	1.35E-03
4	Uropod Organization	+	27.24	1.35E-03
5	Negative Regulation of Mitochondrial RNA Catabolic Process	+	27.24	1.35E-03
6	Negative Regulation of Ubiquitin-Specific Protease Activity	+	27.24	1.35E-03
7	ADP Biosynthetic Process	+	27.24	4.93E-05
8	Fumarate Metabolic Process	+	27.24	4.93E-05
9	Glyoxylate Cycle	+	27.24	1.35E-03
10	Cellular Detoxification of Hydrogen Peroxide	+	27.24	1.35E-03
11	Detoxification of Hydrogen Peroxide	+	27.24	4.93E-05
12	miRNA Transcription	+	27.24	1.35E-03
13	Positive Regulation of Processing in Phagocytic Vesicle	+	27.24	1.35E-03
14	Regulation of Protein Processing in Phagocytic Vesicle	+	27.24	1.35E-03
15	Cellular Response to Methylglyoxal	+	27.24	1.35E-03
16	Calcium Ion Transport From Cytosol to Endoplasmic Reticulum	+	27.24	1.35E-03
17	Glutamate Catabolic Process to 2-Oxoglutarate	+	27.24	1.35E-03
18	Glutamate Catabolic Process to Aspartate	+	27.24	1.35E-03
19	Nuclear Pore Localization	+	21.79	8.75E-06
20	Glyceraldehyde-3-Phosphate Biosynthetic Process	+	20.43	2.31E-11

Supplemental Table 5. Gene set enrichment analysis of the differentially expressed proteins in the squamocolumnar junctional glands of control and *Sox2*$^{\Delta/\Delta}$ animals

GSEA Report for *Sox2*$^{\Delta/\Delta}$ vs Control Squamocolumnar Junction Proteins

TISSUE Gene sets enriched in *Sox2*$^{\Delta/\Delta}$ Squamocolumnar Junctions

	GS	SIZE	ES	NES	NOM p-val	FDR q-val	FWER p-val	RANK AT MAX	LEADING EDGE
1	STOMACH	16	0.73	2.76	0	0	0	165	tags=81%, list=20%, signal=100%
2	TONGUE	79	0.41	2.41	0	0	0	189	tags=49%, list=23%, signal=58%
3	HEARTMUSCLE	44	0.4	2.08	0	0.003	0.014	186	tags=50%, list=23%, signal=61%
4	KIDNEY	9	0.66	2.01	0.001	0.005	0.032	184	tags=78%, list=23%, signal=99%
5	SKELETALMUSCLE	104	0.31	1.95	0	0.007	0.055	189	tags=40%, list=23%, signal=46%
6	PANCREAS	12	0.56	1.89	0.006	0.01	0.086	194	tags=67%, list=24%, signal=86%
7	LUNG	3	0.78	1.59	0.02	0.073	0.546	15	tags=33%, list=2%, signal=34%
8	GALLBLADDER	3	0.75	1.57	0.028	0.075	0.597	120	tags=67%, list=15%, signal=78%
9	CHOROID	11	0.39	1.3	0.168	0.305	0.985	129	tags=45%, list=16%, signal=53%
10	EPIDIDYMIS	3	0.64	1.29	0.163	0.289	0.986	295	tags=100%, list=36%, signal=156%
11	PITUITARYGLAND	1	0.93	1.24	0.132	0.33	0.996	53	tags=100%, list=7%, signal=107%
12	PARATHYROID	3	0.55	1.13	0.279	0.463	1	180	tags=67%, list=22%, signal=85%
13	ADRENALGLAND	11	0.32	1.07	0.384	0.527	1	304	tags=55%, list=37%, signal=86%
14	BONEMARROW	14	0.28	1	0.473	0.601	1	318	tags=57%, list=39%, signal=92%
15	THYROID	1	0.65	0.87	0.668	0.763	1	285	tags=100%, list=35%, signal=154%
16	INTESTINE	31	0.16	0.77	0.727	0.861	1	184	tags=26%, list=23%, signal=32%
17	TESTIS	13	0.21	0.76	0.765	0.83	1	295	tags=46%, list=36%, signal=71%
18	FALLOPIANTUBE	4	0.29	0.66	0.919	0.891	1	244	tags=50%, list=30%, signal=71%

TISSUE Gene sets enriched in Control Squamocolumnar Junctions

	GS	SIZE	ES	NES	NOM p-val	FDR q-val	FWER p-val	RANK AT MAX	LEADING EDGE
1	SKIN	21	-0.9	-5.06	0	0	0	43	tags=90%, list=5%, signal=93%
2	VAGINA	8	-0.91	-3.2	0	0	0	19	tags=88%, list=2%, signal=89%
3	ESOPHAGUS	27	-0.49	-3.05	0	0	0	91	tags=63%, list=11%, signal=69%
4	PLACENTA	6	-0.7	-2.07	0.003	0.005	0.011	249	tags=100%, list=31%, signal=143%
5	RETINA	5	-0.77	-2.06	0	0.005	0.012	192	tags=100%, list=24%, signal=130%
6	CERVIX	3	-0.96	-1.97	0	0.01	0.031	40	tags=100%, list=5%, signal=105%
7	ADIPOSE	5	-0.68	-1.83	0.015	0.023	0.081	267	tags=100%, list=33%, signal=148%
8	BLADDER	4	-0.7	-1.65	0.028	0.053	0.19	247	tags=100%, list=30%, signal=143%
9	OVARY	3	-0.67	-1.47	0.077	0.13	0.454	268	tags=100%, list=33%, signal=149%
10	BRAIN	18	-0.27	-1.43	0.078	0.141	0.519	477	tags=89%, list=59%, signal=210%
11	BREAST	2	-0.78	-1.37	0.094	0.164	0.616	179	tags=100%, list=22%, signal=128%
12	SALIVARYGLAND	7	-0.41	-1.33	0.143	0.184	0.697	240	tags=86%, list=29%, signal=121%
13	SEMINALVESICLE	4	-0.47	-1.15	0.257	0.365	0.929	435	tags=100%, list=53%, signal=214%
14	SMOOTHMUSCLE	7	-0.32	-0.99	0.458	0.586	0.986	558	tags=100%, list=69%, signal=315%
15	LIVER	65	-0.08	-0.83	0.674	0.832	0.999	423	tags=71%, list=52%, signal=136%
16	LYMPHOID	13	-0.16	-0.72	0.841	0.955	0.999	204	tags=54%, list=25%, signal=71%
17	ENDOMETRIAM	3	-0.31	-0.65	0.901	0.982	1	560	tags=100%, list=69%, signal=319%
18	PROSTATE	2	-0.36	-0.62	0.925	0.949	1	520	tags=100%, list=64%, signal=276%

Supplemental Table 6. Primary antibody information

Application	Antibody	Company	Catalog Number	Concentration
Human Barrett's esophagus biopsy and organoid staining	SOX2	Santa Cruz	sc365823	1:50 (biopsy) 1:25 (organoid)
	CDX2	Abcam	Ab76541	1:200
Mouse tissue and organoid staining				
Immunofluorescence	SOX2	Abcam	ab97959	1:100
	mCherry	Abcam	ab125096	1:200
	Ki67	Life Tech	14-5698-80	1:200
	CK13	Abcam	ab92551	1:200
	CK7	Abcam	ab181598	1:500
	Das-1 (IgM)	Gift of Jeff Brown and Koushik Das, Washington University School of Medicine (Main References 102-104)		1:100
Immunohistochemistry	SOX2	Santa Cruz	sc365823	1:50
	TP63	Ventana	5867061001	1:5
	Ki67	Life Tech	14-5698-80	1:200
	CK13	Abcam	ab92551	1:200
	CK14	Abcam	ab7800	1:100
	CK5	Abcam	ab52635	1:100
	γ-H2AX	Cell Signaling	9718S	1:100
	mCherry	Abcam	ab125096	1:150
	CD8	Cell Signaling	98941	1:200
	STING	Cell Signaling	13657	1:500
	F4/80	Cell Signaling	70076	1:200
	Ly6G	Cell Signaling	87048S	1:200
